# Facile and Scalable Route to Access Rare Deoxy Amino Sugars for Nonulosonic Acid Aldolase Biosynthesis

**DOI:** 10.3389/fchem.2022.865026

**Published:** 2022-06-09

**Authors:** Yixuan Zhou, Kuo-Shiang Liao, Shiou-Ting Li, Chung-Yi Wu

**Affiliations:** Genomics Research Center, Academia Sinica, Taipei, Taiwan

**Keywords:** azide, double inversion, amino sugar, biocatalytic, nonulosonic acid

## Abstract

We presented a facile and scalable route for the synthesis of di-azido sugars *via* one-pot double inversion of the mono-benzoyl sugars by TBAN_3_ and studied the dependency pattern between solvent and protecting groups as well as the configuration of the neighboring and leaving groups. Moreover, we developed a chemical synthetic strategy for pseudaminic acid precursors (11 steps in 49%). Furthermore, we discussed the configuration of nonulosonic acid precursors for specificity of PseI and PmNanA enzymatic synthesis, permitting us to synthesize new nonulosonic acid derivatives for accessing Pse isomers.

## Introduction

Since azide-containing sugar was first reported by Bertho (1930), various methods have been developed to introduce azides to different positions of a sugar molecule. Published in 1967, the Mitsunobu reaction provided a simple access to organic azides from alcohols by reacting primary and secondary alcohols with hydrogen azide, triphenylphosphine, and diethyl azodicarboxylate (DEAD) (Hughes, 2004). As substrates, secondary alcohols are of considerable interest because they invert stereochemistry in the reaction. Although the Mitsunobu reaction is useful in the synthesis of glycosamine derivatives, it has not been widely applied in the synthesis of glycosamine derivatives due to toxicity of hydrazoic acid and potential explosivity of DEAD (Armarego, 2017). On the other hand, S_*N*_2 displacement can invert stereochemistry by external nucleophiles in carbohydrate sulfonate derivatives; however, S_*N*_2 displacement is a complex reaction system and can be complicated by the stereo-electronic effect (Smith, 2020), steric effect (Hamlin et al., 2018a), and solvent effect (Hamlin et al., 2018b).

In S_*N*_2 displacement, a common route to stereocenter inversion in carbohydrate chemistry involves triflation of a given hydroxyl group followed by a substitution using a variety of nucleophilic reagents. Ramström’s laboratory developed an efficient carbohydrate stereocenter inversion based on glycoside triflate displacement by acetate (OAc^−^)/thioacetate (SAc^−^)/nitrite (NO_2_^−^) under very mild conditions (Pei et al., 2005; Dong et al., 2006; Dong et al., 2007). They demonstrated that neighboring equatorial ester groups played highly important roles in this type of reaction, inducing the inversion reaction to proceed efficiently and resulting in good yields. Generally, thioacetate (SAc^−^) is soft, nonbasic, and highly nucleophilic; acetate (OAc^−^) is significantly more basic and less nucleophilic. Different from the aforementioned nucleophilic anions, the azide anion sits at the borderline, inclining toward competing elimination (Hale et al., 2014). Kulkarni and co-workers developed a general and divergent protocol to access differentially protected rare deoxy amino L-sugar building blocks that can be used as glycosyl donors or acceptors in the assembly of complex bacterial glycans in one-pot double serial or double parallel displacements (stereocenter inversion) of 1,3-diol f L-sugars by the NaN_3_/DMF system (Emmadi and Kulkarni, 2013b; a; c; 2014; Clark et al., 2016; Sanapala and Kulkarni, 2016b; a; Behera and Kulkarni, 2018; Emmadi and Kulkarni, 2018). Based on this chemical strategy, Fascione and co-workers established an efficient chemoenzymatic method to prepare pseudaminic acid, which is a nonmammalian sugar present on the cell surface of a number of bacteria, including *Pseudomonas aeruginosa*, *Campylobacter jejuni*, and *Acinetobacter baumannii* (Flack et al., 2020).

Interestingly, Ramström’s group found that solvents played an important role in neighboring group participation and remote group participation (NGP/RGP) (Pei et al., 2005; Dong et al., 2007). For anti-diequatorial configuration between triflate and ester functionalities, NGP/RGP was more favored in a polar solvent and less favored in a nonpolar solvent. Moreover, Sanapala and Kulkarni (2016a) obtained the double inversion products in moderate yield (47–69%) in the NaN_3_/DMF system even when using HMPA (dipole moment at 25°C: 5.37D) instead of DMF (dipole moment at 25°C: 3.82D) as the solvent under harsh conditions at 110°C to increase the nucleophilicity. Their experimental data strongly suggested that the NGP and inversion reactions were in strong competition.

In order to advance azide-based carbohydrate double inversion, we studied regioselective one-pot double displacements (stereocenter inversion) of 1,2/1,3-diol trifluoromethanesulfonates (OTf, triflate) of sugars by the tetrabutylammonium azide (TBAN_3_)/toluene system and obtained a group of non-natural di-azido sugars in excellent yield under mild conditions. These results promoted us to further explore substrate specificity of PseI and PmNanA nonulosonic acid precursors with differing configurations to obtain a new Pse isomer as a potential Pse transference inhibitor.

## Materials and Methods

### General Methods

All chemicals were purchased as reagent grade and used without further purification. Anhydrous dichloromethane (CH_2_Cl_2_) was purchased from a commercial source without further distillation. Pulverized MS 4Å (Aldrich) for glycosylation was activated by heating at 350°C for 10 h. Reactions were monitored by analytical thin-layer chromatography (TLC) in EM silica gel 60 F254 plates and visualized under UV (254 nm) and/or by staining with acidic ceric ammonium molybdate or *p*-anisaldehyde. Flash chromatography was performed on a silica gel (Merck) of 40–63 μm particle size. ^1^H NMR spectra were recorded on a Bruker AVANCE 600 (600 MHz) spectrometer at 25°C. Chemical shifts (in ppm) were assigned relative to CDCl_3_ (δ = 7.24 ppm) and D_2_O (δ = 4.80 ppm). ^13^C NMR spectra were obtained using a Bruker AVANCE 600 spectrometer and were calibrated with CDCl_3_ (δ = 77.00 ppm). Coupling constants (*J*) are reported in hertz (Hz). Splitting patterns are described by using the following abbreviations: s, singlet; brs, broad singlet; d doublet; brd, broad doublet; t, triplet; q, quartet; dt, doublet of triplets; tt, triplet of triplets; qt, quartet of triplets; m, multiplet. High-resolution ESI-TOF was recorded on a Bruker Daltonics spectrometer.

### General Acylation Procedure

Benzyl *α*-L-fucopyranoside (100 mg, 0.4 mmol) and dibutyltin oxide (107 mg, 0.432 mmol) were dissolved in 30 ml of methanol and refluxed for 2 h. After evaporation of the solvent, the residue was dried under vacuum and then dissolved in 2.5 ml of toluene. After the solution was cooled to 0°C for 5 min, a solution of acetic anhydride (0.432 mmol, 40.8 μL) in anhydrous DCM (0.1 ml) was added dropwise and then allowed to react at room temperature for 8 h. The resulting mixture was directly purified by flash column chromatography, yielding the acetylated products.

### General Benzoylation Procedure

Benzyl *α*-L-fucopyranoside (1.0 g, 4 mmol) and dibutyltin oxide (1.07 g, 4.32 mmol) were dissolved in 300 ml of methanol and refluxed for 2 h. After evaporation of the solvent, the residue was dried under vacuum and then dissolved in 25 ml of solvent (toluene oracetonitrile). After the solution was cooled to 0°C for 5 min, a solution of benzoyl chloride (4.32 mmol, 502 μL) in anhydrous DCM (1 ml) was added dropwise and then allowed to react at room temperature for 8 h. The resulting mixture was directly purified by flash column chromatography, yielding the benzoylated products.

### General Synthesis of Triflate Derivatives

To a solution of the suitably *O*-protected benzyl glycoside carrying two unprotected OH groups at C2/C3, C2/C4, or C3/C4 (0.2 g, 0.6 mmol) in DCM (5 ml) was added pyridine (0.269 ml, 3.35 mmol) at -20°C. Trifluoromethanesulfonic anhydride (0.281 ml, 1.67 mmol) in DCM (1 ml) was added dropwise, and the mixture was stirred while allowing to warm from -20 to 10°C for over 2 h. The resulting mixture was subsequently diluted with DCM (50.0 ml) and washed with 1 M HCl (10.0 ml), aqueous NaHCO_3_ (10.0 ml), water (10.0 ml), and brine (10.0 ml). The organic phase was dried over Na_2_SO_4_ and concentrated in vacuum at low temperatures. The residue was used directly in the next step without further purification.

### General Inversion of Triflate Derivatives

TBAN_3_/TBANO_2_ (5.0 equiv.) was all added at once to a solution of the protected triflate residue in dry toluene (5 ml) at 25°C. After stirring at room temperature (25°C) for 8 h, the mixture was diluted with EtOAc (50.0 ml) and washed with water (10.0 ml). The organic phase was dried with MgSO_4_ and concentrated in vacuum. Purification of the residue by flash column chromatography (10:1, hexanes/ethyl acetate) afforded the inversion products.

### General Deprotection of the Di-Azido Derivatives

To a solution of compound **31** (100 mg, 0.3 mmol) in DCM (5.0 ml) was added pydine (79.5 μL, 0.987 mmol) at 0°C. After the solution was cooled to 0°C for 5 min, a solution of benzoyl chloride (0.493 mmol, 57.3 μL) in anhydrous DCM (0.1 ml) was added dropwise and then allowed to react at room temperature for 8 h. The resulting mixture was subsequently diluted with DCM (50.0 ml) and washed with 1 M HCl (10.0 ml), aqueous NaHCO_3_ (10.0 ml), water (10.0 ml), and brine (10.0 ml). The organic phase was dried over Na_2_SO_4_ and concentrated in vacuum at low temperatures. The residue was used directly in the next step without further purification. The benzoylated residue was dissolved in pyridine (1.5 ml), and thioacetic acid (3.0 ml) was added.(Khedri et al., 2014). The reaction was allowed to stir at room temperature for 12 h and monitored by TLC. The reaction mixture was concentrated in high vacuum to remove the pyridine and thioacetic acid. The residue was run through a short silica gel column to remove the lower polarity impurities (EA/Hexane, 1/4 to 1/1). The residue was concentrated and directly dissolved in MeOH (10 ml), and then NaOMe (20 mg) was added. The reaction was allowed to stir at room temperature for 6 h. After the benzoyl group had been removed completely, the reaction was neutralized by IR120, filtered, and concentrated in vacuum. Purification of the residue by flash column chromatography afforded 84 mg of the di-acetamido product **S2** with 76% yield. To the solution of **S2** in MeOH (10 ml) was added 10% Pd-C (84 mg). The resulting mixture was hydrogenated on a hydrogenation apparatus for 12 h under a hydrogen atmosphere (2-3 psi). The resulting suspension was filtered and concentrated. Silica gel column purification (DCM/MeOH = 10:1, by column) produced the desired product **40** (59 mg, 97% yield).

### General Enzymatic Reaction Based on PmNanA

The reaction was carried out in 2 ml Tris–HCl buffer (100 mM, pH 7.5) containing compound **40** (10 mg), sodium pyruvate (5.0 equiv.), and PmNanA (*P. multocida* sialic acid synthase) (Li et al., 2008) (0.2 mg) by incubating at 37°C for 12 h with agitation (140 rpm). The product formation was monitored by thin-layer chromatography (eluent: butanol: AcOH: H_2_O = 5 : 3: 2) (Supplementary Figure S1). When no additional product formation was observed, the reaction was quenched by adding an equal volume of cold 95% EtOH and incubating on ice for 30 min to precipitate the protein followed by centrifugation to remove the precipitates. The supernatant was concentrated by rotary evaporation, and the product was purified using a C-18 column.

### General Enzymatic Reaction Based on NeuB3

The reaction was carried out in 2 ml Tris–HCl buffer (150 mM, pH 7.5) containing compound **40** (10 mg), phosphoenolpyruvate (5.0 equiv.), MgCl_2_ (20 mM), and NeuB3 (pseudaminic acid synthase) (Chou et al., 2005) (0.2 mg) by incubating at 37°C for 15.5 h with agitation (140 rpm). The product formation was monitored by thin-layer chromatography (eluent: butanol: AcOH: H_2_O = 5: 3: 2) (Supplementary Figure S1). When no additional product formation was observed, the reaction was quenched by adding an equal volume of cold 95% EtOH and incubating on ice for 30 min to precipitate the protein followed by centrifugation to remove the precipitates. The supernatant was concentrated by rotary evaporation, and the product was purified using a C-18 column.

## Results and Discussion

### Synthesis of Rare Deoxy Amino L/D-Sugar

**SCHEME 1:**
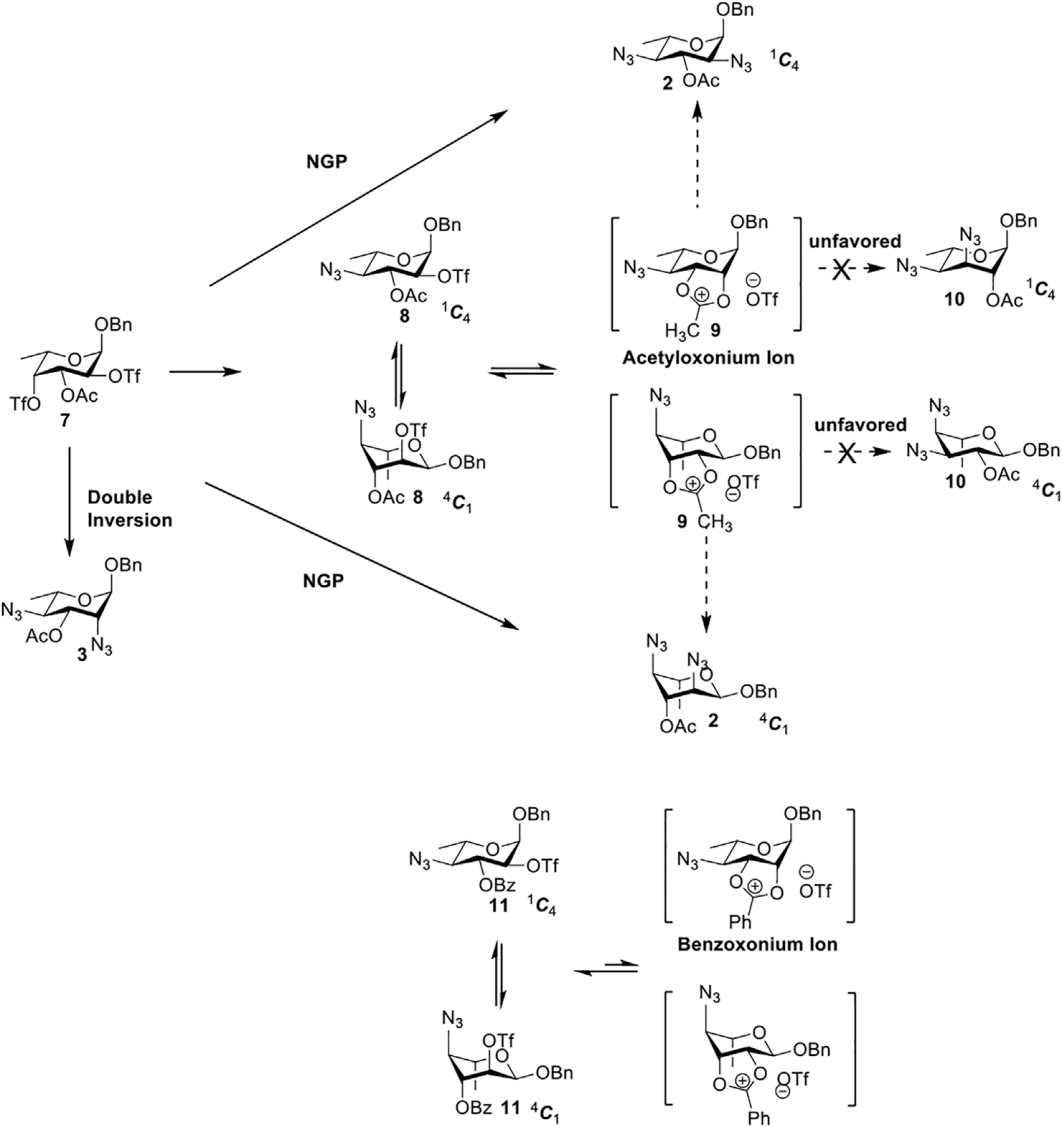
Competition of NGP with double inversion.

Previous studies on inversion reactions in the synthesis of 2,4-di-azido-2,4-di-deoxy-*α*-L-Rha recommended harsh conditions (110°C in DMF/HMPA); however, the yield was not satisfactory (58%/69%), and no detailed explanations were given (Sanapala and Kulkarni, 2016a; Flack et al., 2020). In our study, using 3-*O*-acetyl-*α*-L-Fuc **1** as reactant and TBAN_3_ (5.0 equiv.×2) in the double inversion process resulted in a mixture of 2,4-di-azido-3-*O*-acetyl-2,4,6-trideoxy-*α*-L-Glu **2** and 2,4-di-azido-3-*O*-acetyl-2,4-di-deoxy-*α*-L-Rha **3** (Table 1, Entry 1, NMR ratio *51*/*49*, **2**: **3**). To our surprise, a better result was obtained when 3-*O*-benzoyl-*α*-L-Fuc **4** was used instead as the reactant. In this case, 2,4-di-azido-3-*O*-benzoyl-2,4-di-deoxy-*α*-L-Rha **6** was the major product of the process (Table 1, Entry 3, NMR ratio *38*/*62*, **5**: **6**). We think the main reason for this behavior is most likely due to the formation of competing acetoxonium ion intermediate **9** in the reaction pathway (Scheme 1). Specifically, when the triflates of compound **8** was shifted far toward the ^4^ *C*_1_ chair form, an anti-di-axial relationship is established between the ester and the triflate group at the 2-position, and the undesirable **9** forms quickly to compete with the attacking azide anion in a nonpolar solvent. For the ^1^ *C*_4_ chair form of compound **8**, the acetoxonium ion intermediate **9** also could be obtained slowly. In this case, nucleophilic attack on the acetyloxonium ion from the equatorial/axial 2-position is preferred, opening the five-member-ring to produce L-Glu **2**. Attack at the equatorial/axial 3-position appeared to be unfavored because no 2-*O*-acetyl-3,4-di-azido-3,4,6-tri-deoxy-*α*-L-Alt **10** was identified in the reaction. Moreover, the anti-di-equatorial configuration between the triflate and ester is less likely to produce an intermediate benzoxonium ion leading to the double inversion (DI) as the major instead of neighboring group participation (NGP). For substrate **7**, reaction with 4-OTf does not have any complicating side reaction. Furthermore, acetyloxonium ion intermediate is unlikely to form because of the 3,4-*cis* configuration.

**TABLE 1:** Competition of NGP, double inversion, and *β*-elimination.

Entry	Substrate	Products					
		NGP		DI		*β*-E	
1	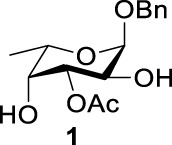	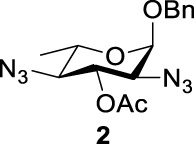	51%^a^	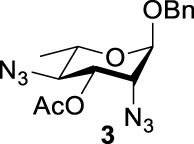	49%^a^	—	—
2	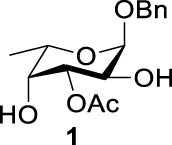	—	—	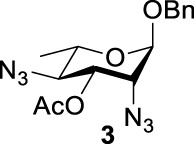	95%^b^	—	—
3	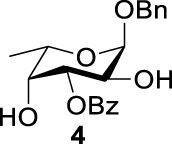	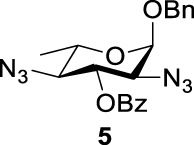	38%^a^	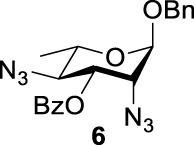	62%^a^	—	—
4	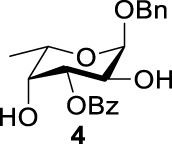	—	—	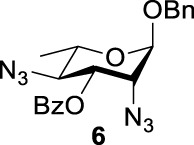	95%^c^	—	—
5	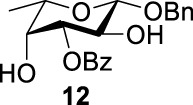	—	—	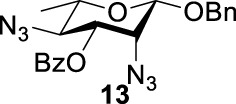	90%^c^	—	—
6	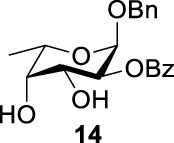	—	—	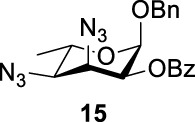	80%^c^	—	—
7	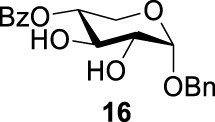	—	—	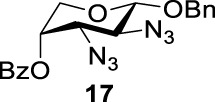	23%^c^	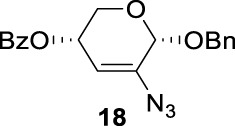	50%^c^
8	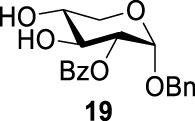	—	—	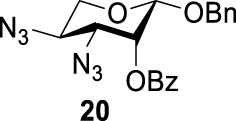	16%^c^	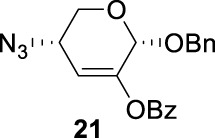	78%^c^
9	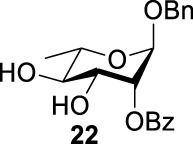	—	—	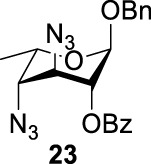	35%^c^	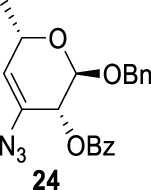	37%^c^
10	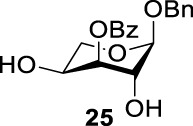	—	—	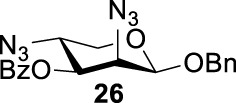	79%^c^	—	—
11	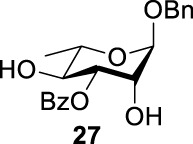	Complex mixture^c^	—	—	—	—	—
12	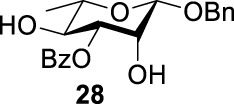	—	—	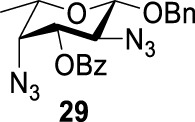	85%^c^	—	—

a Reagents and conditions. i: Tf_2_O, pyridine, and DCM, -20°C, 1 h. ii: TBAN_3_, 5.0 eq. × 2 and toluene, r.t., 6 h. NMR ratio.

b i: Tf_2_O, pyridine, and DCM, -20°C, 1 h. ii: TBAN_3_, 20.0 eq. × 2 and toluene, r.t., 6 h. Isolated yield.

c i: Tf_2_O, pyridine, and DCM, -20°C, 1 h. ii: TBAN_3_, 8.0 eq. × 2 and toluene, r.t., 6 h. Isolated yield.

Ramström’s group concluded that the neighboring group’s participation would compete with inversion when ester protecting groups were used (Pei et al., 2005). The formation of acetoxonium ion intermediate **9** is slower in a nonpolar solvent (toluene) than in a polar solvent (DMF), favoring an intermolecular S_*N*_2 reaction (NGP), which is dependent on the nucleophile concentration. As we expected, the yield of **3** was dramatically increased when the concentration of TBAN_3_ was increased close to the saturation limit (20.0 equiv. × 2), resulting in near quantitative formation of **3** (Table 1, Entry 2, 95% isolated yield). Furthermore, in contrast to the high nucleophile concentration for the double inversion of *α*-L-Fuc **1** in toluene, the *α*-L-Rha **6** was formed almost quantitatively for *α*-L-Fuc **4** in TBAN_3_ (8.0 equiv. × 2, Table 1, Entry 4, which was less than half of the saturation limit, 20.0 × 2 equiv.). Even when the gram-scale reaction of 3-*O*-benzoyl *α*-L-Fuc **4** (1g/3g/5 g) was performed, this double inversion strategy still managed a high yield (95%/93%/90%) of *α*-L-Rha **6**. In the 5 g scale, the intermediate product 2-*O*-triflate-3-*O*-benzoyl-4-azido-2,4,6-tri-deoxy *α*-L-Glu **11** was isolated in 5% yield, agreeing with what we proposed in Scheme 1. As a result, the neighboring group participation (NGP) was avoided, and the overall yield increased.

**SCHEME 2:**
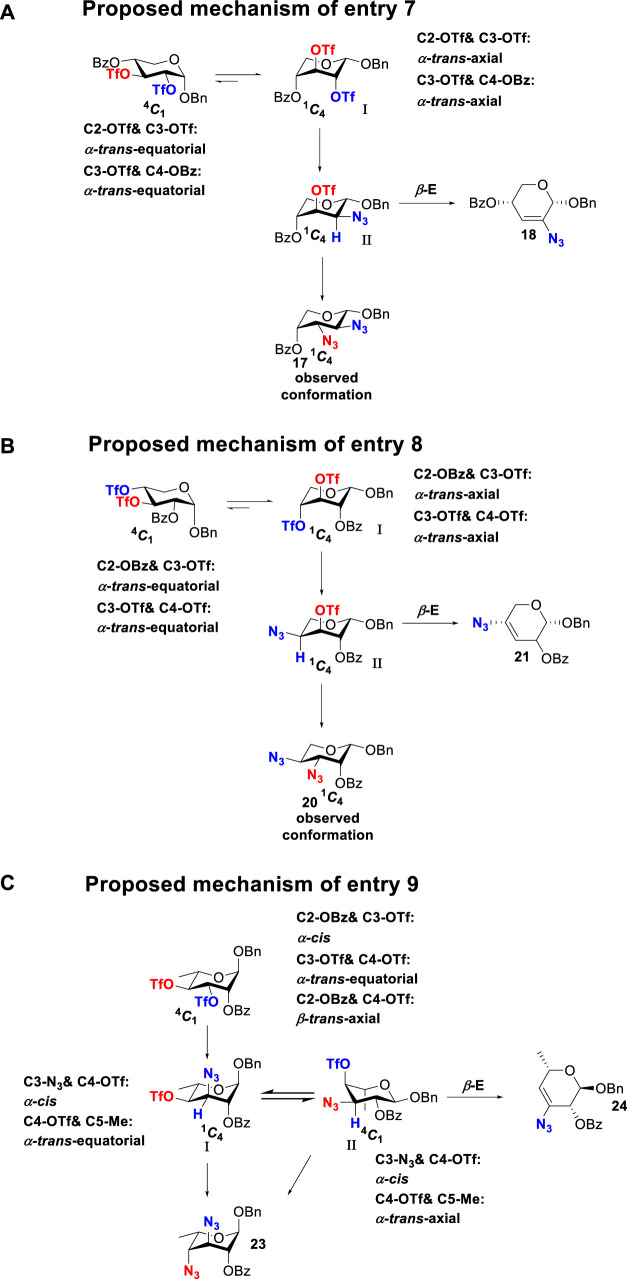
Conformation-dependent double inversion.

To further analyze these findings and explore how reactivity is affected by configurations of triflate and the neighboring ester group, we designed other inversion systems (Table 1). We prepared a range of compounds by protecting one of the hydroxyl groups in the 2-, 3-, or 4-position with a benzoyl group and subsequently tested in the azide-based double inversion reactions. Our experimental results (Table 1) indicated that a range of configurations provided high yields of diazide products. Exceptions can be found in entries 7–9 wherein the precursor vicinal diol bears the trans-di-equatorial configuration. This may be the result of initial S_N_2 displacement followed by conformational shift and antiperiplanar elimination. Scheme 2 depicts plausible pathways to elimination products. In addition, Entry 11 provided poor results.

In Scheme 2, we discussed completely the mechanisms of entries 7–9. All of these involve vicinal di-equatorial triflates with a neighboring benzoyl group. The Vicinal Triflate Effect (Hale et al., 2014) had been used to explain the reactivity of the inversion reaction, which suggested an *α*-*trans*-relationship between C (2/3/4-carbon)-triflates and a vicinal axially oriented strong electron-withdrawing group, indicating poor S_*N*_2 displacements. Based on the Vicinal Triflate Effect, when the conformation shifted, the *α*-*trans*-relationship between C (3)-OTf and the benzoyl group was formatted, which led to antiperiplanar elimination (Schemes 2A,B). For Entry 9, when the conformation shifted, the intermediate **II** could not format the *α*-*trans*-relationship between C (4)-OTf and the neighboring group (Scheme 2C). Meanwhile, the C (3)-proton in the *α-trans-*axial relationship with C (4)-triflate led to an antiperiplanar elimination reaction, which competed with the triflate S_*N*_2 displacement, and resulted in product **24**.

More interestingly, we thought compound **25** was likely to form a *α*-*trans*-axial relationship between the C (2)-triflate and the C (3)-benzoyl group, which would perform poorly in S_*N*_2 displacement. However, the double inversion product **26** was obtained in good yield (79%), suggesting that the ^4^ *C*_1_ chair conformation was exclusively populated for the intermediate of **25** triflate. Such conformation usually engages in S_*N*_2 displacement readily.

As we discussed previously, the double inversion reaction proceeded smoothly when the ester group was in the *α-cis* or *α-trans-*equatorial relationship with the triflate groups (compounds **4** and **12**). However, for *α*-L-Rha **27** and *β*-L-Rha **28**, only the *β*-isomer **28** provided double inversion product **29** in excellent yield (85%), whereas the *α*-isomer proved inefficient. Despite the fact, we are still unclear about how anomeric configuration influences the S_*N*_2 transition state.

### Chemical Synthesis of 6-Deoxyl-L-AltdiNAc

**SCHEME 3:**
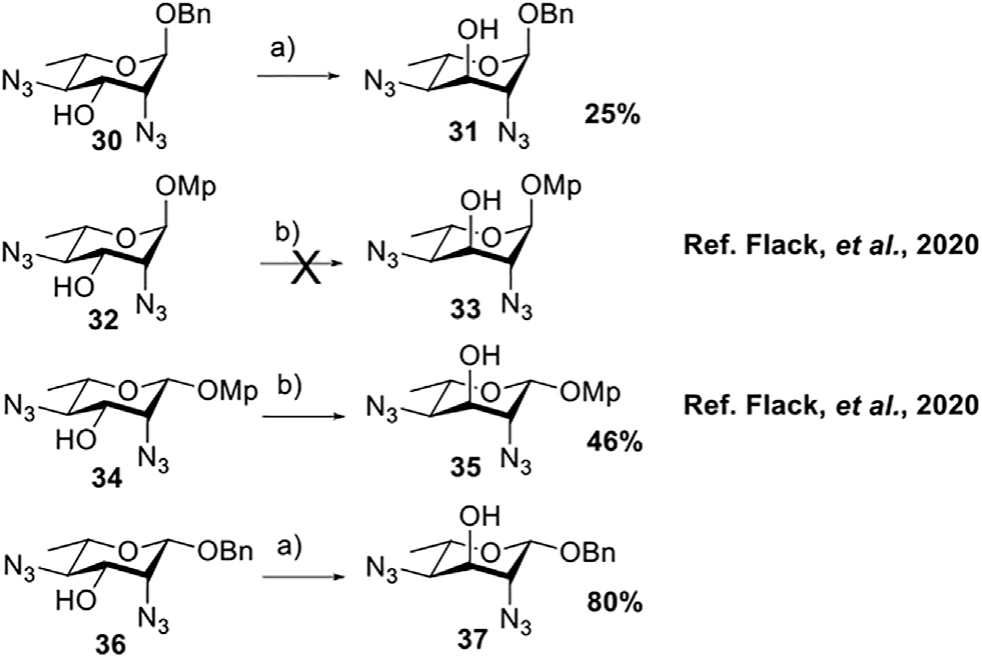
Solvent-dependent nitrite-mediated carbohydrate epimerization.^*a*^(a) Tf_2_O, pyridine, and DCM, -20°C, 2 h; (ii) TBANO_2_and toluene, r.t., 6 h. (b) Tf_2_O, pyridine, and DCM, 0°C; (ii) TBANO_2_ and CH_3_CN, 70°C (Flack et al., 2020).

**SCHEME 4:**
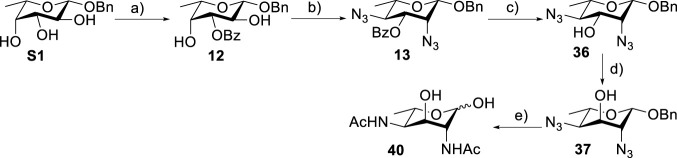
Chemical synthesis of 6-deoxyl-L-AltdiNAc 40.^*a*^(a) (i) Bu_2_SnO (1.1 eq.) and MeOH, 70°C, 2 h; (ii) BzCl, TBAB, and toluene, 0–22°C, 6 h, 96% over two steps. (b) (i) Tf_2_O, pyridine, and DCM, -20°C, 2 h; (ii) TBAN_3_ and toluene, r. t., 6 h, 90% over two steps. (c) NaOMe and MeOH, 95%. (d) Tf_2_O, pyridine, and DCM, -20°C, 2 h; (ii) TBANO_2_ and toluene, r.t., 6 h, 80% over 2 steps. (e) (i) BzCl, pyridine, and DCM, 0–22°C, 6 h; (ii) HSAc and pyridine, r.t. overnight; (iii) NaOMe, MeOH, (ⅳ) Pd(OH)_2_, and H_2_, 6 h, 74% over four steps.

To prepare di-NAc sugars to obtain nonulosonic acid precursors, we need to synthesize 6-deoxy-L-AltdiNA **40** (Scheme 4). Fascione’s group used the Lattrell–Dax reaction (Guo and O'Doherty, 2007; 2008) with *β*-Rha **34** as the substrate and found that the epimerization product **35** was obtained in moderate yield in the TBANO_2_/acetonitrile system (46%) (Flack et al., 2020). They also found *α*-Rha **32** was inefficient for the reaction. Considering these unsatisfactory results, we tested *α*-Rha **30** and *β*-Rha **36** in the TBANO_2_/toluene system. As we expected, *β*-Rha **36** gave *β*-Alt **37** in excellent yield (80%) over two steps, and *α*-Rha **30** provided the epimerization product *α*-Alt **31** in moderate yield (25%) (Scheme 3).

**FIGURE 1:**
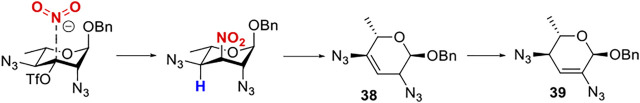
N-attack of nitrite to achieve epimerization of the substrates.

For these results, usually, a simple explanation is that axially disposed OBn and developing 1,3-diaxial interactions retard the approach of nucleophile with *α*-Rha **30** and **32** but not with *β*-Rha **34** and **36**, especially a significant increase in productivity based on solvent effection. Thus, compounds **34** and **36**, both with *β*-anomer, yielded compound **35** (46%) in acetonitrile and compound **37** (80%) in toluene. For compound **30**, both inversed nitro-product and nitrite-ester were formed by N/O-attack of nitrite to achieve epimerization of the substrates without a neighboring equatorial ester group. In this case (Figure 1), the nitro-product was unstable and subsequently underwent the antiperiplanar elimination reaction to form double-bond product **39** (in 70% yield).

Based on our discussions on neighboring group participation and solvent effect, we provided a new chemical synthetic strategy to prepare 6-deoxy-L-AltdiNAc **40** with 49% overall yield (Scheme 4). Compared with the 11% overall yield reported by Fascione’s group, our route provides a more efficient chemical synthesis strategy to obtain the important pseudaminic acid precursor **40** for enzymatic synthesized Pse.

### Enzymatic Synthesis by Pseudaminic Acid Synthase and Sialic Acid Synthase

The pseudaminic acid was expressed by *Pseudomonas* species, which exhibited the same connectivity as legionaminic acid (Leg) but in a different stereochemical configuration. Like Neu5Ac, Pse and Leg are well-characterized microbial nonulosonic acids (NulO). Enzymatic synthesis of NulOs generally proceeds by the aldol reaction of a 6-carbon monosaccharide with 3-carbon pyruvate, activation to a CMP-sugar, and incorporation into glycans. Previous studies showed the condensation of phosphoenolpyuvate with *N*-acetyl-D-mannosamine (ManNAc) was catalyzed by sialic acid synthase to form sialic acid and phosphate (Blacklow and Warren, 1962; Masson and Holbein, 1983; Angata and Varki, 2002). In particular, PmNanA, a sialic acid synthase, exhibited good tolerance toward mannose derivatives in ManNAc modifications (Li et al., 2008; Khedri et al., 2014). In addition, a study also characterized enzymes of pseudaminic acid enzymatic synthesis (Schoenhofen et al., 2006). However, not many studies have been carried out on the conversion of non-natural modified AltdiNAc derivatives by a pseudaminic acid aldolase (PseI)-catalyzed reaction.

Based on the experience from the enzymatic synthesis of the non-natural C-5-, C-7-, and C-8-modified pseudaminic acid derivatives, we can synthesize new, desired isomers of pseudaminic acid for further research. To date, none of the enzymes for enzymatic synthesis of nonulosonic acid precursors have been studied in the aspect of configurations. In order to explore the sialidase substrate specificity for various configurations and to synthesize new nonulosonic acid derivatives enzymatically for further research, we chose compounds **6**, **15**, **26**, and **29** for deprotection to obtain **41**, **42**, **43**, and **44.** Individually, the deprotection products were combined with compound **40** and catalyzed by PseI and PmNanA.

For PmNanA, all of diacetamido pyranoside derivatives tested were completely incompatible. Although 2,4-diNAc Lyx **43** and ManNAc share similar configurations, we did not observe effective catalysis of PmNanA. We attributed the ineffectiveness of 2,4-diNAc Lyx **43** to the loss of hydrogen bonding to PmNanA. For PseI, the results (Table 2) indicated the importance of 2- and 4-NHAc group configuration in the reaction. Clearly, Pse (Pse5Ac7Ac) **45** was obtained in excellent yield from 6-deoxy-L-AltdiNAc **40** (in 80% yield). Similar to precursor **40**, the only difference was that compound **41** has opposite configuration of C3-OH. Using substrate **41**, a new nonulosonic acid derivative **46** was obtained in a 61% isolated yield as the isomer of Pse. Different from precursor **40**, compound **44** contains two acetamido groups (on C2 and C4) and one hydroxyl group (on C3) with the opposite configuration of C2, C3, and C4. Similar to compound **44**, **43** (like **40**) only contains C3-OH. For compounds **43** and **44**, although 2- and 4-NHAcs are present, their configuration proved to be inefficient for PseI. For compound **42**, the change of the functional group order will further increase the structural complexity to lose binding interactions with PseI. In sum, we found that both the configuration of di-acetamido groups and the placement of acetamido groups were important for PseI substrate specificity. Under this guideline, including the diverse substitution on the two amino groups, diverse pseudaminic acid derivatives could be accessed to elucidate substrate specificities of bacterial, human, and viral pseudaminic acid synthase.

**TABLE 2:** Reactions catalyzed by pseudaminic acid synthase (PseI) and sialic acid synthase (PmNanA).

Substrate	Enzyme	
	PmNanA^a^	PseI^b^
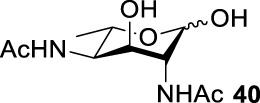	--	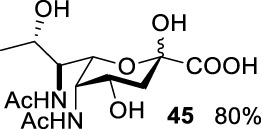
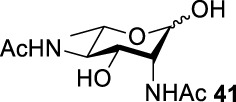	--	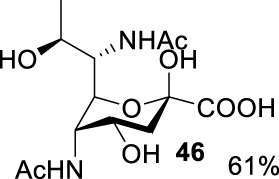
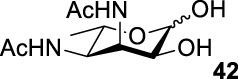	--	--
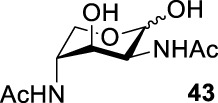	--	--
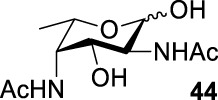	--	--

a Enzymes: PmNanA, *P. multocida P-1059*, sialic acid synthase. The substrate (5 μmol/ml), sodium pyruvate (5.0 equiv.), and PmNanA (0.2 mg) were dissolved in Tris–HCl buffer (100 mM, pH 7.5, 20 mM MgCl_2_). The reaction was kept in 37°C for 12 h with agitating at 140 rpm.

b Enzymes: PseI, *NeuB3-C. jejuni*, Cj1317, pseudaminic acid synthase. The substrate (5 μmol/ml), phosphoenolpyruvate (5.0 equiv.) and NeuB3 (0.2 mg) were dissolved in Tris–HCl buffer (150 mM, pH 7.5, 20 mM MgCl_2_). The reaction was kept in 27°C for 15.5 h with agitating at 140 rpm.

## Conclusion

In summary, we have developed a facile and scalable azide-based double inversion synthesis to prepare rare deoxy amino L/D-sugars using simple mild nucleophilic substitution of triflates with TBAN_3_. We also mapped the potential influence from the adjacent ester protecting group and solvent dependence, and the desired products could be formed in good yield. These strategies and findings are expected to find broad applications in the synthesis of diamino sugars that are widely present in bacterial glycans.

Furthermore, we investigated the configuration of NulO precursors for specificity of PseI and PmNanA enzymatic synthesis. Substrate specificity studies of five sugars indicate that, in general, the configuration of C2 and C4-NHAc of sugars in PseI is important for maintaining the activity of PseI. Fortunately, the RhadiNAc **41**) could be converted enzymatically by PseI to form a new nonulosonic acid as the isomer of Pse. The free hydroxyl group in C3 of 6-deoxy sugar provides us the possibility for installing fluoride, azide, or methoxy moieties *via* readily available methodologies, which could be used in the synthesis of modified Pse derivatives. This ready access to Pse isomer/Pse derivatives and their glycoconjugates will open up unexplored opportunities to study the biological function and significance of bacterial Pse glycosylation in pathogenesis to develop novel therapeutic intervention.

## Data Availability

The original contributions presented in the study are included in the article/Supplementary Material; further inquiries can be directed to the corresponding author.
