# Supplementary material for: Facile and Scalable Route to Access Rare Deoxy Amino Sugars for Nonulosonic Acid Aldolase Biosynthesis
Source: Front Chem. 2022 Jun 9;10:865026. doi: 10.3389/fchem.2022.865026 (PMC9245050; doi:10.3389/fchem.2022.865026)
Supplement: Supplementary file 1 [file DataSheet1.PDF]

Supporting information

Facile and Scalable Route to Access Rare Deoxy Amino Sugars for Nonulosonic Acid Aldolase Biosynthesis

Yixuan Zhou[1], Kuo-Shiang Liao[1], Shiou-Ting Li[1], Chung-Yi Wu[1*]

[1] Genomics Research Center, Academia Sinica, No. 128 Academia Road, Section 2, Nangang District, Taipei 11529, Taiwan.

*** Correspondence:**
Corresponding Author: C-Y. Wu
cyiwu@gate.sinica.edu.tw

Contents

**Characterization of New Compounds**	S2-S15
**NMR spectra for new compounds**	S16-S80
**NMR Ratio of Double Inversion Competed with NGP**	S81-S82
**Reference**	S83

Characterization of New Compounds

Benzyl 3-*O*-acetyl-*α*-L-fucopyranoside (1).

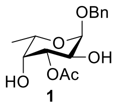

1

Prepared by the same route as general acylation, yielding **1** as a colorless syrup (85 mg, 72%). ^{1}H NMR ($CDCl_3$, 600 MHz): δ 7.39-7.31 (m, 5 H), 5.09-5.06 (m, 1 H), 5.01 (d, 1 H, *J* = 3.8 Hz), 4.74 (d, 1 H, *J* = 11.7 Hz), 4.57 (d, 1 H, *J* = 11.8 Hz), 4.04-4.00 (m, 2 H), 3.84 (s, 1 H), 2.47 (br, 1 H), 2.24 (br, 1 H), 2.15 (s, 3 H), 1.24 (d, 3 H, *J* = 6.6 Hz). ^{13}C NMR ($CDCl_3$, 150 MHz) δ 171.1, 137.0, 128.6, 128.1, 128.1, 98.1, 74.0, 70.7, 70.0, 66.9, 66.1, 21.1, 16.0. HRMS (ESI-TOF, MH^+) calcd for $C_{15}H_{20}O_6H$: 297.1333, found: 297.1330.

Benzyl 3-*O*-acetyl-2,4-di-azido-*α*-L-rhamnopyranoside (3).

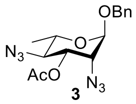

3

^{1}H NMR ($CDCl_3$, 600 MHz) δ 7.41-7.34 (m, 5 H), 5.33 (dd, 1 H, *J* = 3.7 Hz, *J* = 10.1 Hz), 4.82 (d, 1 H, *J* = 1.3 Hz), 4.70 (d, 1 H, *J* = 12.0 Hz), 4.53 (d, 1 H, *J* = 12.1 Hz), 4.07 (dd, 1 H, *J* = 1.6 Hz, *J* = 3.7 Hz), 3.67 (m, 1 H), 3.56 (t, 1 H, *J* = 10.1 Hz, *J* = 10.1 Hz), 2.21 (s, 3 H), 1.36 (d, 3 H, *J* = 6.2 Hz). ^{13}C NMR ($CDCl_3$, 150 MHz) δ 169.9, 136.4, 128.6, 128.2, 128.1, 96.7, 72.4, 69.4, 67.1, 62.6, 61.2, 20.7, 18.3. HRMS (ESI-TOF, MNa^+) calcd for $C_{15}H_{18}N_6O_4Na$: 369.1282, found: 369.1277.

Benzyl 3-*O*-benzoyl-*α*-L-fucopyranoside (4).

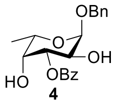

4

Prepared by the same route as general benzoylation in acetonitrile, yielding **4** as a colorless syrup (0.70 g, 50%). ^{1}H NMR (600 MHz, $CDCl_3$) δ 8.19-8.11 (m, 2 H), 7.58 (m, 1 H), 7.47-7.34 (m, 7 H), 5.32 (dd, 1 H, *J* = 3.1 Hz, *J* = 10.3 Hz), 5.07 (d, 1 H, *J* = 4.0 Hz), 4.78 (d, 1 H, *J* = 11.7 Hz), 4.60 (d, 1 H, *J* = 11.7 Hz), 4.18 (dd, 1 H, *J* = 4.0 Hz, *J* = 10.3 Hz), 4.10 (m, 1 H), 3.98 (d, 1 H, *J* = 2.9 Hz), 1.27 (d, 3 H, *J* = 6.6 Hz); ^{13}C NMR ($CDCl_3$, 150 MHz) δ 166.5, 137.0, 133.3, 129.9, 129.7, 128.6, 128.5, 128.1, 128.1, 98.3, 74.7, 70.8, 70.1,

67.0, 66.1, 16.0; HRMS (ESI-TOF, MNa^+) calcd for $C_{20}H_{22}O_6Na$: 381.1309, found: 381.1316.

Benzyl 3-*O*-benzoly-2,4-di-azido-*α*-L-rhamnopyranoside (6).

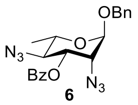

6

^{1}H NMR ($CDCl_3$, 600 MHz) δ 8.16-8.15 (m, 2 H), 7.64 (m, 1 H), 7.52 (m, 2 H), 7.43-7.36 (m, 5 H), 5.61 (dd, 1 H, *J* = 3.7 Hz, *J* = 9.8 Hz), 4.90 (d, 1 H, *J* = 1.5 Hz), 4.75 (d, 1 H, *J* = 12.0 Hz), 4.58 (d, 1 H, *J* = 12.0 Hz), 4.24 (dd, 1 H, *J* = 1.6 Hz, *J* = 3.7 Hz), 3.79-3.72 (m, 2 H), 1.42 (d, 3 H, *J* = 6.0 Hz). ^{13}C NMR ($CDCl_3$, 150 MHz) δ 165.5, 136.4, 133.8, 130.0, 128.8, 128.7, 128.6, 128.2, 128.1, 96.9, ,72.7, 69.5, 67.4, 63.1, 61.4,18.4. HRMS (ESI-TOF, MH^+) calcd for $C_{20}H_{20}N_6O_4NH$:409.1619, found: 409.1614.

Benzyl 6-deoxy-2-*O*-triflate-3-*O*-benzoly-4-azido-*α*-L-glucopyranoside (11).

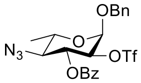

11

^{1}H NMR ($CDCl_3$, 600 MHz) δ 8.10 (m, 2 H), 7.63 (m, 1 H), 7.50 (m, 2 H), 7.43-7.36 (m, 5 H), 5.89 (t, 1 H, *J* = 9.8 Hz, *J* = 9.8 Hz), 5.16 (d, 1 H, *J* = 3.7 Hz), 4.89 (dd, 1 H, *J* = 3.7 Hz, *J* = 10.0 Hz), 4.82 (d, 1 H, *J* = 12.1 Hz), 4.66 (d, 1 H, *J* = 12.1 Hz), 3.90 (m, 1 H), 3.38 (t, 1 H, *J* = 10.0 Hz, *J* = 9.8 Hz), 1.38 (d, 3 H, *J* = 6.2 Hz). ^{13}C NMR ($CDCl_3$, 150 MHz) δ 165.1, 135.7, 133.8, 129.9, 128.7, 128.6, 128.4, 128.2, 121.4, 119.3, 117.1, 115.0, 94.6, 81.7, 70.2, 69.6, 66.6, 66.4, 18.0.

Benzyl 3-*O*-benzoyl-*β*-L-fucopyranoside (12).

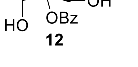

12

Benzyl *β*-L-fucopyranoside (100 mg, 0.4 mmol) and dibutyltin oxide (107 mg, 0.432 mmol) were dissolved in 30 mL of methanol and refluxed for 2 h. After evaporation of the solvent, the residue was dried under vacuum and then dissolved in 2.5 mL of toluene. A solution of The TBAB (1.0 equiv.) in anhydrous DCM (0.1 mL) was added in the reaction and then

allowed to stir at room temperature over 5 min. After the solution was cooled to 0 °C for 5 min, a solution of benzoyl chloride (0.432 mmol, 50.2 μL) in anhydrous DCM (0.1 mL) was added dropwise and then allowed to react at room temperature for 8h. The resulting mixture was directly purified by flash column chromatography, yielding **12** as a colorless syrup (135 mg, 95.9%). ^{1}H NMR (600 MHz, $CDCl_3$) δ 8.12 (m, 2 H), 7.60 (m, 1 H), 7.49-7.33 (m, 7 H), 5.10 (dd, 1 H, *J* = 3.2 Hz, *J* = 10.1 Hz), 5.01 (d, 1 H, *J* = 11.7 Hz), 4.68 (d, 1 H, *J* = 11.6 Hz), 4.49 (d, 1 H, *J* = 7.7 Hz), 4.04 (m, 1 H), 4.00 (dd, 1 H, *J* = 3.2 Hz, *J* = 5.9 Hz), 3.80 (dd, 1 H, *J* = 6.5 Hz, *J* = 13.0 Hz), 2.39 (br, 1 H), 2.07 (br, 1 H), 1.41 (d, 3 H, *J*=6.5 Hz); ^{13}C NMR ($CDCl_3$, 150 MHz) δ 166.2, 137.0, 133.4, 130.0, 129.6, 128.6, 128.5, 128.2, 128.1, 102.1, 76.1,71.1, 70.6, 70.3, 69.4, 16.2; HRMS (ESI-TOF, MNa^+) calcd for $C_{20}H_{22}O_6Na$: 381.1309, found: 381.1314.

Benzyl 3-*O*-benzoly-2,4-di-azide-*β*-L-rhamnopyranoside (13).

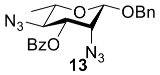

13

^{1}H NMR ($CDCl_3$, 600 MHz) δ 8.14-8.12 (m, 2 H), 7.65 (m, 1 H), 7.52 (m, 2 H), 7.42-7.36 (m, 5 H), 5.05 (dd, 1 H, *J* = 3.7 Hz, *J* = 10.2 Hz), 5.01 (d, 1 H, *J* = 12.0 Hz), 4.70-4.68 (m, 2 H), 4.27 (dd, 1 H, *J* = 0.6 Hz, *J* = 3.6 Hz), 3.68 (t, 1 H, *J* = 9.9 Hz, *J* = 10.0 Hz), 3.34-3.30 (m, 1 H), 1.51 (d, 3 H, *J* = 6.1 Hz). ^{13}C NMR ($CDCl_3$, 150 MHz) δ 165.6, 136.4, 133.9, 130.0, 128.7, 128.6, 128.2, 128.0, 97.5, 74.0, 71.3, 70.6, 62.7, 61.8, 18.4. HRMS (ESI-TOF, MH^+) calcd for $C_{20}H_{20}N_6O_4H$: 409.1619, found: 409.1617.

Benzyl 2-*O*-benzoly-*α*-L-fucopyranoside (14).

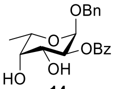

14

Prepared by the same route as general benzoylation in acetonitrile, yielding **14** as a colorless syrup (0.45 g, 32%). ^{1}H NMR ($CDCl_3$, 600 MHz) δ 8.08-8.06 (m, 2 H), 7.59 (m,1 H), 7.45 (m,2 H), 7.33-7.31 (m,2 H), 7.27-7.24 (m, 3 H), 5.28 (dd, 1 H, *J* = 3.8 Hz, *J*=10.2 Hz), 5.18 (d, 1 H, *J* = 3.9 Hz), 4.75 (d, 1 H, *J* = 12.4 Hz), 4.57 (d, 1 H, *J* = 12.3 Hz), 4.26 (dd, 1 H, *J* = 3.4 Hz, *J* = 10.2 Hz), 4.14 (m, 1 H), 3.89 (d, 1 H, *J* = 2.8 Hz), 1.34 (d, 3 H, *J* = 6.7 Hz). ^{13}C NMR ($CDCl_3$, 150 MHz) δ 167.1, 137.5, 133.3, 129.9, 129.6, 128.4, 128.4,

127.8, 127.6, 95.9, 72.5, 72.2, 69.7, 68.9, 65.9, 16.1. HRMS (ESI-TOF, MH^+) calcd for $C_{20}H_{22}O_6H$: 359.1489, found: 359.1485.

Benzyl 2-*O*-benzoly-3,4-di-azide-6-deoxyl-*α*-L-allopyranoside (15).

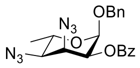

15

^{1}H NMR ($CDCl_3$, 600 MHz) δ 8.14-8.13 (m, 2 H), 7.64 (m, 1 H), 7.52 (m, 2 H), 7.38 (m, 2 H), 7.25 (m, 3 H), 5.22 (t, 1 H, *J* = 4.0 Hz, *J* = 4.0 Hz), 5.12 (d, 1 H, *J* = 4.1 Hz), 4.86 (d, 1 H, *J* = 12.8 Hz), 4.63 (d, 1 H, *J* = 12.7 Hz), 4.47 (t, 1 H, *J* = 3.4 Hz, *J* = 3.4 Hz), 4.20 (m, 1 H), 3.21 (dd, 1 H, *J* = 3.2 Hz, *J* = 9.9 Hz), 1.33 (d, 3 H, *J* = 6.3 Hz). ^{13}C NMR ($CDCl_3$, 150 MHz) δ 165.4, 137.4, 133.8, 130.2, 128.8, 128.6, 128.4, 127.7, 127.4, 94.7, 70.4, 70.0, 62.8, 62.4, 60.0, 17.7. HRMS (ESI-TOF, MNa^+) calcd for $C_{20}H_{20}N_6O_4Na$: 431.1438, found: 431.1447.

Benzyl 4-*O*-benzoly-*α*-D-xylopyranoside (16).

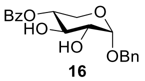

16

Prepared by the same route as general benzoylation in toluene, yielding **16** as a colorless syrup (0.54 g, 38%). ^{1}H NMR ($CDCl_3$, 600 MHz) δ 8.06-8.04 (m, 2 H), 7.58 (m,1 H), 7.45-7.33 (m,7 H), 5.09 (m, 1 H), 4.99 (d, 1 H, *J* = 3.8 Hz), 4.80 (d, 1 H, *J* = 11.8 Hz), 4.56 (d, 1 H, *J* = 11.7 Hz), 4.07 (t, 1 H, *J* = 9.4 Hz, *J* = 9.4 Hz), 3.92 (dd, 1 H, *J* = 5.6 Hz, *J* = 10.8 Hz), 3.71 (t, 1 H, *J* = 10.7 Hz, *J* = 10.8 Hz), 3.66 (m, 1 H), 3.30 (br, 1 H), 2.78 (br, 1 H). ^{13}C NMR ($CDCl_3$, 150 MHz) δ 166.2, 136.8, 133.4, 129.8, 129.5, 128.6, 128.4, 128.2, 128.1, 97.4, 72.7, 72.4, 71.9, 69.8, 59.1. HRMS (ESI-TOF, MH^+) calcd for $C_{19}H_{20}O_6H$: 345.1333, found: 345.1327.

Benzyl 4-*O*-benzoly-2,3-di-azide-*α*-D-arabinopyranoside (17).

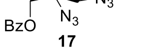

17

^{1}H NMR ($CDCl_3$, 600 MHz) δ 8.12-8.11 (m, 2 H), 7.63 (m, 1 H), 7.50 (m, 2 H), 7.45-7.35 (m, 5 H), 5.36 (s, 1 H), 5.00 (d, 1 H, *J* = 11.8 Hz), 4.75 (d, 1 H, *J* = 11.7 Hz), 4.45 (d, 1 H,

J = 7.7 Hz), 4.28 (dd, 1 H, J = 1.7 Hz, J=13.4 Hz), 3.90 (dd, 1 H, J = 7.7 Hz, J = 10.8 Hz), 3.66 (d, 1 H, J = 13.3 Hz), 3.43 (dd, 1 H, J = 3.4 Hz, J = 10.8 Hz), 1.33 (d, 3 H, J = 6.3 Hz). ^{13}C NMR ($CDCl_3$, 150 MHz) δ 165.7, 136.3, 133.6, 130.0, 129.2, 128.6, 128.6, 128.3, 128.2, 101.3, 71.2, 68.9, 65.0, 62.2, 61.5. HRMS (ESI-TOF, MNa^+) calcd for $C_{19}H_{18}N_6O_4Na$: 417.1282, found: 417.1286.

Benzyl 2-azide-2,3-di-deoxy-3-dehydro-4-*O*-benzoly-*α*-D-lyxopyranoside (18).

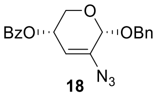

18

^{1}H NMR ($CDCl_3$, 600 MHz) δ 8.06-8.05 (m, 2 H), 7.61 (m, 1 H), 7.49-7.35 (m, 7 H), 5.77 (m, 1 H), 5.66 (d, 1 H, J = 2.6 Hz), 5.04 (s, 1 H), 4.88 (d, 1 H, J = 11.5 Hz), 4.69 (d, 1 H, J = 11.4 Hz), 4.10 (dd, 1 H, J = 6.1 Hz, J = 10.8 Hz), 4.02 (dd, 1 H, J = 9.1 Hz, J = 10.7 Hz). ^{13}C NMR ($CDCl_3$, 150 MHz) δ 166.0, 138.3, 136.7, 133.4, 129.8, 129.5, 128.6, 128.5, 128.2, 112.0, 92.9, 70.5, 66.0, 59.7. HRMS (ESI-TOF, MNa^+) calcd for $C_{19}H_{17}N_3O_4Na$: 374.1111, found: 374.1117.

Benzyl 2-*O*-benzoly-*α*-D-xylopyranoside (19).

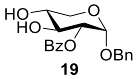

19

Prepared by the same route as general benzoylation in toluene, yielding **19** as a colorless syrup (0.43 g, 31%). ^{1}H NMR ($CDCl_3$, 600 MHz) δ 8.08-8.07 (m, 2 H), 7.61 (m, 1 H), 7.47 (m, 2 H), 7.30-7.21 (m, 5 H), 5.14 (d, 1 H, J = 3.7 Hz), 4.94 (dd, 1 H, J = 3.7 Hz, J = 10.0 Hz), 4.77 (d, 1 H, J = 12.3 Hz), 4.52 (d, 1 H, J = 12.4 Hz), 4.15 (t, 1 H, J = 9.2 Hz, J = 9.3 Hz), 3.84-3.77 (m, 2 H), 3.71 (t, 1 H, J = 10.7 Hz, J = 10.7 Hz), 3.05 (br, 2 H). ^{13}C NMR ($CDCl_3$, 150 MHz) δ 166.5, 137.1, 133.4, 129.9, 129.5, 128.5, 128.4, 127.8, 127.7, 95.5, 73.8, 72.4, 70.6, 69.4, 61.4. HRMS (ESI-TOF, MH^+) calcd for $C_{19}H_{20}O_6Na$: 345.1333, found: 345.1328.

Benzyl 2-*O*-benzoly-3,4-di-azide-*β*-L-lyxopyranoside (20).

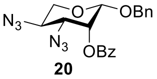

20

^{1}H NMR ($CDCl_3$, 600 MHz) δ 8.13-8.12 (m, 2 H), 7.64 (m, 1 H), 7.50 (m, 2 H), 7.34-7.26 (m, 5 H), 5.61 (t, 1 H, *J* = 3.2 Hz, *J* = 3.1 Hz), 4.91 (d, 1 H, *J* = 2.6 Hz), 4.88 (d, 1 H, *J* = 12.5 Hz), 4.63 (d, 1 H, *J* = 12.6 Hz), 4.28 (dd, 1 H, *J* = 3.2 Hz, *J* = 12.5 Hz), 3.90 (dd, 1 H, *J* = 3.6 Hz, *J* = 6.4 Hz), 3.81 (m, 1 H), 3.51 (dd, 1 H, *J* = 5.7 Hz, *J* = 12.5 Hz). ^{13}C NMR ($CDCl_3$, 150 MHz) δ 165.5, 137.0, 133.6, 130.1, 129.1, 128.5, 128.4, 127.8, 127.5, 96.1, 70.1, 68.7, 60.2, 58.7. HRMS (ESI-TOF, MH^+) calcd for $C_{19}H_{18}N_6O_4H$: 395.1462, found: 395.1457.

Benzyl 2-*O*-benzoly-3,4-di-deoxy-4-azide-4-dehydro-*α*-D-arabinopyranoside (21).

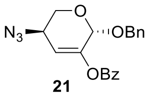

21

^{1}H NMR ($CDCl_3$, 600 MHz) δ 8.13-8.11 (m, 2 H), 7.62 (m, 1 H), 7.49 (m, 2 H), 7.32-7.25 (m, 5 H), 5.70 (m, 1 H), 5.50 (m, 1 H), 5.26 (d, 1 H, *J* = 4.0 Hz), 4.89 (d, 1 H, *J* = 12.3 Hz), 4.68 (d, 1 H, *J* = 12.4 Hz), 4.23 (m, 1 H), 3.98 (m, 1 H). ^{13}C NMR ($CDCl_3$, 150 MHz) δ 166.1, 139.1,137.0, 133.3, 129.9, 129.8, 128.4, 128.0, 127.9, 105.6, 93.8, 70.2, 67.1, 59.6. HRMS (ESI-TOF, MH^+) calcd for $C_{19}H_{17}N_3O_4H$: 352.1292, found: 352.1293.

Benzyl 2-*O*-benzoyl-*α*-L-rhamnopyranoside (22).

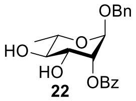

22

Prepared by the same route as general benzoylation in toluene, yielding **22** as a colorless syrup (0.12 g, 8.1%). ^{1}H NMR ($CDCl_3$, 600 MHz) 8.06 (m, 2 H), 7.59 (m, 1 H), 7.45 (m, 2 H), 7.40-7.32 (m, 5 H), 5.40 (dd, 1 H, *J* = 1.7 Hz, *J* = 3.4 Hz), 4.99 (d, 1 H, *J* = 1.3 Hz), 4.75 (d, 1 H, *J* = 11.8 Hz), 4.56 (d, 1 H, *J* = 11.8 Hz), 4.13 (d, 1 H, *J* = 9.4 Hz), 3.81 (m, 1 H), 3.63 (t, 1 H, *J* = 9.5 Hz, *J* = 9.4 Hz), 2.86 (br, 2 H), 1.38 (d, 1 H, *J* = 6.2 Hz). ^{13}C NMR ($CDCl_3$, 150 MHz) δ 166.4, 137.0, 133.5, 129.9, 129.4, 128.5, 128.5, 128.0, 128.0, 97.0, 73.6, 73.0, 70.6, 69.5, 68.3, 17.7. HRMS (ESI-TOF, MH^+) calcd for $C_{20}H_{22}O_6H$: 359.1489, found: 359.1488.

Benzyl 2-*O*-benzoly-3,4-di-azide-6-deoxyl-*α*-L-idopyranoside (23).

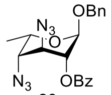

23

^{1}H NMR ($CDCl_3$, 600 MHz) δ 8.12-8.10 (m, 2 H), 7.62 (m, 1 H), 7.50 (m, 2 H), 7.35-7.29 (m, 5 H), 5.23 (dd, 1 H, *J* = 3.4 Hz, *J* = 5.8 Hz), 4.96 (d, 1 H, *J* = 3.4 Hz), 4.80 (d, 1 H, *J* = 12.4 Hz), 4.63 (d, 1 H, *J* = 12.4 Hz), 4.40 (m, 1 H), 4.02 (t, 1 H, *J* = 5.6 Hz, *J* = 5.6 Hz), 3.45 (dd, 1 H, *J* = 3.5 Hz, *J* = 5.4 Hz), 1.34 (d, 3 H, *J* = 6.7 Hz). ^{13}C NMR ($CDCl_3$, 150 MHz) δ 165.3, 137.0, 133.6, 130.0, 129.0, 128.5, 128.5, 127.9, 127.7, 96.7, 69.7, 69.2, 64.4, 61.4, 59.9, 15.9. HRMS (ESI-TOF, MH^+) calcd for $C_{20}H_{20}N_6O_4H$: 409.1619, found: 409.1619.

Benzyl 2-*O*-benzoly-3-azide-3,4,6-tri-deoxy-3-dehydro-*α*-L-altropyranoside (24).

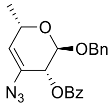

24

^{1}H NMR ($CDCl_3$, 600 MHz) δ 8.12-8.10 (m, 2 H), 7.61 (m, 1 H), 7.48 (m, 2 H), 7.42-7.33 (m, 5 H), 5.66 (d, 1 H, *J* = 1.7 Hz), 5.32 (m, 1 H), 5.16 (s, 1 H), 4.83 (d, 1 H, *J* = 11.9 Hz), 4.69 (d, 1 H, *J* = 11.9 Hz), 4.54 (m, 1 H), 1.43 (d, 3 H, *J* = 6.7 Hz). ^{13}C NMR ($CDCl_3$, 150 MHz) δ 165.9, 137.0, 133.5, 130.6, 130.0, 129.3, 128.6, 128.4, 128.0, 127.9, 119.1, 97.6, 7 70.2, 66.6, 64.2, 20.8. HRMS (ESI-TOF, MH^+) calcd for $C_{20}H_{19}N_3O_4H$: 366.1448, found: 366.1449.

Benzyl 3-*O*-benzoyl-*α*-L-arabinopyranoside (25).

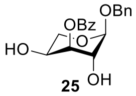

25

Prepared by the same route as general benzoylation in acetonitrile, yielding **25** as a colorless syrup (0.85 g, 59%). ^{1}H NMR ($CDCl_3$, 600 MHz) δ 8.11 (m, 2 H), 7.59 (m, 1 H), 7.46 (m, 2 H), 7.41-7.34 (m, 5 H), 5.36 (dd, 1 H, *J* = 3.2 Hz, *J* = 10.1 Hz), 5.09 (d, 1 H, *J* = 3.8 Hz), 4.81 (d, 1 H, *J* = 11.6 Hz), 4.57 (d, 1 H, *J* = 11.6 Hz), 4.19 (m, 2 H), 3.96 (d, 1 H, *J* = 12.3 Hz), 3.75 (dd, 1 H, *J* = 2.0 Hz, *J* = 12.5 Hz); ^{13}C NMR ($CDCl_3$, 150 MHz) δ 166.4, 136.9, 133.4, 129.9, 129.6, 128.6, 128.5, 128.2, 128.1, 98.5, 73.7, 70.0, 68.2, 67.4,

62.7; HRMS (ESI-TOF, MNH_4H^+) calcd for $C_{19}H_{25}O_6N$: 363.1676, found: 363.1655.

Benzyl 3-*O*-benzoly-2,4-di-azide-*β*-D-lyxopyranoside (26).

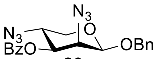

26

^{1}H NMR ($CDCl_3$, 600 MHz) δ 8.16-8.14 (m, 2 H), 7.65 (m, 1 H), 7.53-7.37 (m, 7 H), 5.60 (dd, 1 H, *J* = 3.7 Hz, *J* = 9.7 Hz), 4.91 (d, 1 H, *J* = 2.2 Hz), 4.81 (d, 1 H, *J* = 12.0 Hz), 4.58 (d, 1 H, *J* = 11.9 Hz), 4.22 (dd, 1 H, *J* = 2.3 Hz, *J* = 3.5 Hz), 4.16 (m, 1 H), 3.92 (dd, 1 H, *J* = 5.3 Hz, *J* = 11.5 Hz), 3.70 (t, 1 H, *J* = 11.0 Hz, *J* = 11.0 Hz). ^{13}C NMR ($CDCl_3$, 150 MHz) δ 165.4, 136.3, 133.8, 130.0, 128.8, 128.7, 128.7, 128.3, 128.1, 97.2, 72.5, 69.5, 61.2, 60.1 ,56.6. HRMS (ESI-TOF, MNa^+) calcd for $C_{19}H_{18}N_6O_4Na$: 417.1282, found: 417.1286.

Benzyl 3-*O*-benzoyl-*α*-L-rhamnopyranoside (27).

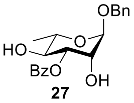

27

Prepared by the same route as general benzoylation in toluene, yielding **27** as a colorless syrup (1.2 g, 85%). ^{1}H NMR ($CDCl_3$, 600 MHz) δ 8.09 (m, 2 H), 7.59 (m, 1 H), 7.47-7.29 (m, 7 H), 5.35 (dd, 1 H, *J* = 3.3 Hz, *J* = 9.4 Hz), 4.90 (d, 1 H, *J* = 1.6 Hz), 4.76 (d, 1 H, *J* = 11.9 Hz), 4.56 (d, 1 H, *J* = 12.0 Hz), 4.20 (m, 1 H), 3.86 (m, 2 H), 1.39 (d, 3 H, *J* = 6.0 Hz); ^{13}C NMR ($CDCl_3$, 150 MHz) δ 167.0, 137.0, 133.5, 129.9, 129.5, 128.5, 128.0, 128.0, 98.6, 75.6, 71.5, 69.9, 69.1, 68.9, 17.6; HRMS (ESI-TOF, MNa^+) calcd for $C_{20}H_{22}O_6Na$: 381.1309, found: 381.1326.

Benzyl 3-*O*-benzoyl-*β*-L-rhamnopyranoside (28).

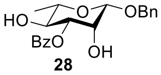

28

Prepared by the same route as general benzoylation in toluene, yielding **28** as a colorless syrup (1.2 g, 85%). ^{1}H NMR ($CDCl_3$, 600 MHz) δ 8.12-8.11 (m, 2 H), 7.59 (m, 1 H), 7.47-7.34 (m, 7 H), 4.96 (m, 2 H), 4.69 (d, 1 H, *J* = 11.9 Hz), 4.63 (s, 1 H), 4.25 (s, 1 H), 3.91 (m, 1 H), 3.42 (m, 1 H), 2.52-2.48 (br, 2 H), 1.46 (d, 3 H, *J* = 6.1 Hz). ^{13}C NMR ($CDCl_3$, 150 MHz) δ 166.8, 136.7, 133.5, 130.0, 129.5, 128.6, 128.5, 128.3, 128.2, 97.7, 72.3, 70.6,

70.5, 69.6, 17.7. HRMS (ESI-TOF, MNa^+) calcd for $C_{20}H_{22}O_6Na$: 381.1309, found: 381.1304.

Benzyl 3-*O*-benzoyl-2,4-di-azide-*β*-L-fucopyranoside (29).

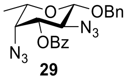

29

^1H NMR ($CDCl_3$, 600 MHz) δ 8.17-8.16 (m, 2 H), 7.65 (m, 1 H), 7.53 (m, 2 H), 7.44-7.34 (m, 5 H), 5.14 (dd, 1 H, *J* = 3.6 Hz, *J* = 10.6 Hz), 5.00 (d, 1 H, *J* = 11.8 Hz), 4.73 (d, 1 H, *J* = 11.9 Hz), 4.45 (d, 1 H, *J* = 7.9 Hz), 3.99-3.95 (m, 2 H), 3.77 (m, 1 H), 1.43 (d, 3 H, *J* = 6.3 Hz). ^{13}C NMR ($CDCl_3$, 150 MHz) δ 165.6, 136.6, 133.9, 130.1, 128.7, 128.6, 128.5, 128.1, 100.6, 73.8, 70.9, 69.4, 63.2, 61.1, 17.4. HRMS (ESI-TOF, MNa^+) calcd for $C_{20}H_{20}N_6O_4Na$: 431.1438, found: 431.1443.

Benzyl 2,4-di-azide-*α*-L-rhamnopyranoside (30).

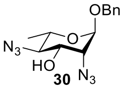

30

The compound **6** (500 mg) was directly dissolved in MeOH (10 mL), and the NaOMe (30 mg) was added. The reaction was allowed to stir at room temperature for 6 h. After the benzoyl group deprotected completely, the reaction was neutralized by IR120, filtered and concentrated in vacuo. Purification of the residue by flash column chromatography afforded 350 mg of the compound **30** in 95% yield. ^1H NMR ($CDCl_3$, 600 MHz) δ 7.41-7.33 (m, 5 H), 4.91 (d, 1 H, *J* = 0.8 Hz), 4.70 (d, 1 H, *J* = 11.8 Hz), 4.52 (d, 1 H, *J* = 11.8 Hz), 4.09 (dd, 1 H, *J* = 3.9 Hz, *J* = 9.8 Hz), 3.94 (dd, 1 H, *J* = 1.3 Hz, *J* = 3.8 Hz), 3.63 (m, 1 H), 3.35 (t, 1 H, *J* = 9.8 Hz, *J* = 9.8 Hz), 1.37 (d, 3 H, *J* = 6.2 Hz). ^{13}C NMR ($CDCl_3$, 150 MHz) δ 136.6, 128.6, 128.2, 128.0, 97.1, 70.3, 69.5, 67.4, 66.2, 63.3, 18.3. HRMS (ESI-TOF, MNa^+) calcd for $C_{13}H_{16}N_6O_3Na$: 327.1176, found: 327.1298.

Benzyl 2,4-di-azide-2,4,6-deoxy-*α*-L-altropyranoside (31).

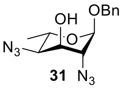

31

Prepared by the same route as general inversion of triflate derivatives, yielding **31** as a

colorless syrup (100 mg, 25%).

^{1}H NMR ($CDCl_3$, 600 MHz) δ 7.43-7.36 (m, 5 H), 4.90 (s, 1 H), 4.79 (d, 1 H, *J* = 11.8 Hz), 4.61 (d, 1 H, *J* = 11.8 Hz), 4.11-4.06 (m, 2 H), 3.86 (dd, 1 H, *J* = 1.8 Hz, *J* = 4.0 Hz), 3.50 (br, 1 H), 3.26 (dd, 1 H, *J* = 3.1 Hz, *J* = 9.7 Hz), 1.39 (d, 3 H, *J* = 6.4 Hz). ^{13}C NMR ($CDCl_3$, 150 MHz) δ 135.9, 128.8, 128.5, 128.3, 96.9, 70.3, 69.4, 63.2, 61.4, 60.9, 18.1. HRMS (ESI-TOF, MH^+) calcd for $C_{13}H_{16}$ N_6O_3H: 305.1357, found: 305.1352.

Benzyl 2,4-di-azide-*β*-L-rhamnopyranoside (36).

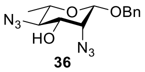

36

The compound **13** (139 mg) was directly dissolved in MeOH (2 mL), and the NaOMe (5 mg) was added. The reaction was allowed to stir at room temperature for 6 h. After the benzoyl group deprotected completely, the reaction was neutralized by IR120, filtered and concentrated in vacuo. Purification of the residue by flash column chromatography afforded 99 mg of the compound **36** in 95% yield.^{1}H NMR ($CDCl_3$, 600 MHz) δ 7.41-7.33 (m, 5 H), 5.01 (d, 1 H, *J* = 12.1 Hz), 4.65 (d, 1 H, *J* = 12.1 Hz), 4.58 (d, 1 H, *J* = 1.2 Hz), 3.99 (dd, 1 H, *J* = 0.8 Hz, *J* = 4.0 Hz), 3.61 (s, 1 H), 3.21-3.14 (m, 2 H), 2.49 (br, 1 H), 1.44 (d, 3 H, *J* = 5.7 Hz). ^{13}C NMR ($CDCl_3$, 150 MHz) δ 136.6, 128.6, 128.1, 127.8, 98.3, 72.2, 71.1, 70.6, 66.0, 64.1, 18.3. HRMS (ESI-TOF, MH^+) calcd for $C_{13}H_{16}N_6O_3H$: 305.1357, found: 305.1244.

Benzyl 2,4-di-azide-2,4,6-deoxy-*β*-L-altropyranoside (37).

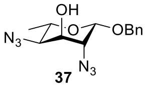

37

Prepared by the same route as general inversion of triflate derivatives, yielding **37** as a colorless syrup (79 mg, 80%). ^{1}H NMR ($CDCl_3$, 600 MHz) δ 7.39-7.32 (m, 5 H), 5.03 (d, 1 H, *J* = 1.4 Hz), 5.00 (d, 1 H, *J* = 12.0 Hz), 4.64 (d, 1 H, *J* = 11.9 Hz), 4.12 (t, 1 H, *J* = 3.5 Hz, *J* = 3.4 Hz), 3.88 (m, 1 H), 3.78 (dd, 1 H, *J* = 1.4 Hz, *J* = 4.0 Hz), 3.39 (dd, 1 H, *J* = 2.9 Hz, *J* = 9.5 Hz), 1.44 (d, 3 H, *J* = 6.3 Hz). ^{13}C NMR ($CDCl_3$, 150 MHz) δ 137.0, 128.5, 127.9, 127.6, 97.1, 70.8,69.5, 69.0, 63.3, 62.1, 18.6. HRMS (ESI-TOF, MNa^+) calcd for $C_{13}H_{16}$ N_6O_3H: 305.1357, found: 305.1437.

Benzyl 2,4-di-azide-2,3,4-tri-deoxy-2-dehydro-*α*-L-rhamnopyranoside (39).

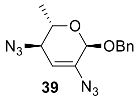

^{1}H NMR ($CDCl_3$, 600 MHz) δ 7.42-7.34 (m, 5 H), 5.49 (d, 1 H, *J*=2.1 Hz), 4.97 (s, 1 H), 4.81 (d, 1 H, *J* = 11.6 Hz), 4.68 (d, 1 H, *J* = 11.6 Hz), 3.90 (m, 1 H), 3.72 (dd, 1 H, *J* = 0.7 Hz, *J* = 9.3 Hz), 1.33 (d, 3 H, *J* = 6.2 Hz). ^{13}C NMR ($CDCl_3$, 150 MHz) δ 138.1, 136.9, 128.5, 128.4, 128.1, 111.0, 93.1, 70.5, 66.5, 60.5. HRMS (ESI-TOF, MNa^+) calcd for $C_{13}H_{14}N_6O_2H$: 289.1301, found: 289.1289.

2,4-di-acetamido-2,4,6-tri-deoxy-L-altropyranoside (40).

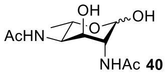

Prepared by the same route as general deprotection of the di-azido derivatives, yielding **40** as a colorless foam (59 mg, 74%).

^{1}H NMR (D_2O, 600MHz) δ *α* anomer: 4.95 (d, 1 H, *J* = 2.3 Hz), 4.14 (m, 1 H), 3.95 (dd, 1 H, *J* = 3.7 Hz, *J* = 4.5 Hz), 3.86-3.83 (m, 2 H), 1.95 (s, 3 H), 1.94 (s, 3 H), 1.14 (d, 3 H, *J* = 6.5 Hz);

β anomer: 5.17 (d, 1 H, *J* = 1.7 Hz), 4.00 (dd, 1 H, *J* = 2.0 Hz, *J* = 3.2 Hz), 3.85-3.80 (m, 2 H), 3.72 (dd, 1 H, *J* = 3.0 Hz, *J* = 10.4 Hz), 1.98 (s, 3 H), 1.93 (s, 3 H), 1.11 (d, 3 H, *J* = 6.2 Hz);

^{13}C NMR (D_2O, 150 MHz) δ 174.7, 174.0, 174.0, 92.2, 90.5, 69.4, 68.0, 67.2, 64.2, 53.5, 52.3, 50.9, 50.6, 48.8, 21.9, 21.9, 21.9, 17.2, 16.7.

HRMS (ESI-TOF, MH^+) calcd for $C_{10}H_{18}N_2O_5H$: 247.1288, found: 247.1287.

2,4-di-acetamido-2,4,6-tri-deoxy-L-rhamnopyranoside (41).

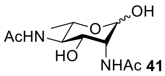

Prepared by the same route as general deprotection of the di-azido derivatives, yielding **41** as a colorless foam (24 mg, 60%).

^{1}H NMR (D_2O, 600 MHz) δ *α* anomer: 4.87 (d, 1 H, *J* = 1.6 Hz), 4.38 (dd, 1 H, *J* = 1.3 Hz,

J = 4.3 Hz), 3.74 (dd, 1 H, J = 4.4 Hz, J = 10.8 Hz), 3.58 (t, 1 H, J = 10.3 Hz, J = 10.5 Hz), 3.41 (m, 1 H), 2.02 (s, 3 H), 1.94 (s, 3 H), 1.12 (d, 3 H, J=6.2 Hz);

β anomer: 5.02 (d, 1 H, J = 1.0 Hz), 4.21 (dd, 1 H, J = 1.1 Hz, J = 4.4 Hz), 3.97 (dd, 1 H, J = 4.6 Hz, J = 10.8 Hz), 3.88 (m, 1 H), 3.68 (t, 1 H, J = 10.5 Hz, J = 10.5 Hz), 1.98 (s, 3 H), 1.94 (s, 3 H), 1.10 (d, 3 H, J = 6.3 Hz);

^{13}C NMR (D_2O, 150 MHz) δ 175.8, 174.9, 174.7, 174.7, 92.8, 92.7, 71.6, 69.9, 67.0, 66.5, 53.5, 53.5, 53.2, 52.7, 48.8, 22.1, 22.1, 22.1, 21.9, 16.8, 16.7.

HRMS (ESI-TOF, MH^+) calcd for $C_{10}H_{18}N_2O_5H$: 247.1288, found: 247.1288.

3,4- di-acetamido-6-deoxyl-L-allopyranoside (42).

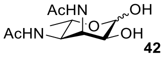

42

Prepared by the same route as general deprotection of the di-azido derivatives, yielding **42** as a colorless foam (16 mg, 40%).

^{1}H NMR (D_2O, 600 MHz) δ α anomer: 4.97 (s, 1 H), 4.89 (dd, 1 H, J = 4.4 Hz, J = 8.6 Hz), 4.17 (dd, 1 H, J = 2.3 Hz, J = 6.7 Hz), 4.12 (d, 1 H, J = 4.3 Hz), 3.80 (dd, 1 H, J = 2.1 Hz, J = 8.6 Hz), 2.16 (s, 3 H), 1.94 (s, 3 H), 1.02 (d, 3 H, J = 6.7 Hz);

β anomer: 5.18 (s, 1 H), 4.60 (dd, 1 H, J = 4.7 Hz, J = 7.4 Hz), 4.17 (m, 1H), 4.03 (d, 1 H, J = 4.6 Hz), 3.79 (m, 1 H), 2.13 (s, 3 H), 2.11 (s, 3 H), 1.08 (d, 3 H, J = 6.6 Hz);

^{13}C NMR (D_2O, 150 MHz) δ 175.9, 175.1, 173.8, 93.9, 91.8, 71.3, 71.0, 66.1, 65.5, 64.9, 64.3, 55.7, 55.0, 50.8, 50.2, 30.2, 21.8, 21.3, 21.1, 17.5, 17.4.

HRMS (ESI-TOF, MH^+) calcd for $C_{10}H_{18}N_2O_5H$: 247.1288, found: 247.1286.

2,4-di-acetamido-D-lyxopyranoside (43).

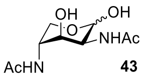

43

Prepared by the same route as general deprotection of the di-azido derivatives, yielding **43** as a colorless foam (30 mg, 76%).

^{1}H NMR (D_2O, 600 MHz) δ α anomer: 4.86 (d, 1 H, J = 2.0 Hz), 4.30 (dd, 1 H, J = 1.9 Hz, J = 3.6 Hz), 3.94 (m, 1 H), 3.87-3.77 (m, 2 H), 3.19 (dd, 1 H, J = 8.9 Hz, J = 11.9 Hz), 2.00 (s, 3 H), 1.92 (s, 3 H);

β anomer: 4.91 (d, 1 H, J = 5.3 Hz), 4.00 (t, 1 H, J = 4.1 Hz, J = 4.9 Hz), 3.94 (dd, 1 H, J

= 3.5 Hz, *J* = 7.0 Hz), 3.87-3.77 (m, 2 H), 3.64 (dd, 1 H, *J* = 6.1 Hz, *J* = 12.0 Hz), 1.96 (s, 3 H), 1.93 (s, 3 H);

^{13}C NMR (D_2O, 150 MHz) δ 175.4, 174.6, 174.4, 174.3, 93.1, 93.0, 70.0, 69.0, 67.2, 62.0, 61.8, 61.6, 58.3, 52.1, 50.3, 50.3, 49.0, 48.8, 48.1, 22.0, 21.9, 21.9, 21.9.

HRMS (ESI-TOF, MH^+) calcd for $C_9H_{16}N_2O_5H$: 233.1132, found: 233.1128.

2,4- di-acetamido-L-fucopyranoside (44).

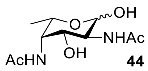

Prepared by the same route as general deprotection of the di-azido derivatives, yielding **44** as a colorless foam (22 mg, 55%).

^{1}H NMR (D_2O, 600 MHz) δ *α* anomer: 5.11 (d, 1 H, *J* = 3.6 Hz), 4.28 (dd, 1 H, *J* = 6.2 Hz, *J* = 12.7 Hz), 4.18 (d, 1 H, *J* = 3.1 Hz), 3.98 (dd, 1 H, *J* = 4.3 Hz, *J* = 11.3 Hz), 3.90 (dd, 1 H, *J* = 3.6 Hz, *J* = 11.3 Hz), 1.94 (s, 6 H), 1.01 (d, 3 H, *J* = 6.5 Hz);

β anomer: 4.53 (d, 1 H, *J* = 8.5 Hz), 4.14 (d, 1 H, *J* = 3.9 Hz), 3.81 (dd, 1 H, *J* = 6.2 Hz, *J* = 12.5 Hz), 3.77 (dd, 1 H, *J* = 4.4 Hz, *J* = 11.0 Hz), 3.66 (t, 1 H, *J* = 8.6 Hz, *J* = 10.8 Hz), 2.02 (s, 3 H), 2.01 (s, 3 H), 1.06 (d, 3 H, *J* = 6.4 Hz);

^{13}C NMR (D_2O, 150 MHz) δ 175.6, 175.5, 174.9, 174.6, 95.4, 90.9, 70.1, 69.9, 66.5, 65.0, 53.9, 53.6, 52.9, 50.6, 48.8, 22.1, 21.9, 21.8, 15.7, 15.7.

HRMS (ESI-TOF, MH^+) calcd for $C_{10}H_{18}N_2O_5H$: 247.1288, found: 247.1289.

Pseudaminic Acid (Pse5Ac7Ac) (45).

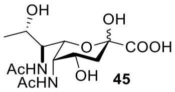

Prepared by the same route as general enzymatic reaction based on NeuB3, yielding **45** as a colorless foam (11 mg, 80%).

^{1}H NMR (D_2O, 600 MHz) δ 4.13 (d, 1 H, *J* = 3.6 Hz), 4.09-4.02 (m, 2 H), 3.97-3.95 (m, 2 H), 1.90 (dd, 1 H, *J* = 4.8 Hz, *J* = 12.5 Hz), 1.86 (s, 3 H), 1.821 (s, 3 H), 1.66 (t, 1 H, *J* = 12.7 Hz, *J* = 12.9 Hz), 0.96 (d, 3 H, *J* = 6.5 Hz); ^{13}C NMR (D_2O, 150 MHz) δ 174.6, 173.7, 172.9, 171.9, 165.6, 96.1, 95.3, 72.8, 70.0, 66.9, 66.6, 65.8, 64.6, 53.1, 52.7, 52.7, 48.6, 48.5, 47.8, 35.2, 34.2, 22.0, 22.0, 22.0, 21.9, 15.3, 15.2, 12.9. HRMS (ESI-TOF, MH^+)

calcd for $C_{13}H_{22}N_2O_8H$: 335.1449, found: 335.1452.

Pseudaminic Acid (Pse5Ac7Ac) isomer (46).

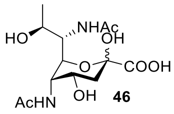

46

Prepared by the same route as general enzymatic reaction based on NeuB3, yielding **46** as a colorless foam (8.3 mg, 61%).

^{1}H NMR (D_2O, 600 MHz) δ 4.52 (d, 1 H, *J* = 10.8 Hz), 4.01 (dd, 1 H, *J* = 2.9 Hz, *J* = 5.9 Hz), 3.81-3.78 (m, 3 H), 2.07 (dd, 1 H, *J* = 3.0 Hz, *J* = 14.8 Hz), 2.02 (dd, 1 H, *J* = 3.4 Hz, *J* = 12.5 Hz), 1.89 (s, 3 H), 1.88 (s, 3 H), 1.07 (d, 3 H, *J* = 5.6 Hz); ^{13}C NMR (D_2O, 150 MHz) δ 174.0,173.7, 173.4, 165.7, 95.3, 68.9, 66.8, 65.9, 65.8, 65.7, 65.1, 53.8, 53.5, 48.6, 48.5, 38.8, 36.4, 21.8, 21.8, 21.6, 19.1, 18.5. HRMS (ESI-TOF, MH^+) calcd for $C_{13}H_{22}N_2O_8H$: 335.1449, found: 335.1451.

Benzyl *β*-L-fucopyranoside (S1)(Brito-Arias et al., 2002).

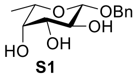

S1

^{1}H NMR ($CDCl_3$, 600 MHz) δ 7.37-7.25 (m, 5 H), 4.86 (d, 1 H, *J* = 11.6 Hz), 4.55 (d, 1 H, *J* = 11.7 Hz), 4.21 (d, 1 H, *J* = 7.7 Hz), 3.69 (t, 1 H, *J* = 9.1 Hz, *J* = 8.4 Hz), 3.53 (d, 1 H, *J* = 2.7 Hz), 3.44 (dd, 1 H, *J* = 3.1 Hz, *J* = 9.7 Hz), 3.33 (m, 1 H), 1.24 (d, 3 H, *J* = 6.4 Hz); ^{13}C NMR ($CDCl_3$, 150 MHz) δ 137.4, 128.4, 128.3, 127.8, 102.1, 73.9, 71.6, 70.9, 70.8, 70.5, 16.3.

Benzyl 2,4-diacetamido-2,4,6-tri-deoxy-*α*-L-altropyranoside (S2).

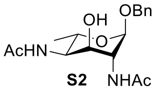

S2

^{1}H NMR ($CDCl_3$, 600 MHz) δ 7.41-7.33 (m, 5 H), 6.10 (d, 1 H, *J* = 9.2 Hz), 5.83 (d, 1 H, *J* = 8.8 Hz), 4.81 (s, 1 H), 4.73 (d, 1 H, *J* = 11.6 Hz), 4.57 (d, 1 H, *J* = 11.7 Hz), 4.42 (dd, 1 H, *J* = 1.0 Hz, *J* = 8.7 Hz), 3.95 (m, 1 H), 3.84 (m, 1 H), 3.75 (s, 1 H), 2.02 (s, 6 H), 1.28 (d, 3 H, *J* = 6.3 Hz). ^{13}C NMR ($CDCl_3$, 150 MHz) δ 170.0, 169.5, 135.9, 128.8, 128.5, 128.2, 98.2, 70.1, 69.1, 64.2, 50.0, 49.8, 23.5, 23.3, 17.8. HRMS (ESI-TOF, MH^+) calcd for $C_{17}H_{24}N_2O_5H$: 337.1758, found: 337.1768.

NMR spectra for new compounds

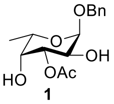

1

^{1}H NMR Spectroscopy of Benzyl 3-*O*-acetyl-α-L-fucopyranoside (1)

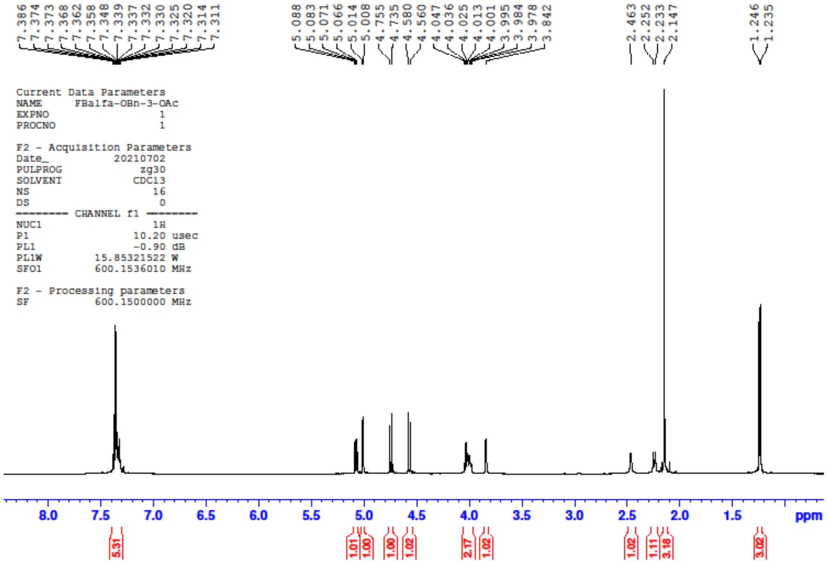

^{13}C NMR Spectroscopy of Benzyl 3-*O*-acetyl-α-L-fucopyranoside (1)

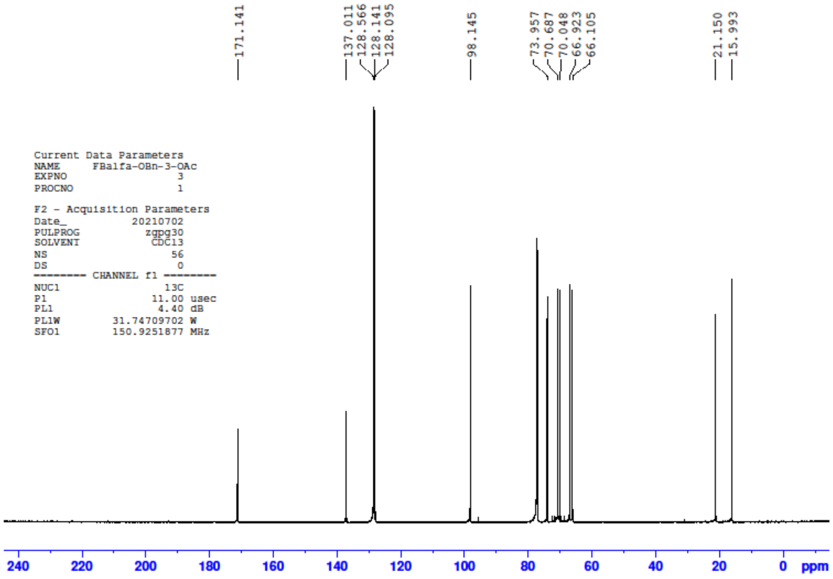

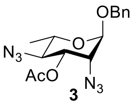

3

^{1}H NMR Spectroscopy of Benzyl 3-*O*-acetyl-2,4-di-azide-α-L-rhamnopyranoside (3)

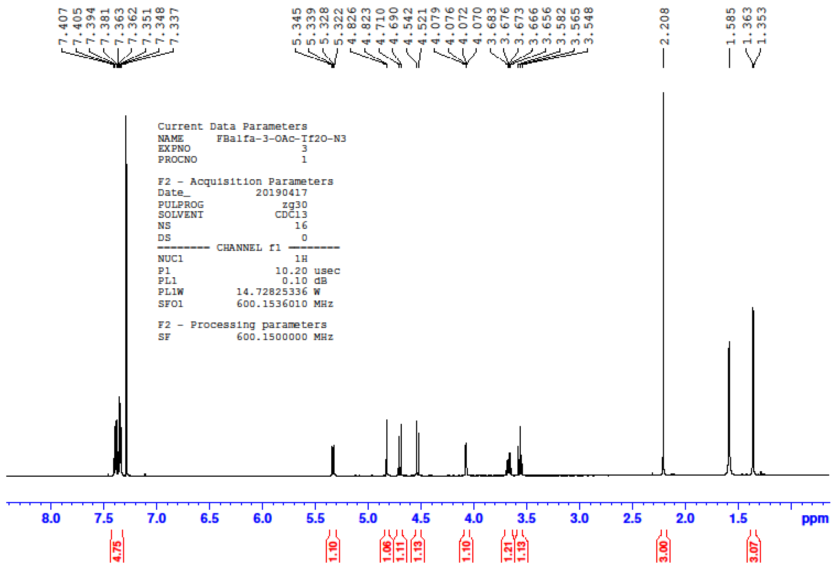

^{13}C NMR Spectroscopy of Benzyl 3-*O*-acetyl-2,4-di-azide-α-L-rhamnopyranoside (3)

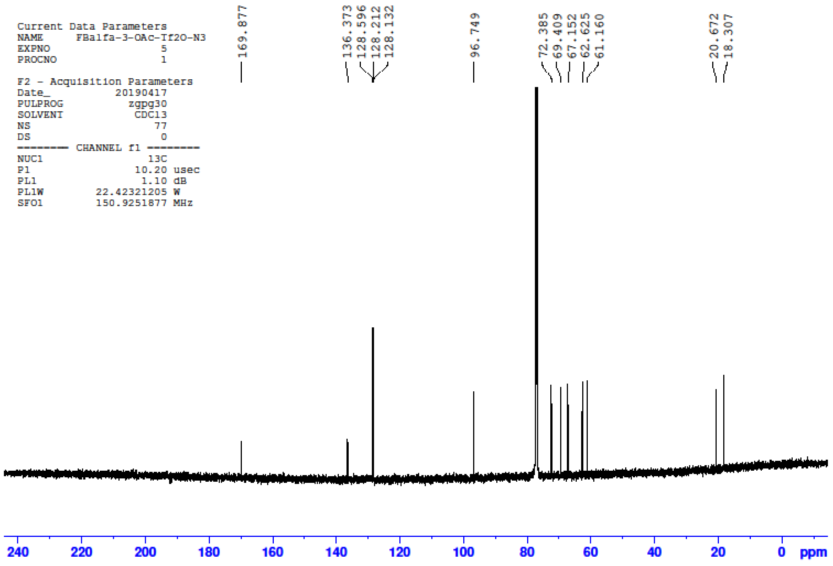

COSY NMR Spectroscopy of Benzyl 3-*O*-acetyl-2,4-di-azide-α-L-rhamnopyranoside (3)

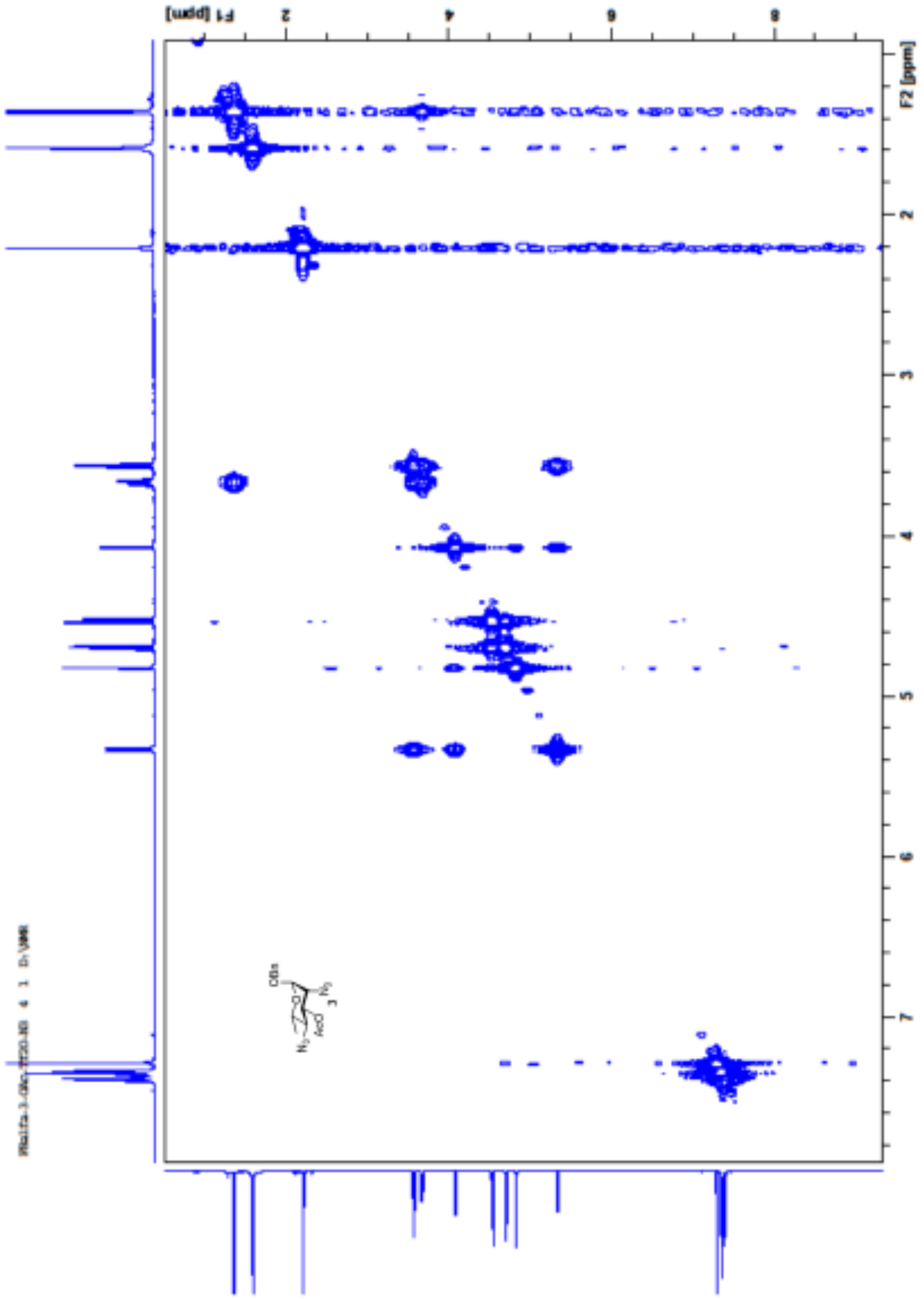

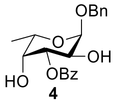

4

^{1}H NMR Spectroscopy of Benzyl 3-*O*-benzoyl-*α*-L-fucopyranoside (4)

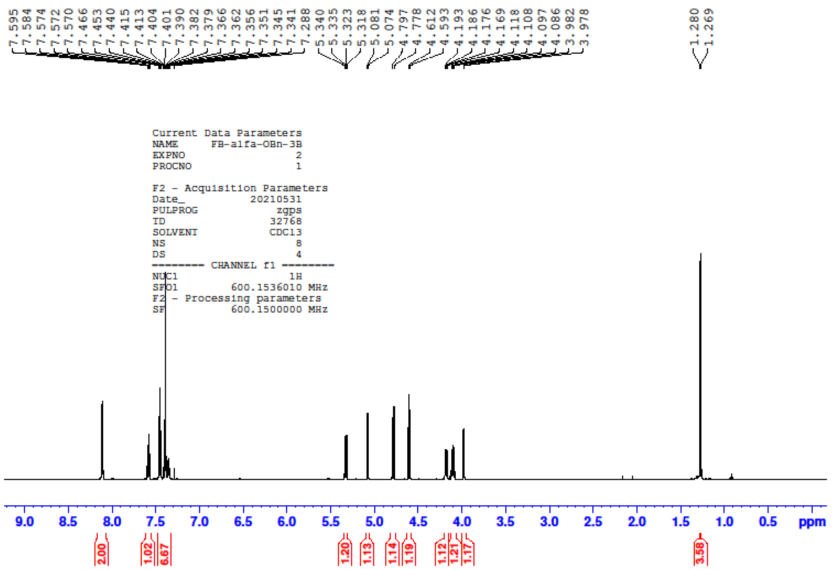

^{13}C NMR Spectroscopy of Benzyl 3-*O*-benzoyl-*α*-L-fucopyranoside (4)

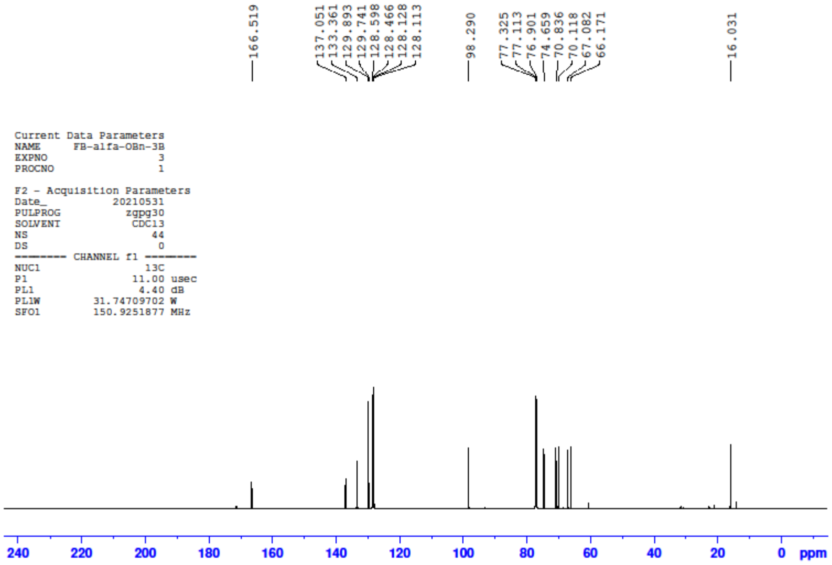

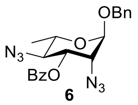

6

^{1}H NMR Spectroscopy of Benzyl 3-*O*-benzoly-2,4-di-azide-*a*-L-rhamnopyranoside (6)

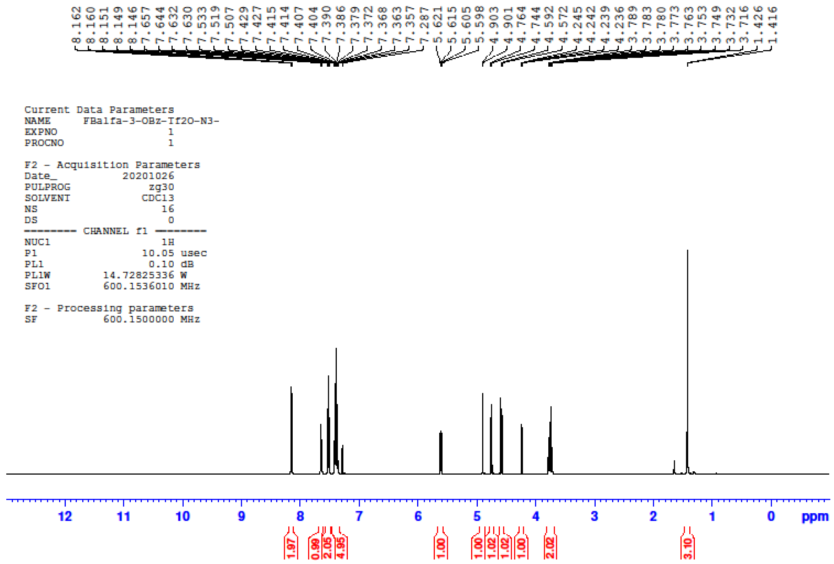

^{13}C NMR Spectroscopy of Benzyl 3-*O*-benzoly-2,4-di-azide-*a*-L-rhamnopyranoside (6)

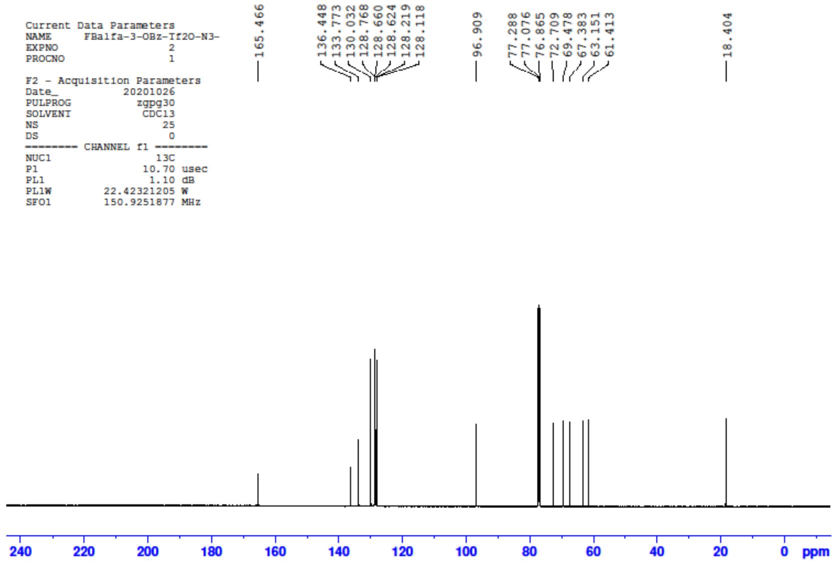

COSY NMR Spectroscopy of Benzyl 3-*O*-benzoly-2,4-di-azide-*a*-L-rhamnopyranoside (6)

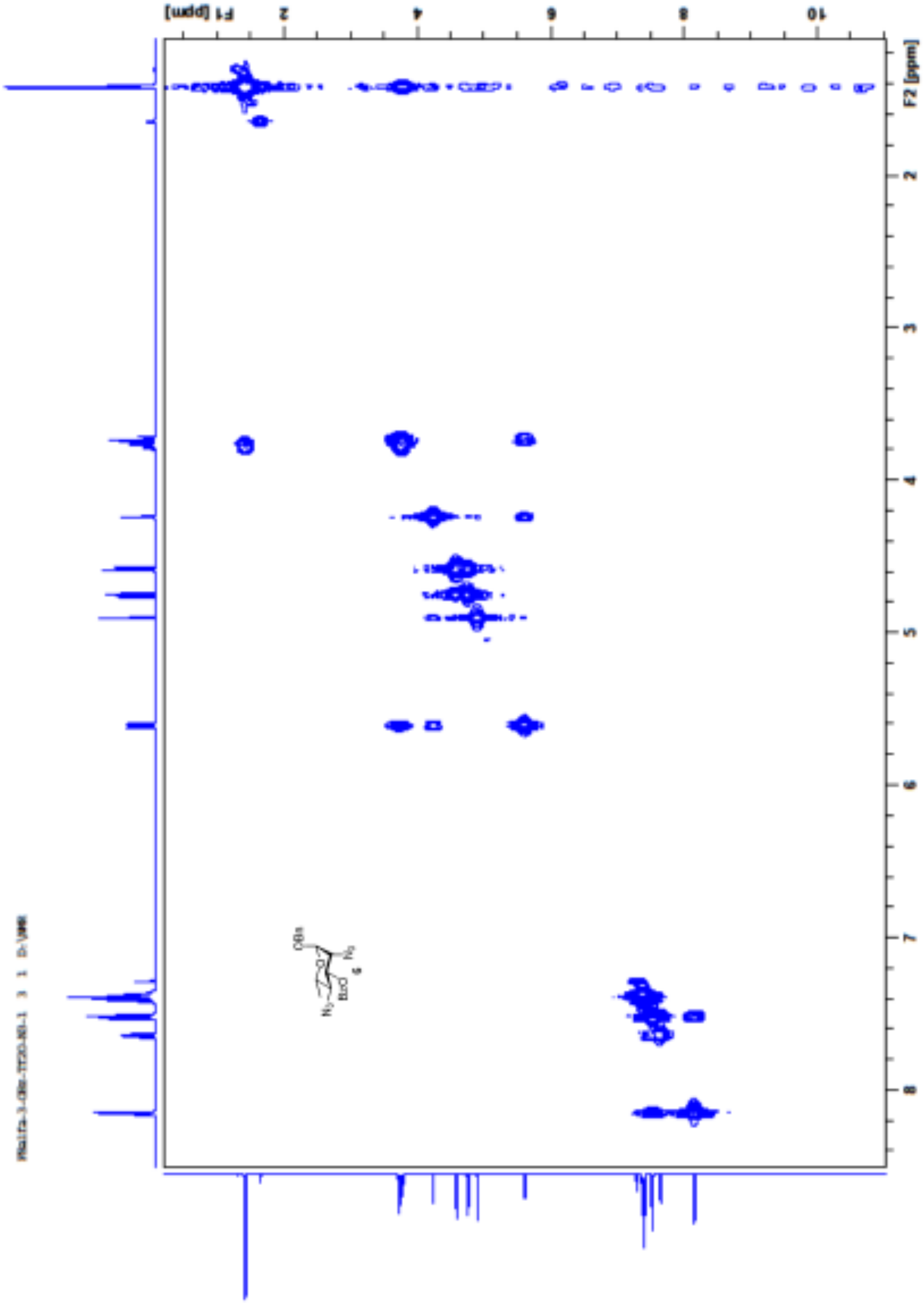

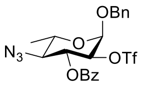

11

^{1}H NMR Spectroscopy of Benzyl 6-deoxy-2-*O*-triflate-3-*O*-benzoly-4-azido-α-L-glucopyranoside (11)

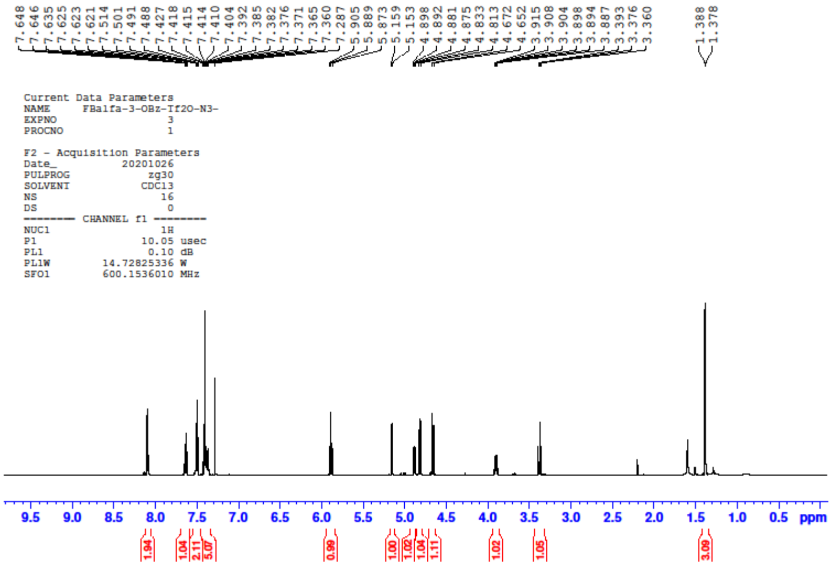

^{13}C NMR Spectroscopy of Benzyl 6-deoxy-2-*O*-triflate-3-*O*-benzoly-4-azido-α-L-glucopyranoside (11)

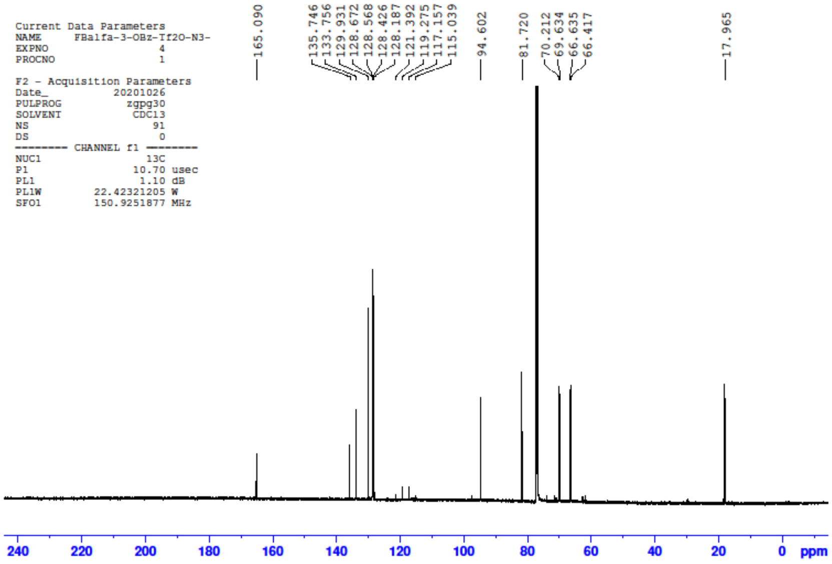

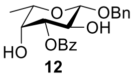

12

^{1}H NMR Spectroscopy of Benzyl 3-*O*-benzoyl-*β*-L-fucopyranoside (12)

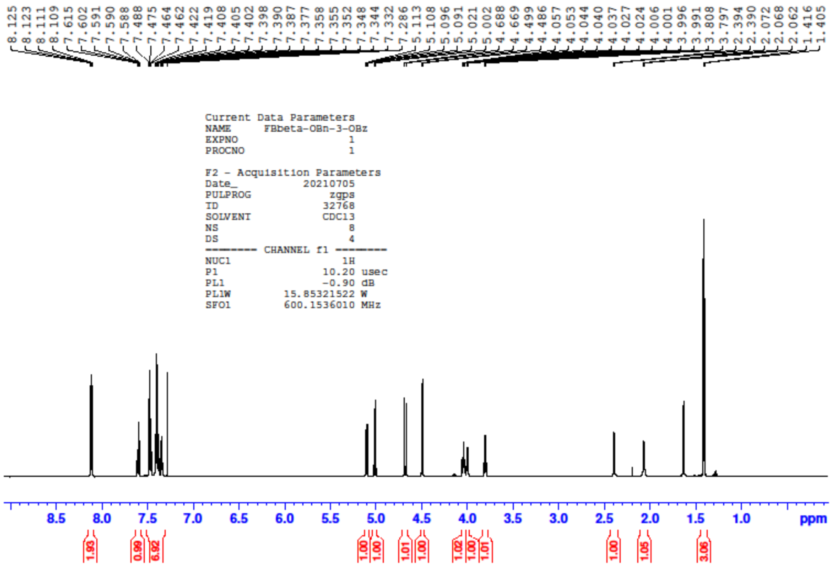

^{13}C NMR Spectroscopy of Benzyl 3-*O*-benzoyl-*β*-L-fucopyranoside (12)

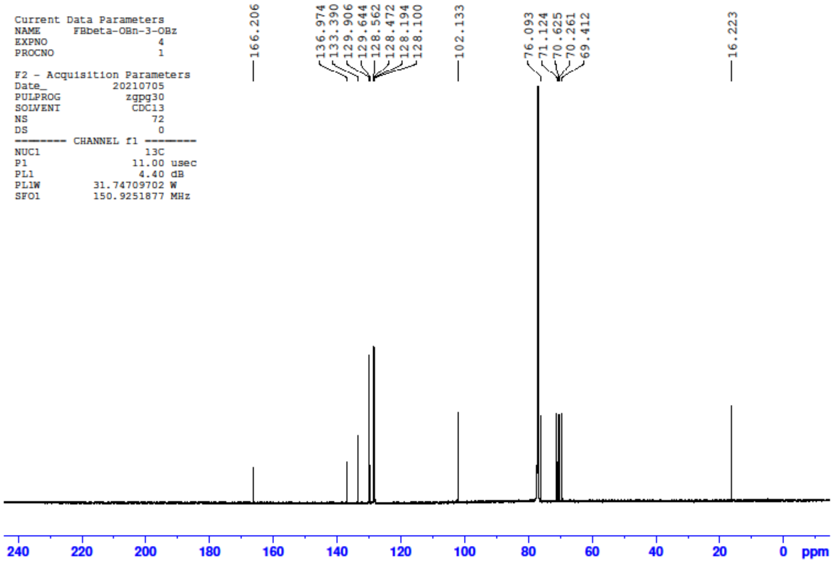

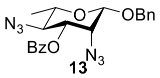

13

^{1}H NMR Spectroscopy of Benzyl 3-*O*-benzoly-2,4-di-azide-β-L-rhamnopyranoside (13)

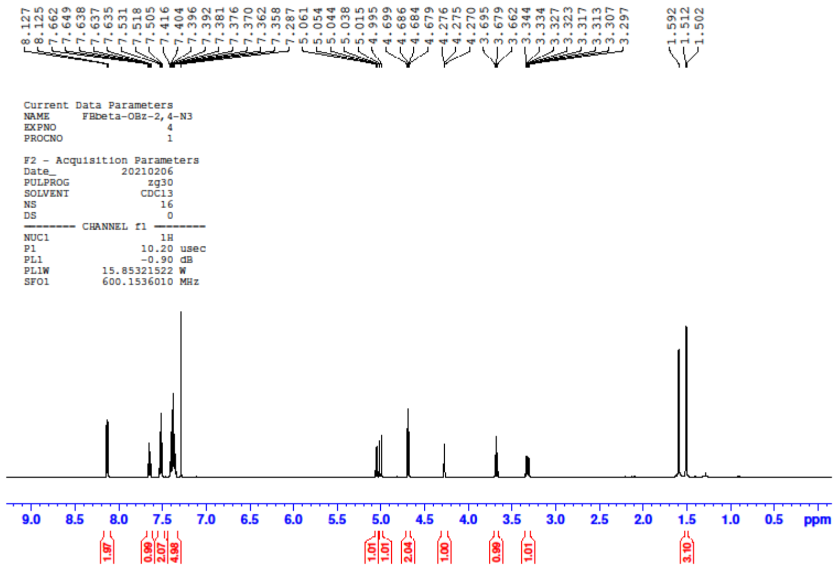

^{13}C NMR Spectroscopy of Benzyl 3-*O*-benzoly-2,4-di-azide-β-L-rhamnopyranoside (13)

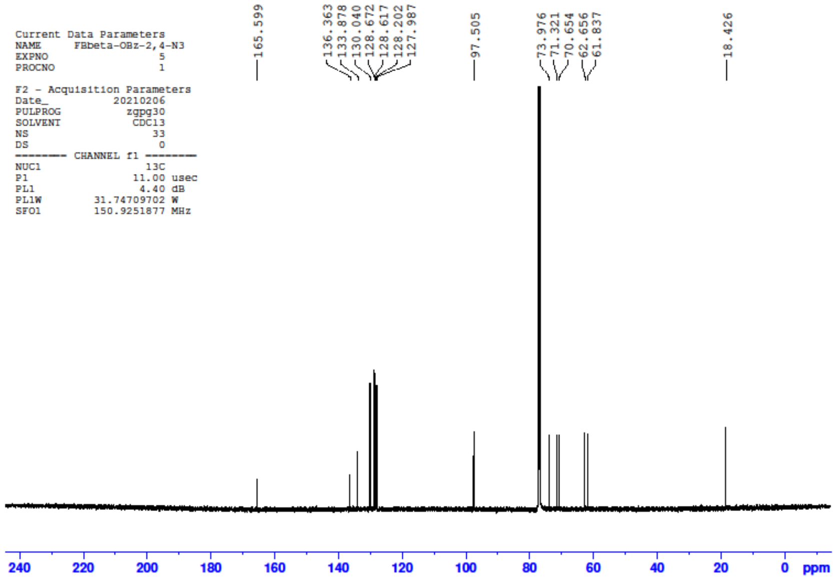

COSY NMR Spectroscopy of Benzyl 3-*O*-benzoly-2,4-di-azide-β-L-rhamnopyranoside (13)

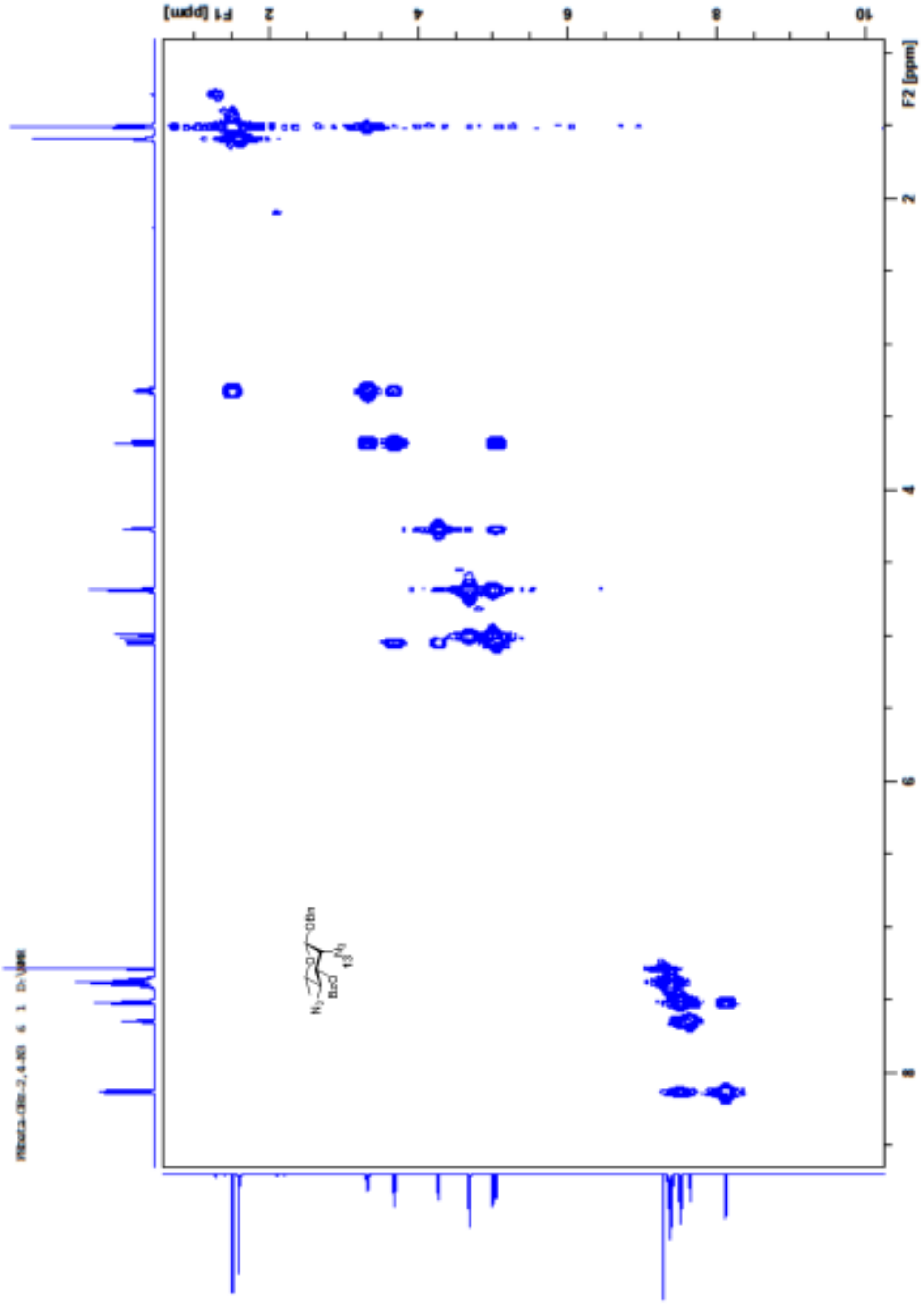

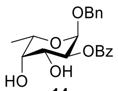

14

^{1}H NMR Spectroscopy of Benzyl 2-*O*-benzoly-*α*-L-fucopyranoside (14)

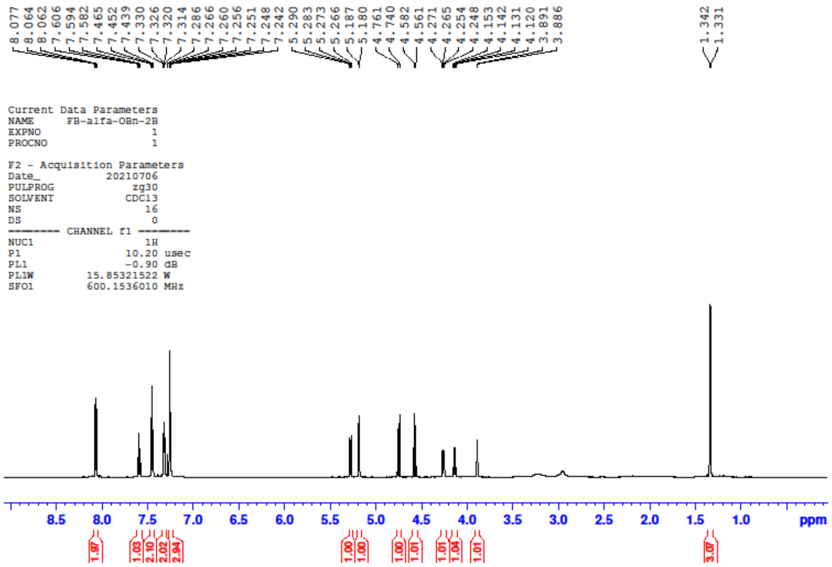

^{13}C NMR Spectroscopy of Benzyl 2-*O*-benzoly-*α*-L-fucopyranoside (14)

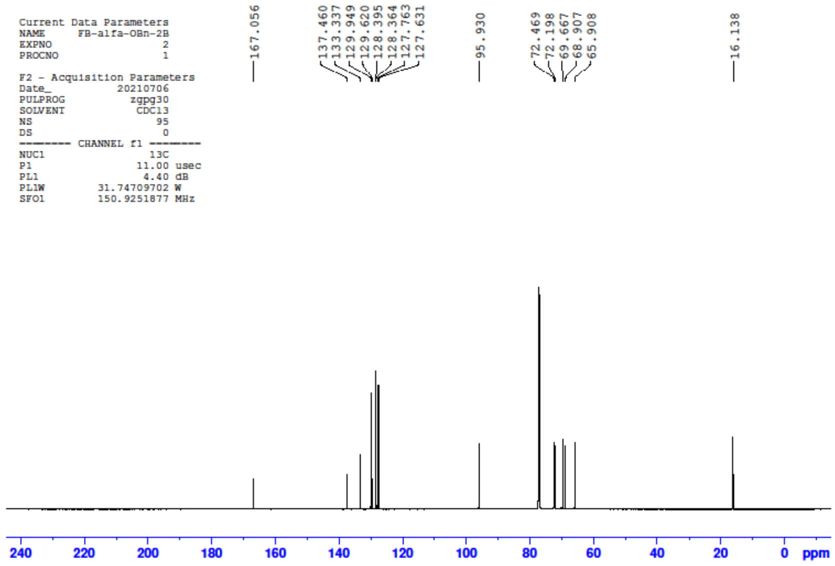

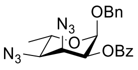

15

^{1}H NMR Spectroscopy of Benzyl 2-*O*-benzoly-3,4-di-azide-6-deoxyl-*α*-L-allopyranoside (15)

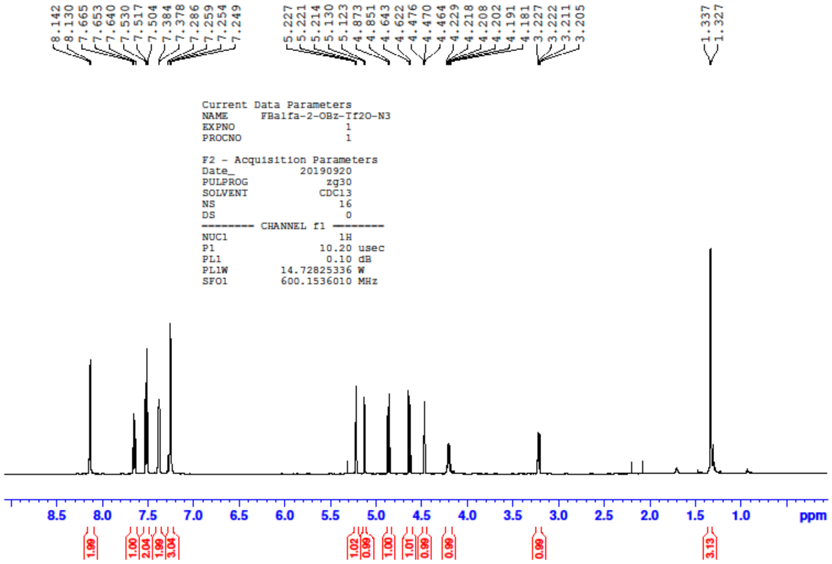

^{13}C NMR Spectroscopy of Benzyl 2-*O*-benzoly-3,4-di-azide-6-deoxyl-*α*-L-allopyranoside (15)

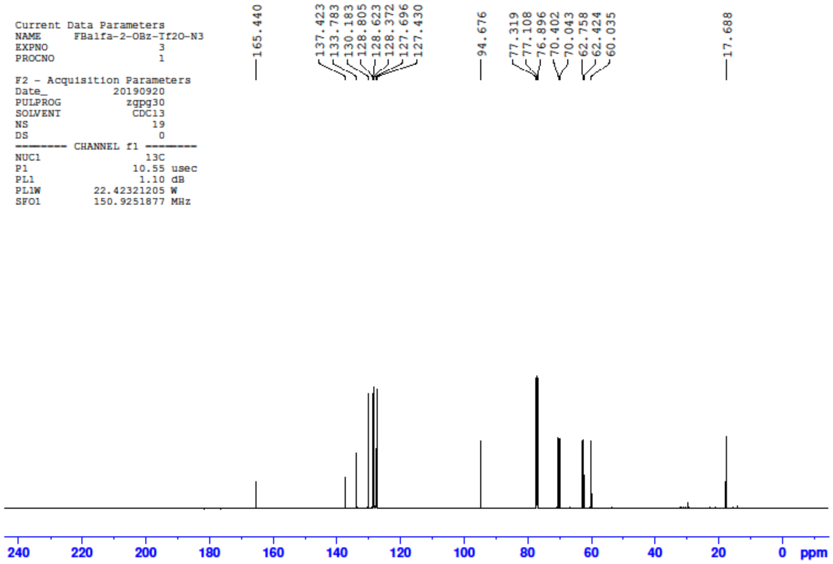

COSY NMR Spectroscopy of Benzyl 2-*O*-benzoly-3,4-di-azide-6-deoxyl-α-L-allopyranoside (15)

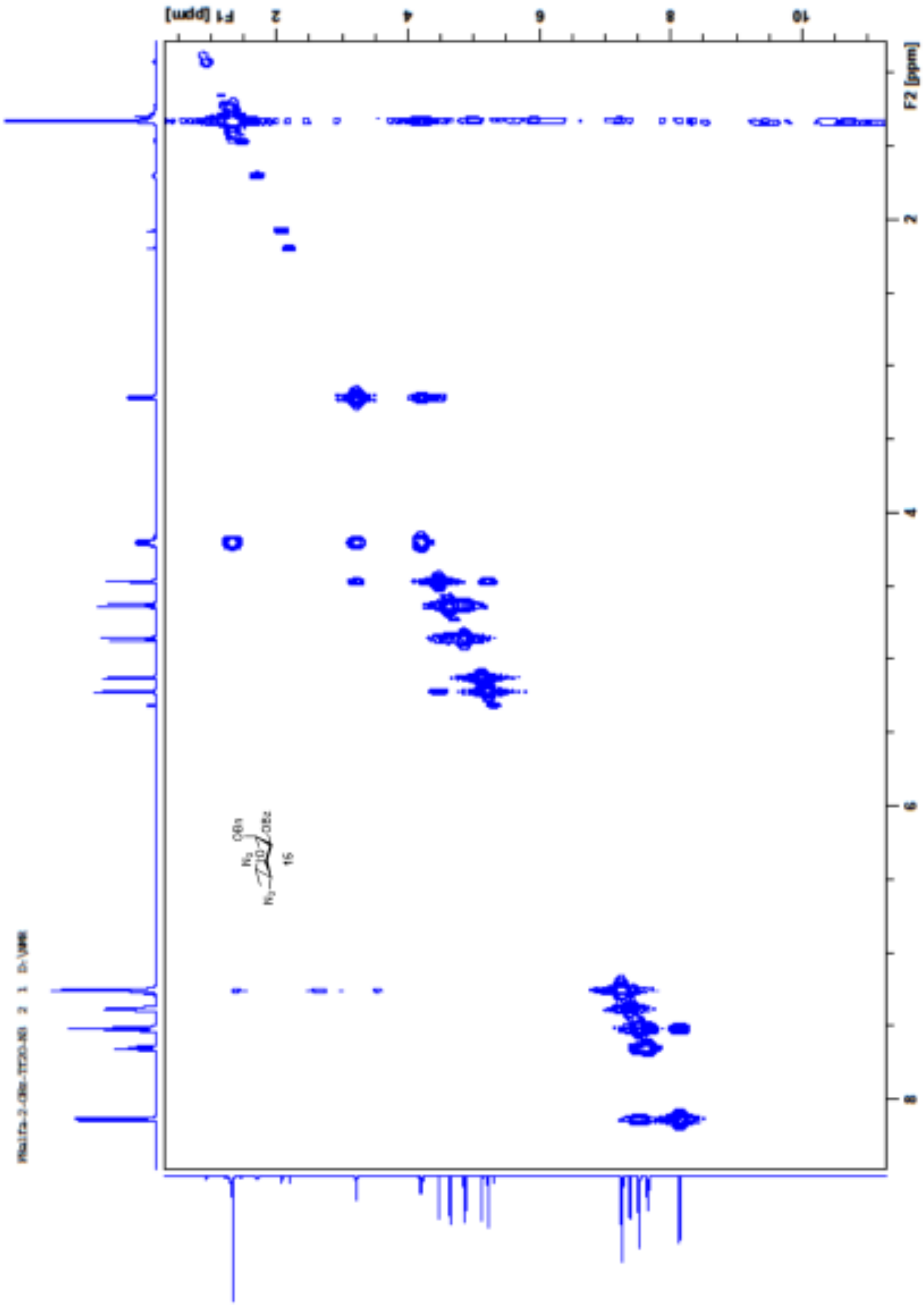

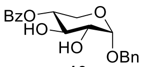

16

^{1}H NMR Spectroscopy of Benzyl 4-*O*-benzoly-α-D-xylopyranoside (16)

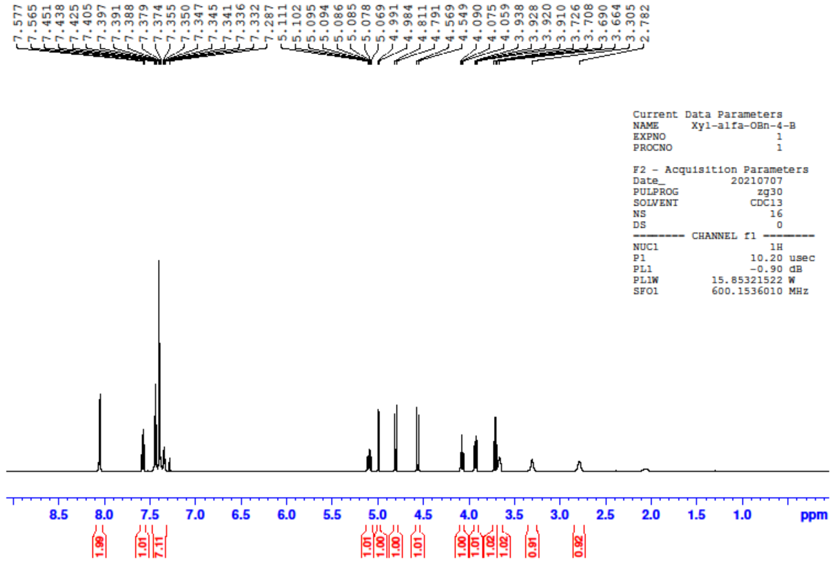

^{13}C NMR Spectroscopy of Benzyl 4-*O*-benzoly-α-D-xylopyranoside (16)

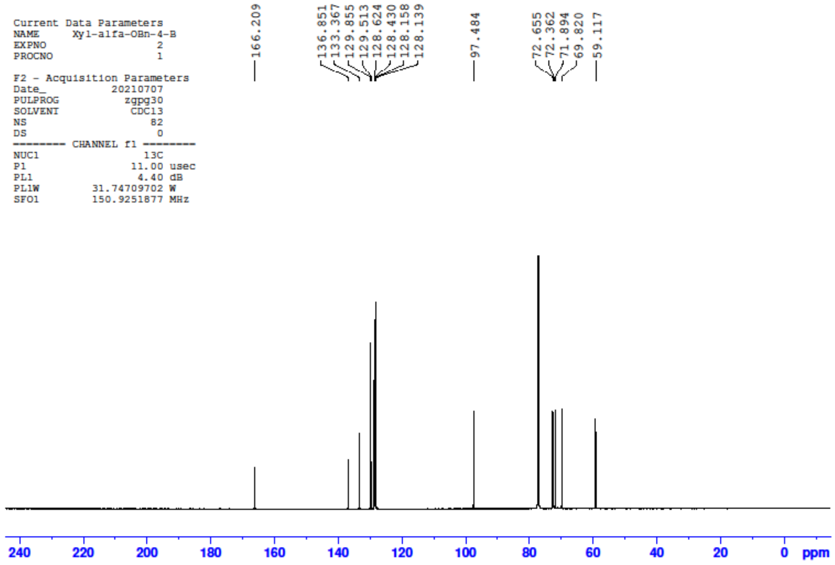

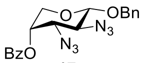

17

^{1}H NMR Spectroscopy of Benzyl 4-*O*-benzoly-2,3-di-azide-α-D-arabinopyranoside (17)

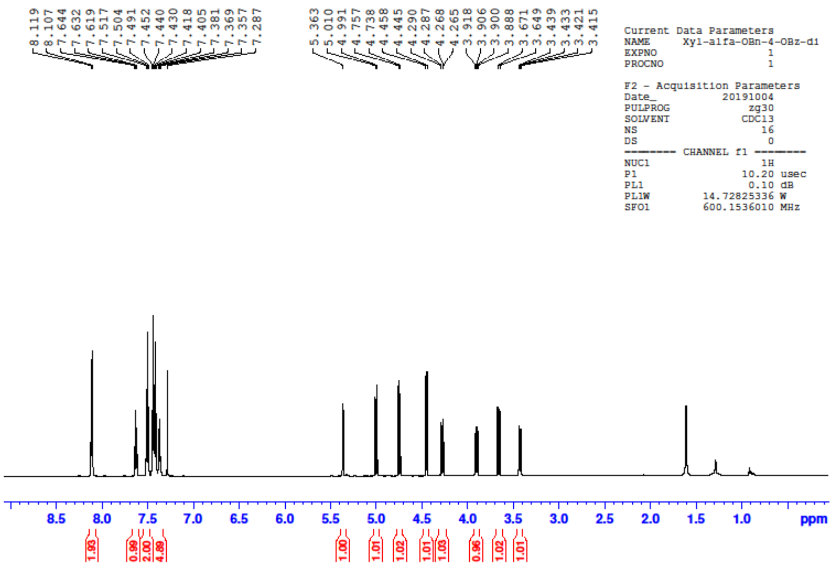

^{13}C NMR Spectroscopy of Benzyl 4-*O*-benzoly-2,3-di-azide-α-D-arabinopyranoside (17)

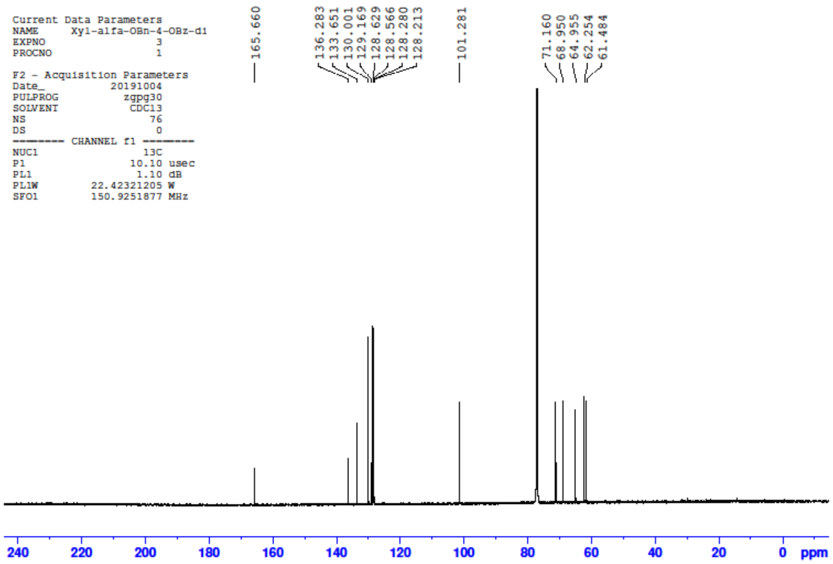

COSY NMR Spectroscopy of Benzyl 4-*O*-benzoly-2,3-di-azide-α-D-arabinopyranoside (17)

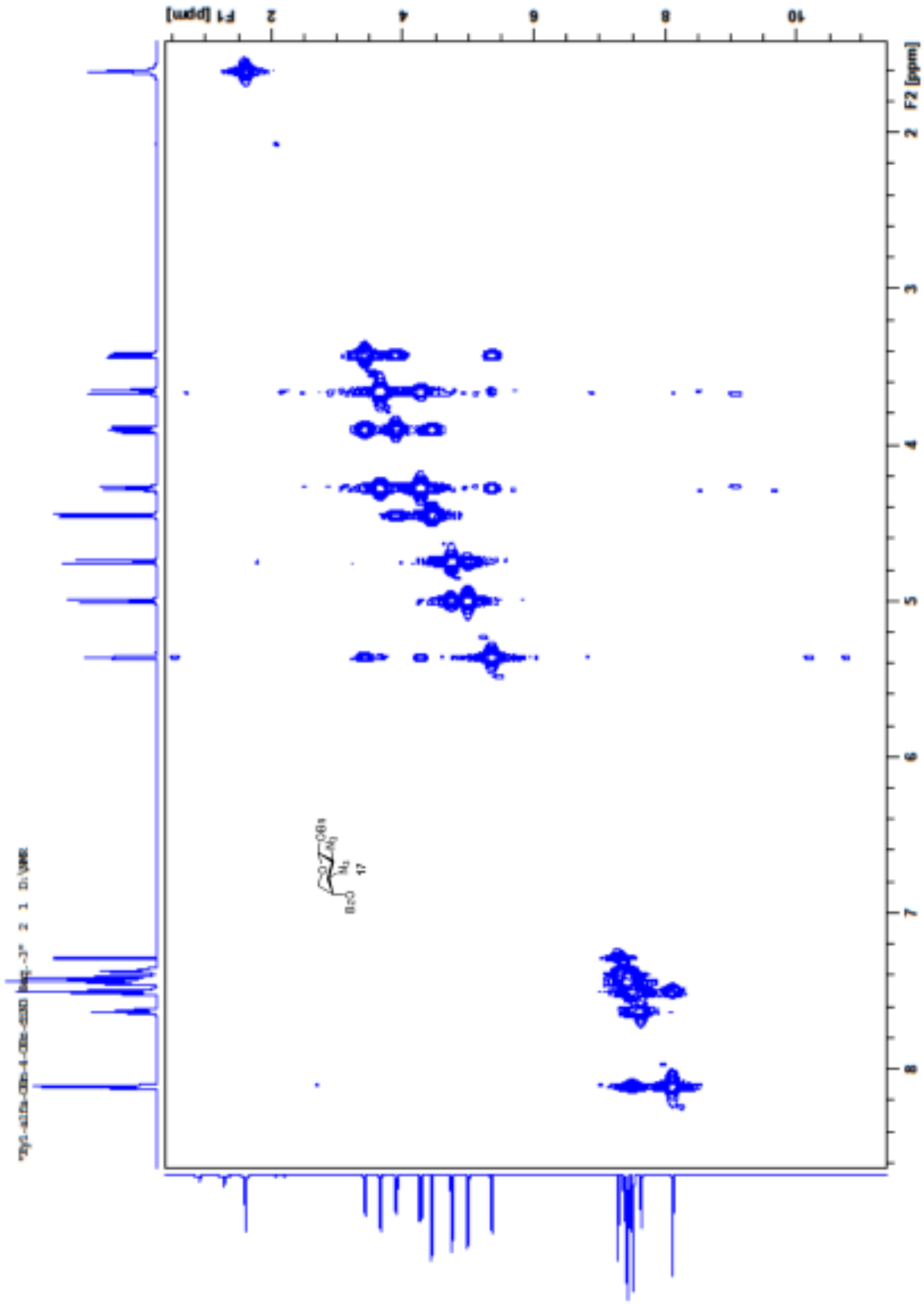

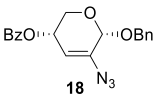

^{1}H NMR Spectroscopy of Benzyl 2-azide-2,3-di-deoxy-3-dehydro-4-*O*-benzoly-α-D-lyxopyranoside (18)

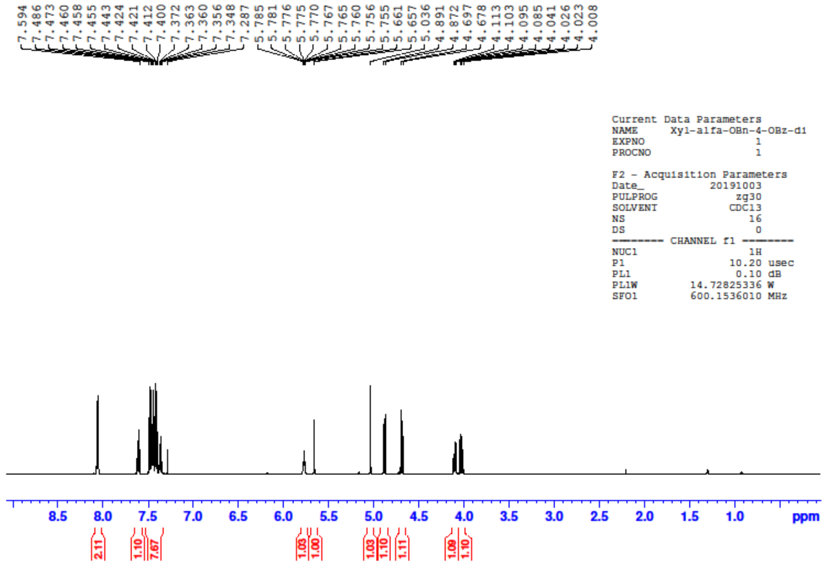

^{13}C NMR Spectroscopy of Benzyl 2-azide-2,3-di-deoxy-3-dehydro-4-*O*-benzoly-α-D-lyxopyranoside (18)

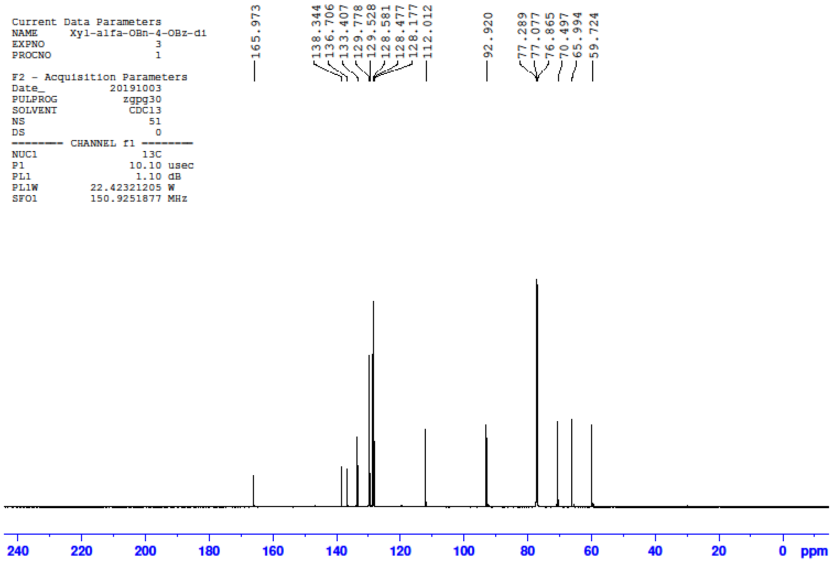

COSY NMR Spectroscopy of Benzyl 2-azide-2,3-di-deoxy-3-dehydro-4-*O*-benzoly-α-D-lyxopyranoside (18)

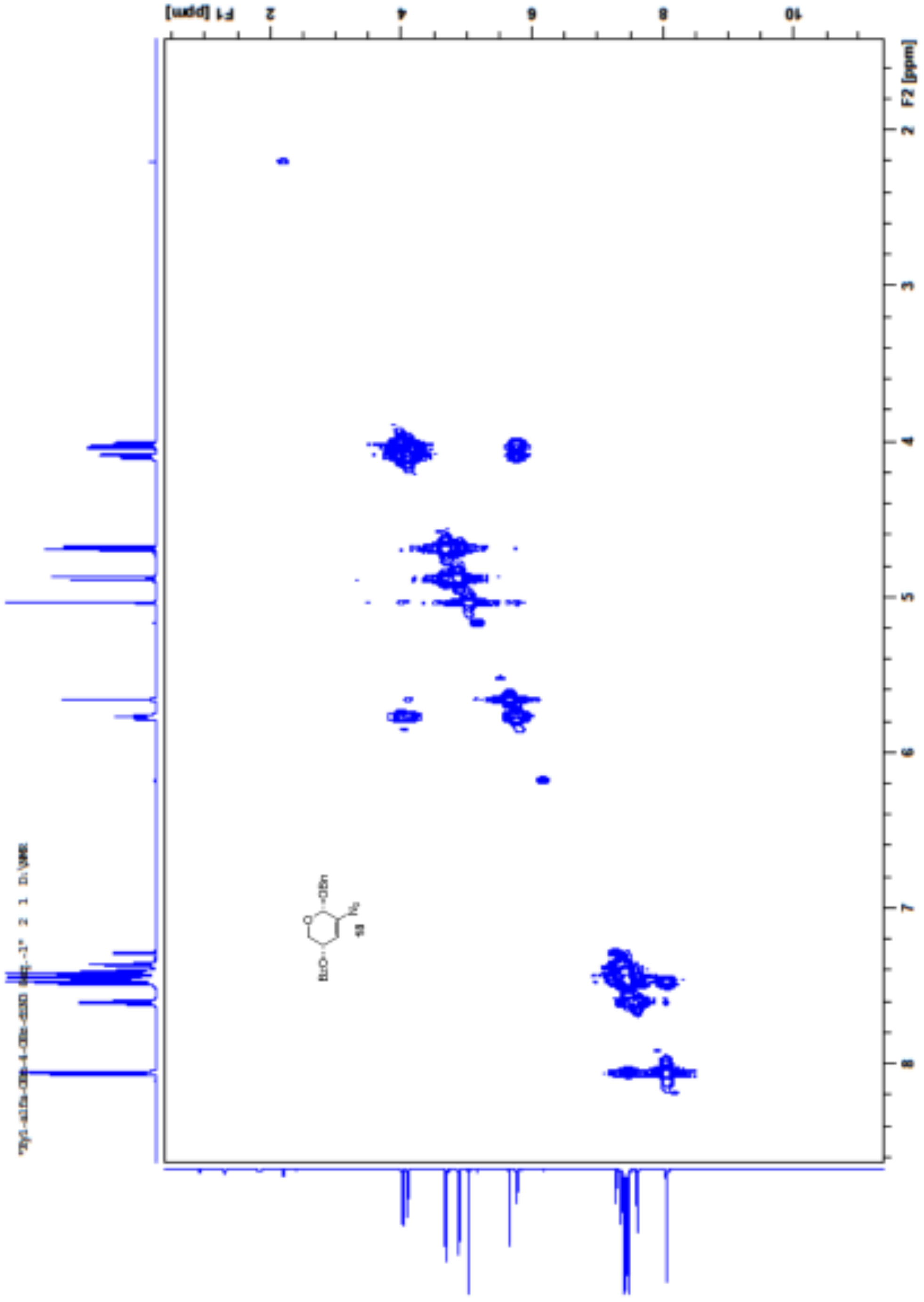

^{13}C NMR DEPT Spectroscopy of Benzyl 2-azide-2,3-di-deoxy-3-dehydro-4-*O*-benzoly-α-D-lyxopyranoside (18)

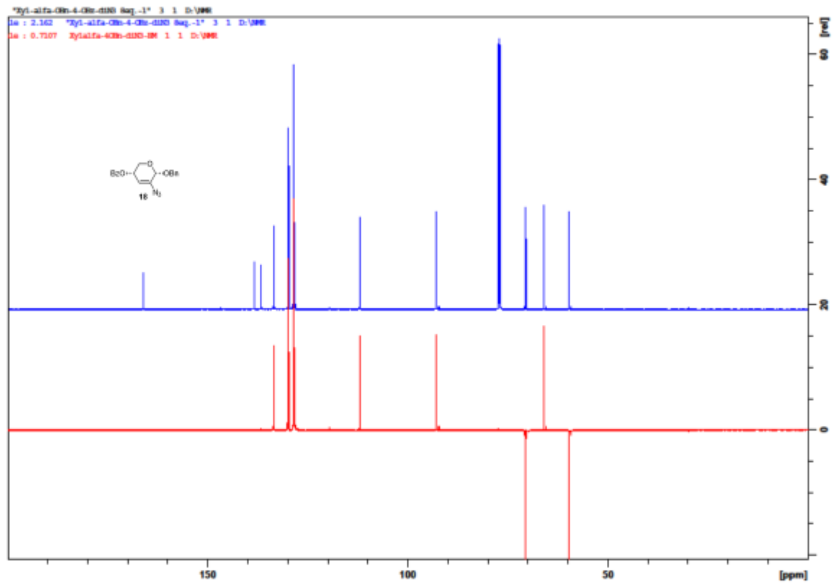

HMBC&HSQC NMR Spectroscopy of Benzyl 2-azide-2,3-di-deoxy-3-dehydro-4-*O*-benzoly-α-D-lyxopyranoside (18)

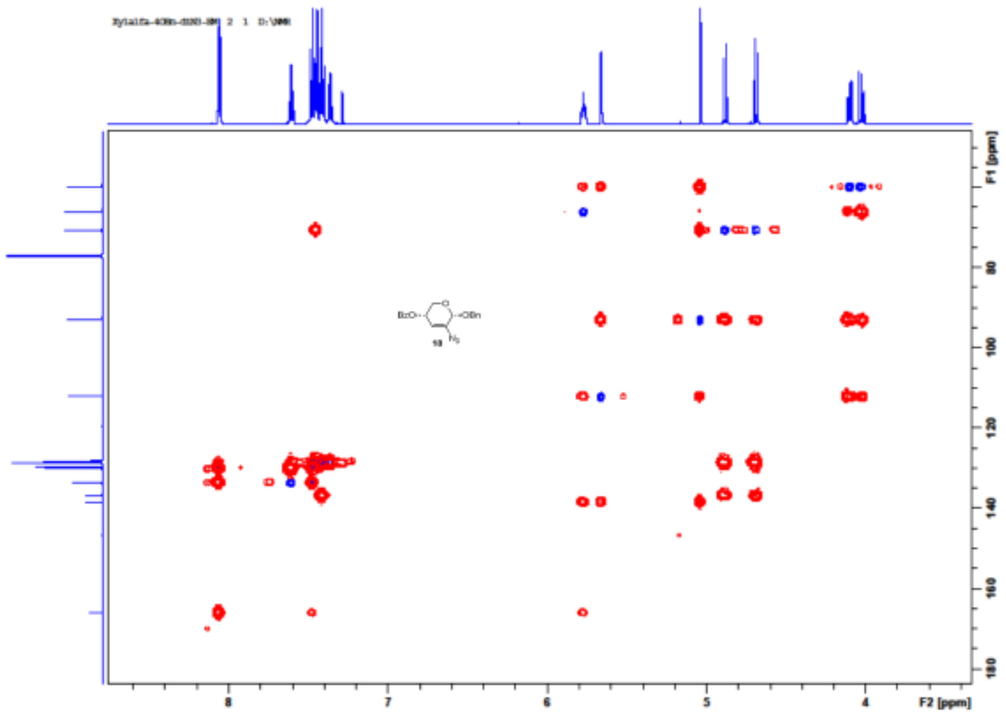

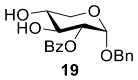

19

^{1}H NMR Spectroscopy of Benzyl 2-*O*-benzoly-α-D-xylopyranoside (19)

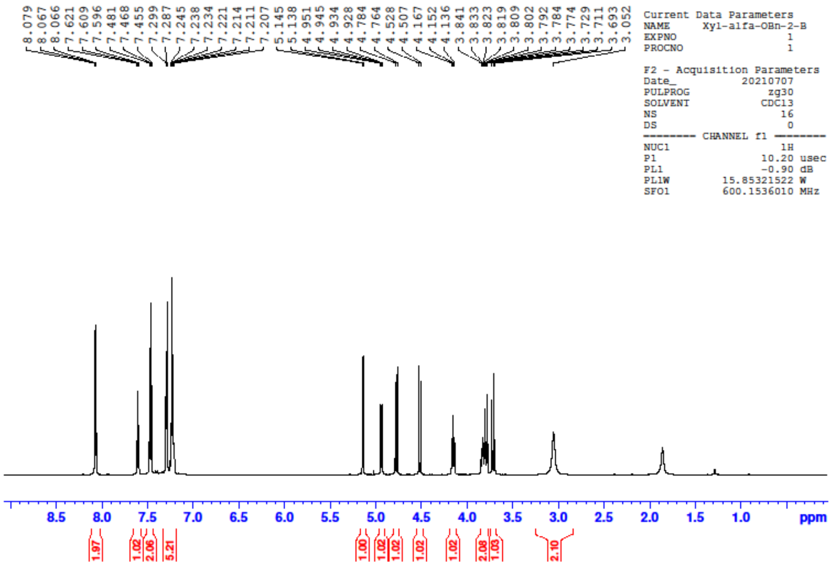

^{13}C NMR Spectroscopy of Benzyl 2-*O*-benzoly-α-D-xylopyranoside (19)

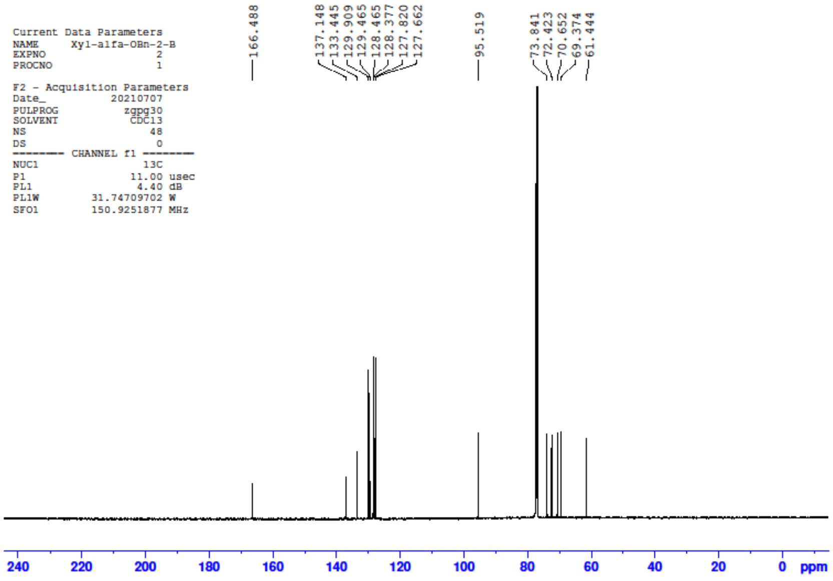

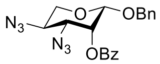

20

^{1}H NMR Spectroscopy of Benzyl 2-*O*-benzoly-3,4-di-azide-*β*-L-lyxopyranoside (20)

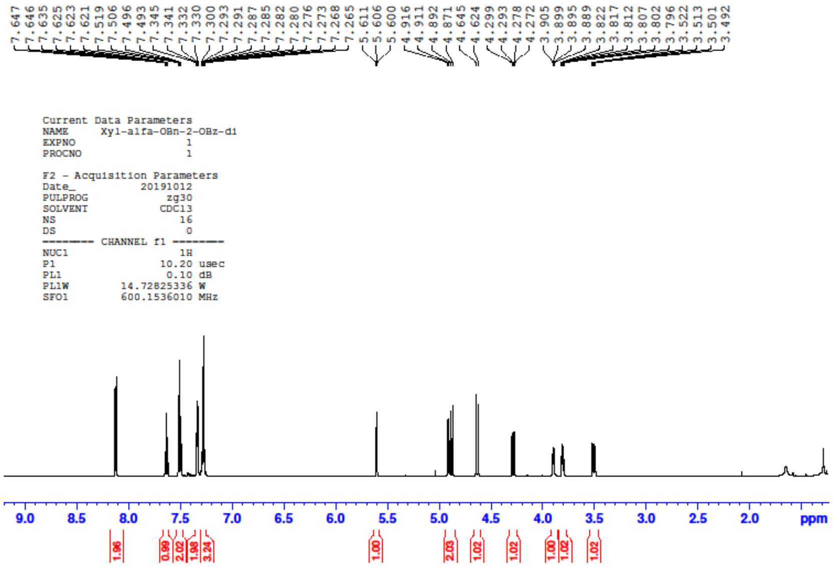

^{13}C NMR Spectroscopy of Benzyl 2-*O*-benzoly-3,4-di-azide-*β*-L-lyxopyranoside (20)

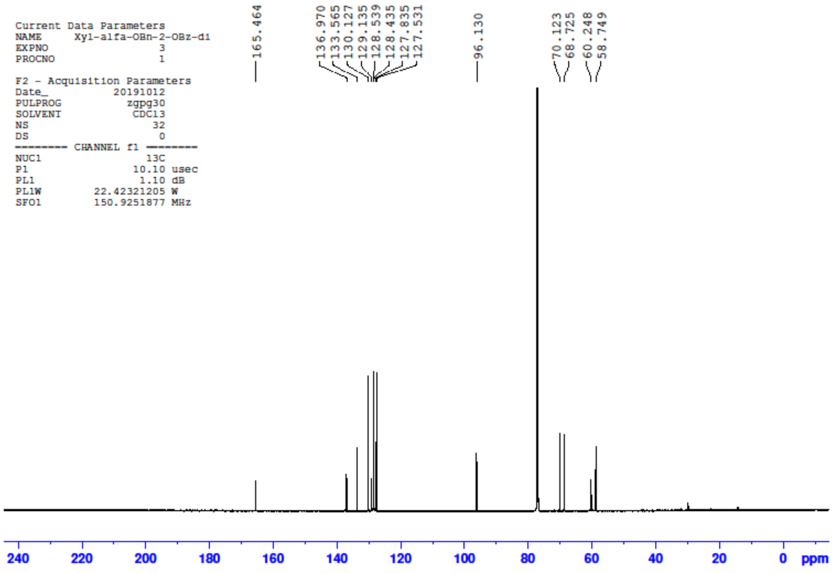

COSY NMR Spectroscopy of Benzyl 2-*O*-benzoly-3,4-di-azide-*β*-L-lyxopyranoside (20)

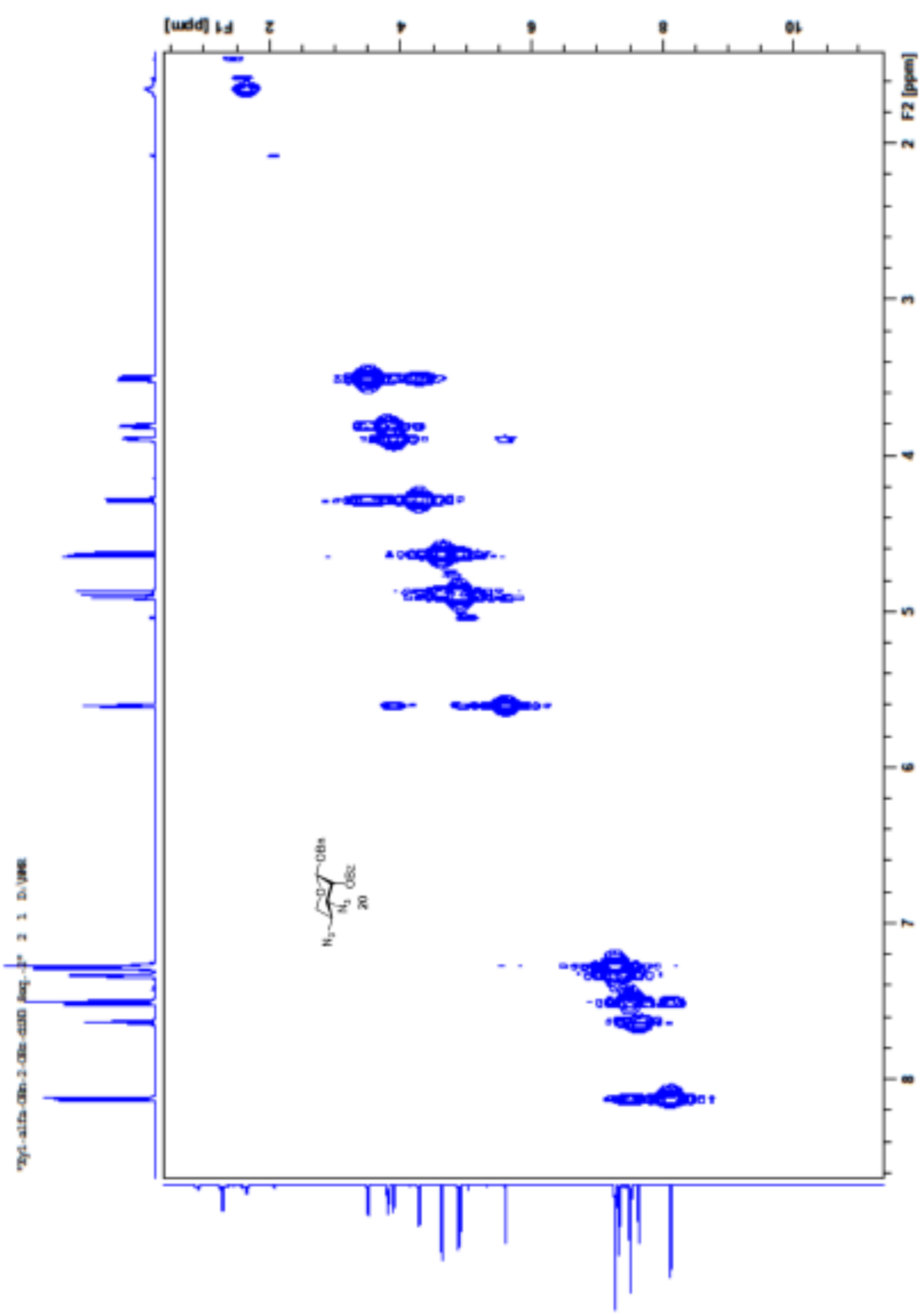

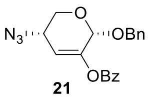

21

^{1}H NMR Spectroscopy of Benzyl 2-*O*-benzoly-3,4-di-deoxy-4-azide-4-dehydro-α-D-arabinopyranoside (21)

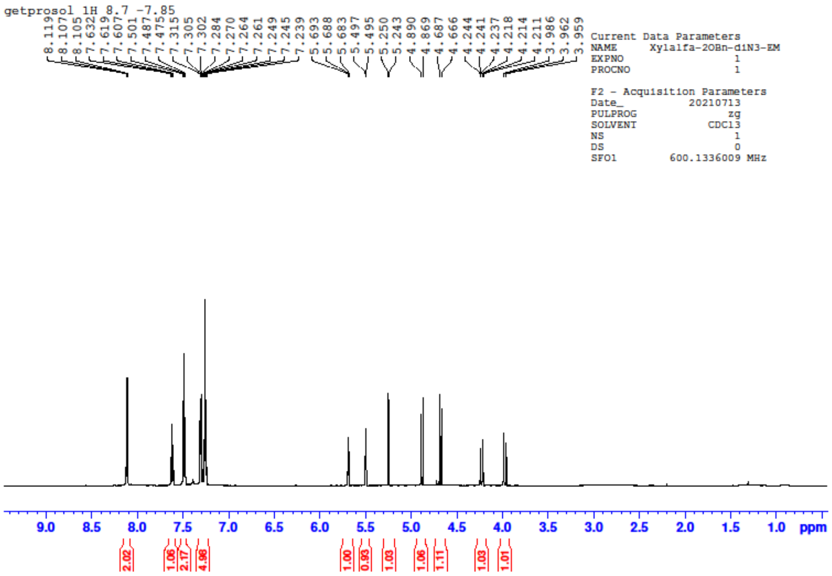

^{13}C NMR Spectroscopy of Benzyl 2-*O*-benzoly-3,4-di-deoxy-4-azide-4-dehydro-α-D-arabinopyranoside (21)

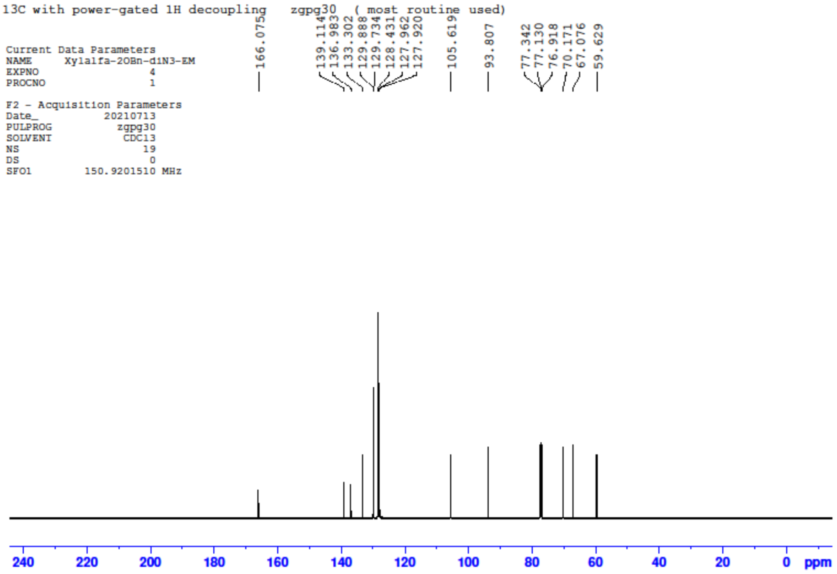

COSY NMR Spectroscopy of Benzyl 2-*O*-benzoly-3,4-di-deoxy-4-azide-4-dehydro-α-D-arabinopyranoside (21)

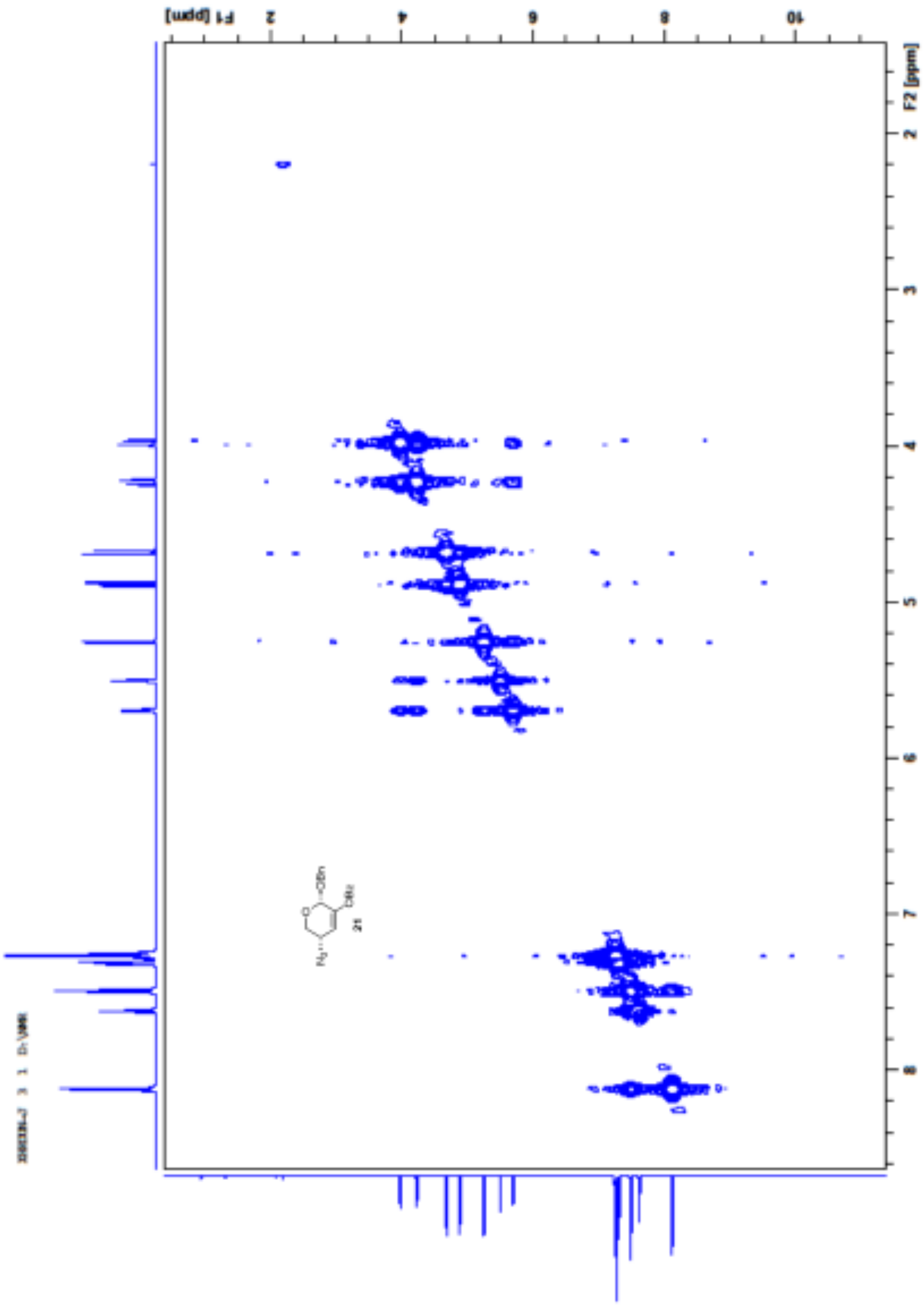

^{13}C NMR DEPT Spectroscopy of Benzyl 2-*O*-benzoly-3,4-di-deoxy-4-azide-4-dehydro-α-D-arabinopyranoside (21)

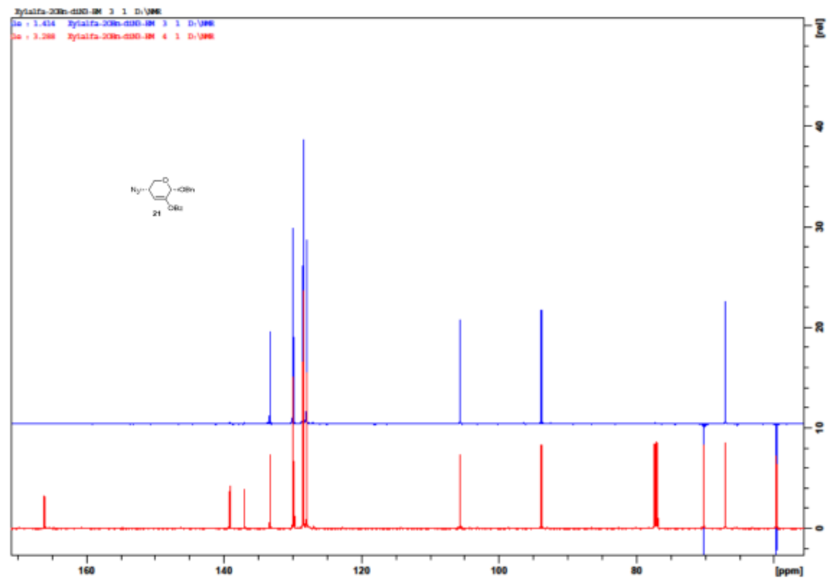

HMBC&HSQC NMR Spectroscopy of Benzyl 2-*O*-benzoly-3,4-di-deoxy-4-azide-4-dehydro-α-D-arabinopyranoside (21)

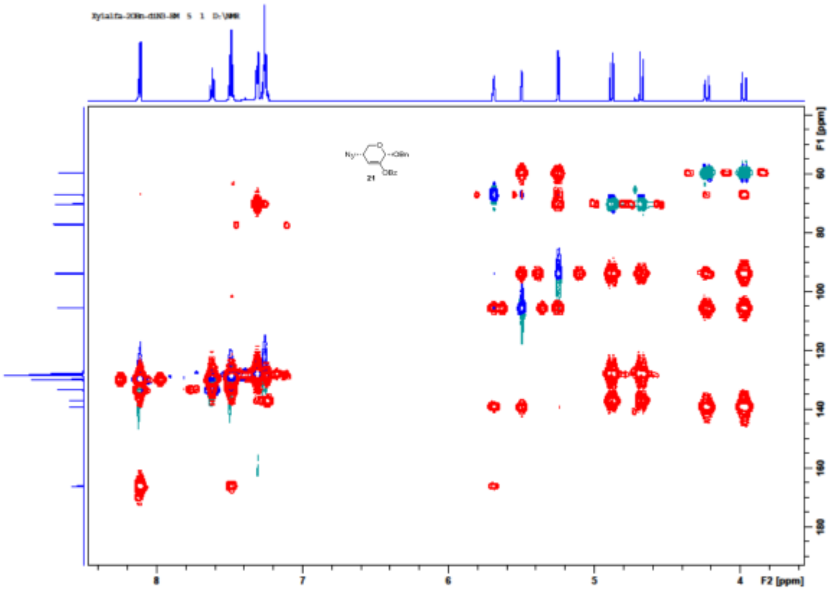

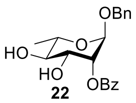

22

^{1}H NMR Spectroscopy of Benzyl 2-*O*-benzoyl-α-L-rhamnopyranoside (22)

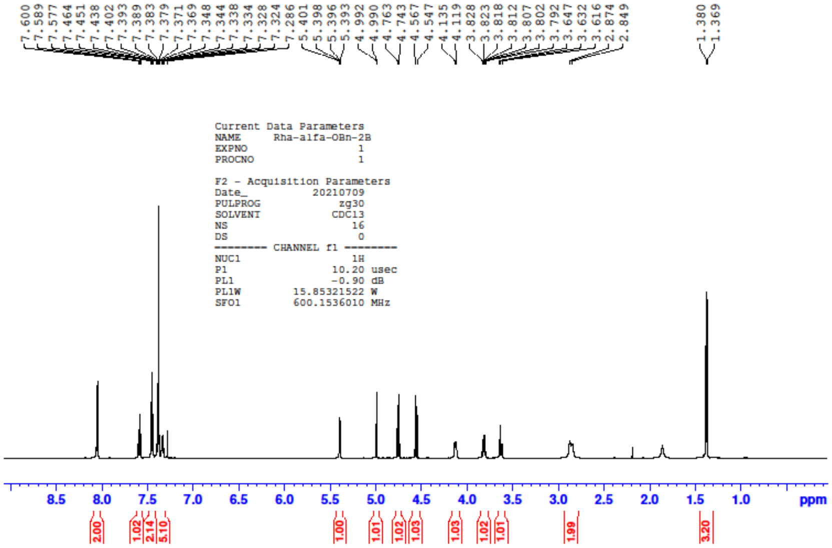

^{13}C NMR Spectroscopy of Benzyl 2-*O*-benzoyl-α-L-rhamnopyranoside (22)

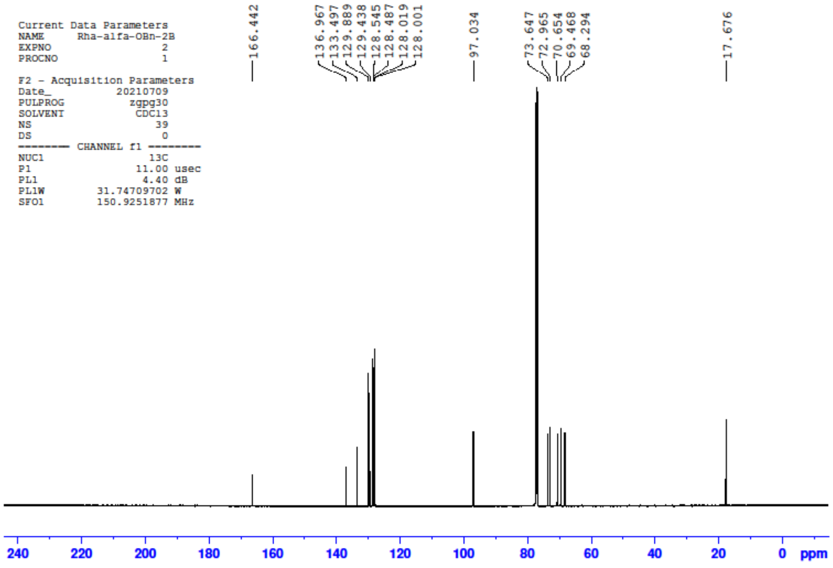

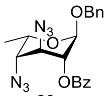

23

^{1}H NMR Spectroscopy of Benzyl 2-*O*-benzoly-3,4-di-azide-6-deoxyl-α-L-idopyranoside (23)

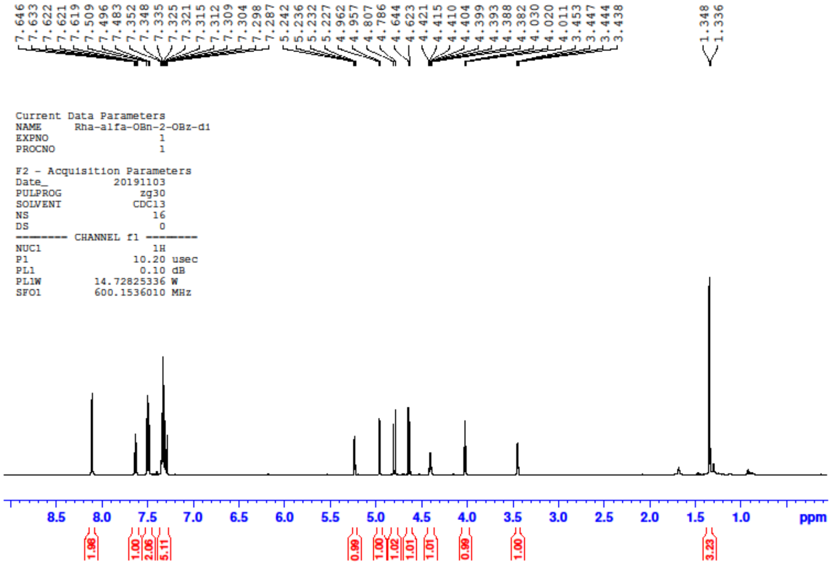

^{13}C NMR Spectroscopy of Benzyl 2-*O*-benzoly-3,4-di-azide-6-deoxyl-α-L-idopyranoside (23)

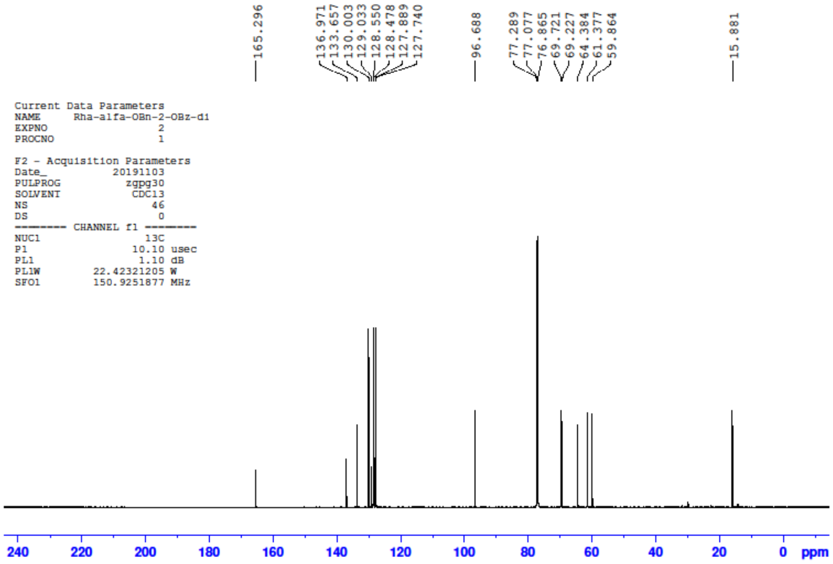

COSY NMR Spectroscopy of Benzyl 2-*O*-benzoly-3,4-di-azide-6-deoxyl-α-L-idopyranoside (23)

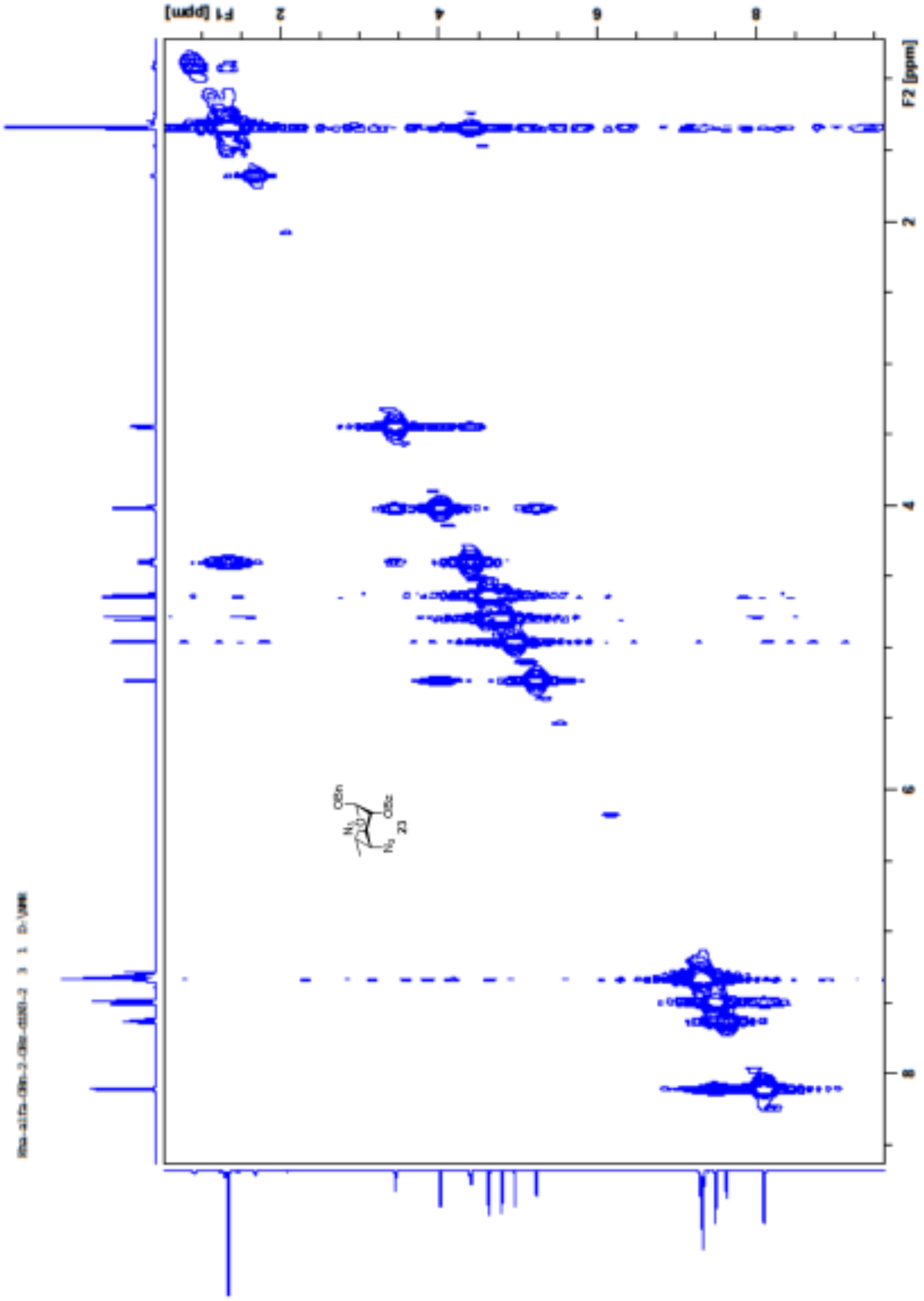

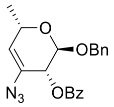

24

^{1}H NMR Spectroscopy of Benzyl 2-*O*-benzoly-3-azide-3,4,6-tri-deoxy-3-dehydro-α-L-altropyranoside (24)

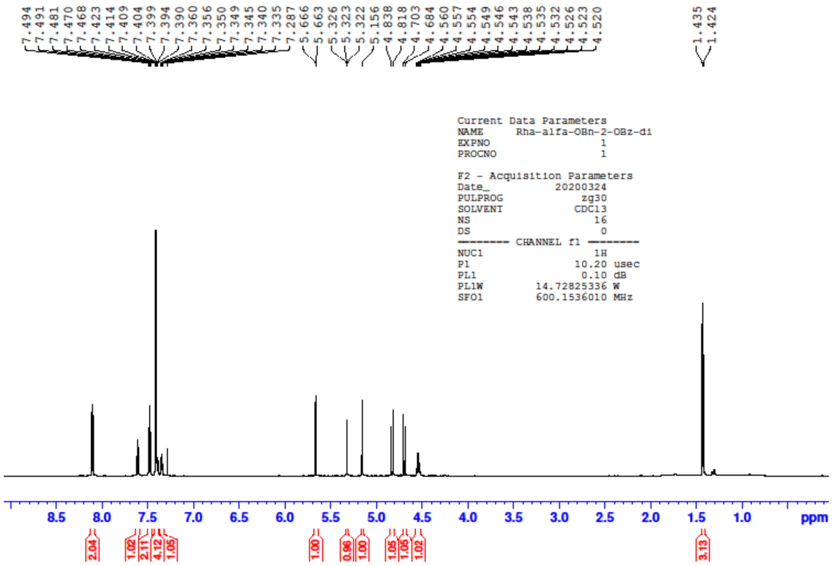

^{13}C NMR Spectroscopy of Benzyl 2-*O*-benzoly-3-azide-3,4,6-tri-deoxy-3-dehydro-α-L-altropyranoside (24)

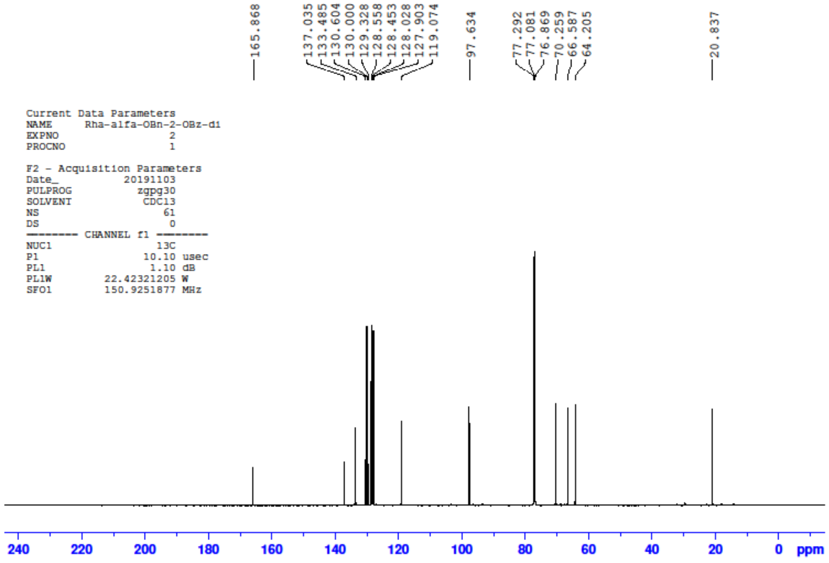

COSY NMR Spectroscopy of Benzyl 2-*O*-benzoly-3-azide-3,4,6-tri-deoxy-3-dehydro-α-L-altropyranoside (24)

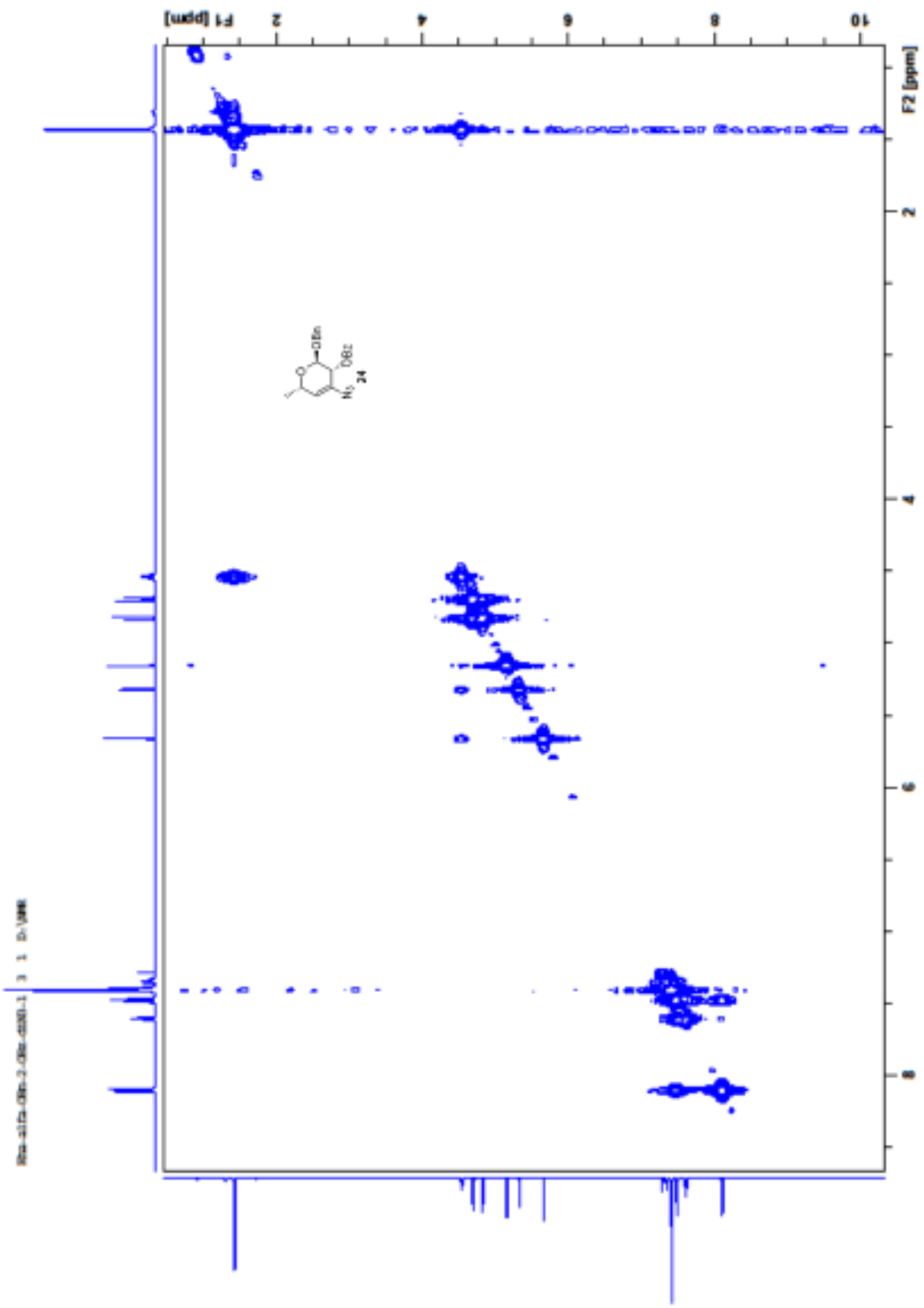

^{13}C NMR DEPT Spectroscopy of Benzyl 2-*O*-benzoly-3-azide-3,4,6-tri-deoxy-3-dehydro-α-L-altropyranoside (24)

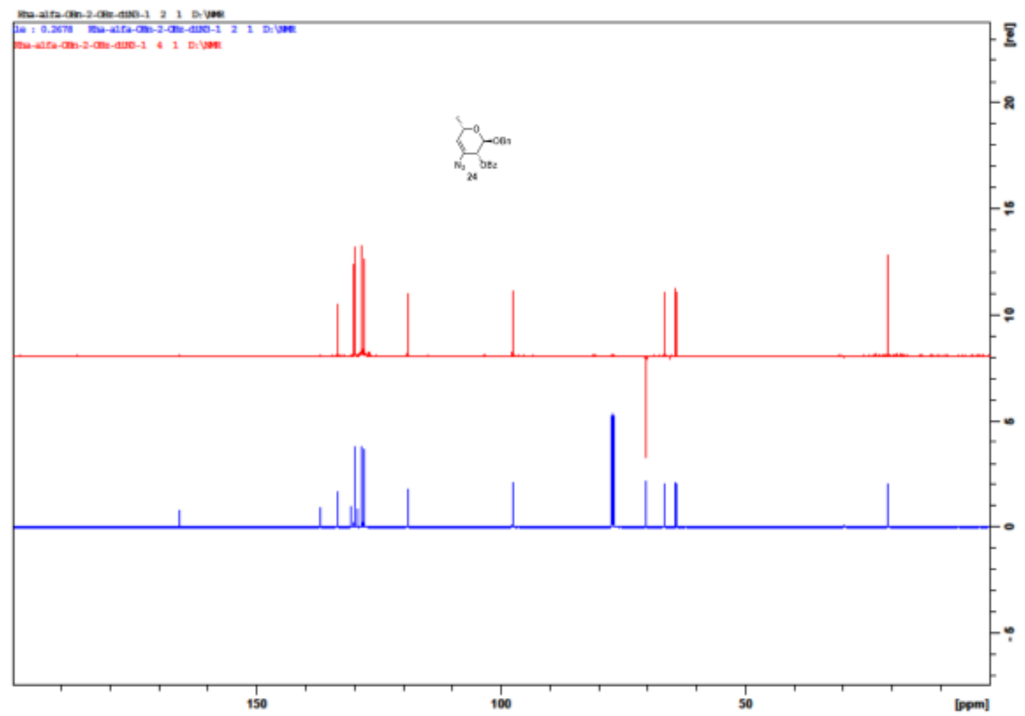

HMBC&HSQC NMR Spectroscopy of Benzyl 2-*O*-benzoly-3-azide-3,4,6-tri-deoxy-3-dehydro-α-L-altropyranoside (24)

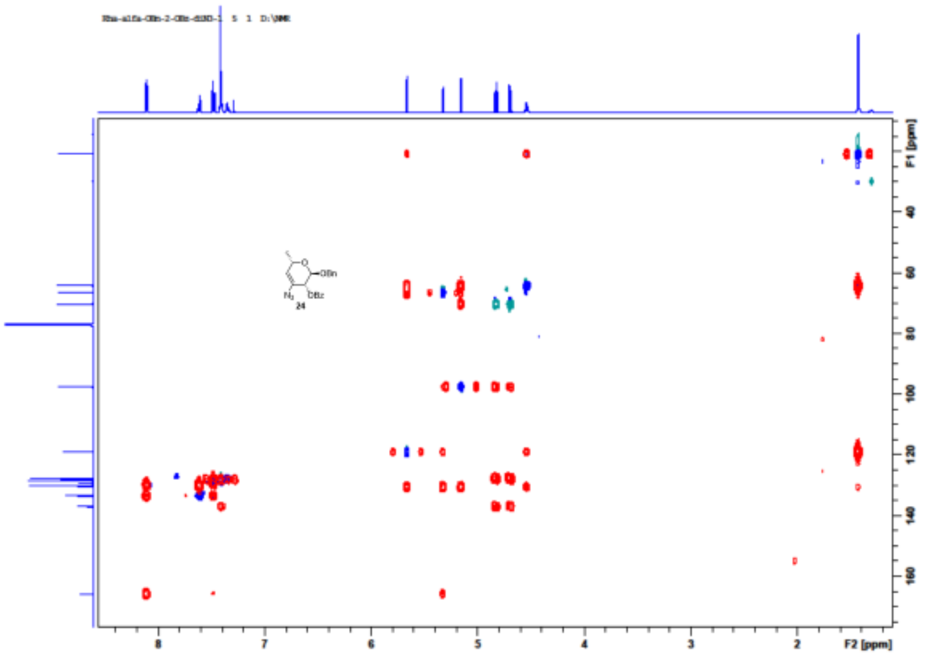

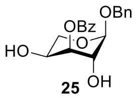

25

^{1}H NMR Spectroscopy of Benzyl 3-*O*-benzoyl-α-L-Arabinopyranoside (25)

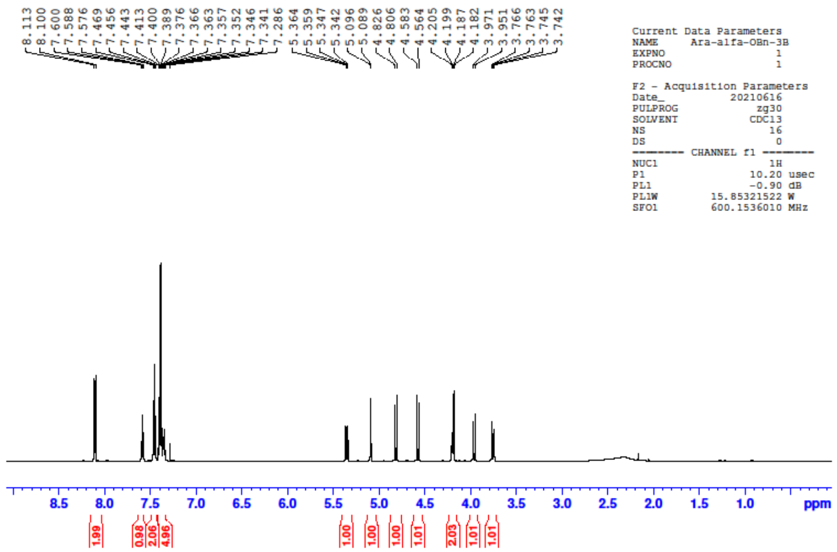

^{13}C NMR Spectroscopy of Benzyl 3-*O*-benzoyl-α-L-Arabinopyranoside (25)

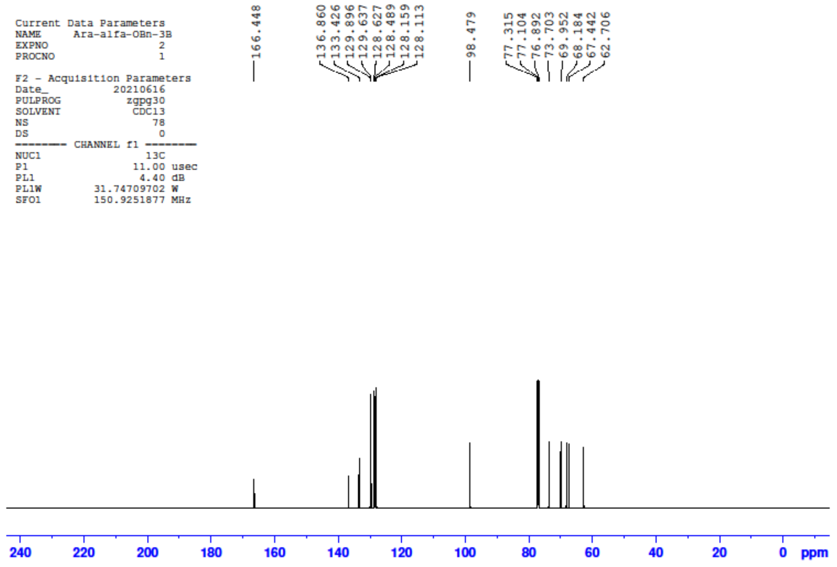

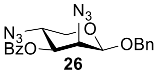

26

^{1}H NMR Spectroscopy of Benzyl 3-*O*-benzoly-2,4-di-azide-*β*-D-lyxopyranoside (26)

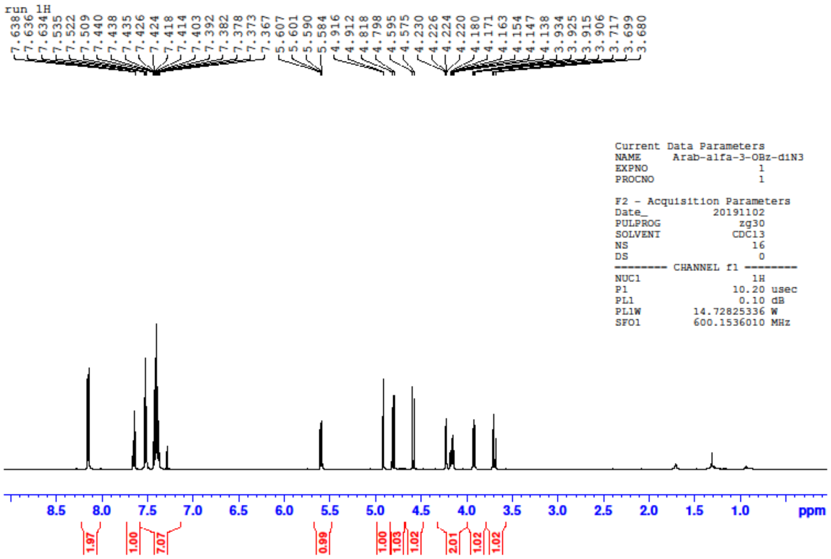

^{13}C NMR Spectroscopy of Benzyl 3-*O*-benzoly-2,4-di-azide-*β*-D-lyxopyranoside (26)

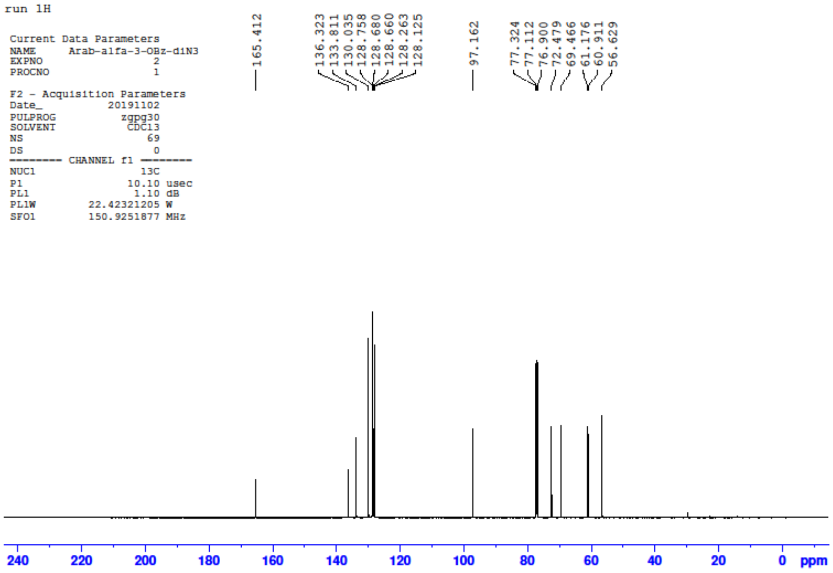

COSY NMR Spectroscopy of Benzyl 3-*O*-benzoly-2,4-di-azide-*β*-D-lyxopyranoside (26)

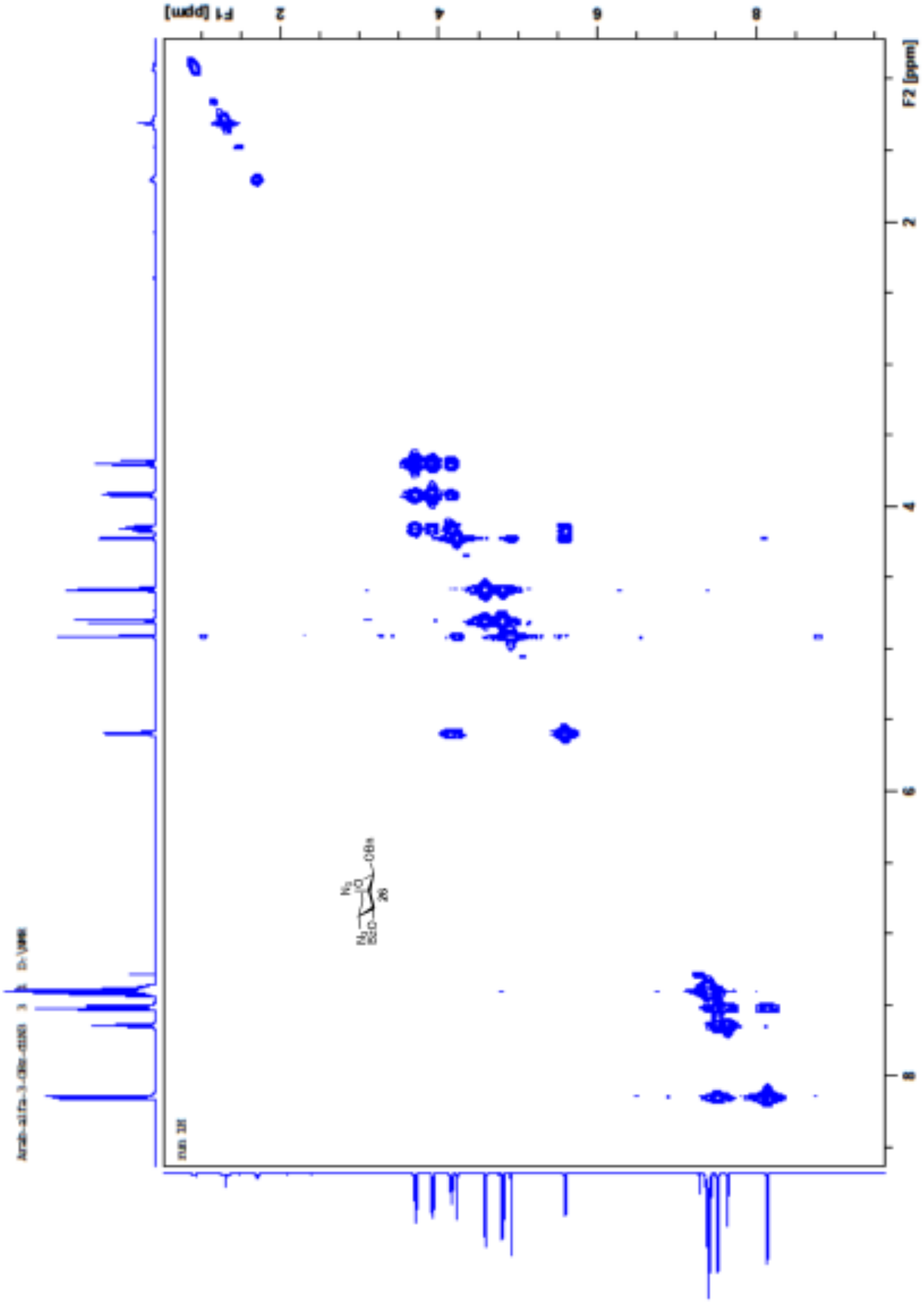

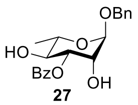

27

^{1}H NMR Spectroscopy of Benzyl 3-*O*-benzoyl-α-L-rhamnopyranoside (27)

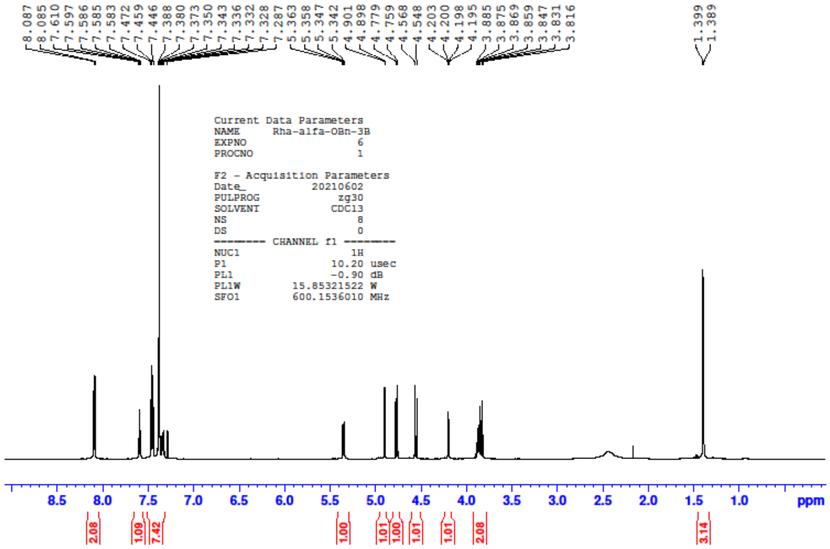

^{13}C NMR Spectroscopy of Benzyl 3-*O*-benzoyl-α-L-rhamnopyranoside (27)

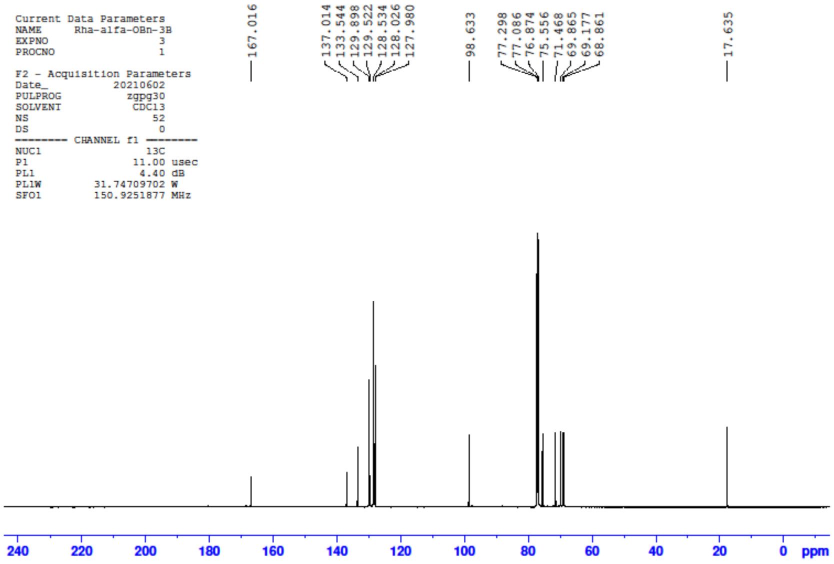

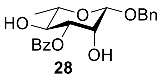

28

^{1}H NMR Spectroscopy of Benzyl 3-*O*-benzoyl-*β*-L-rhamnopyranoside (28)

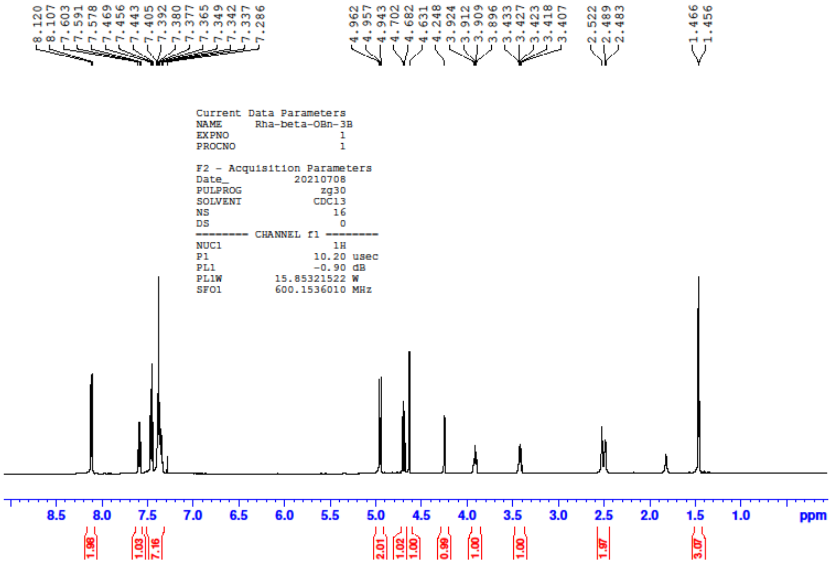

^{13}C NMR Spectroscopy of Benzyl 3-*O*-benzoyl-*β*-L-rhamnopyranoside (28)

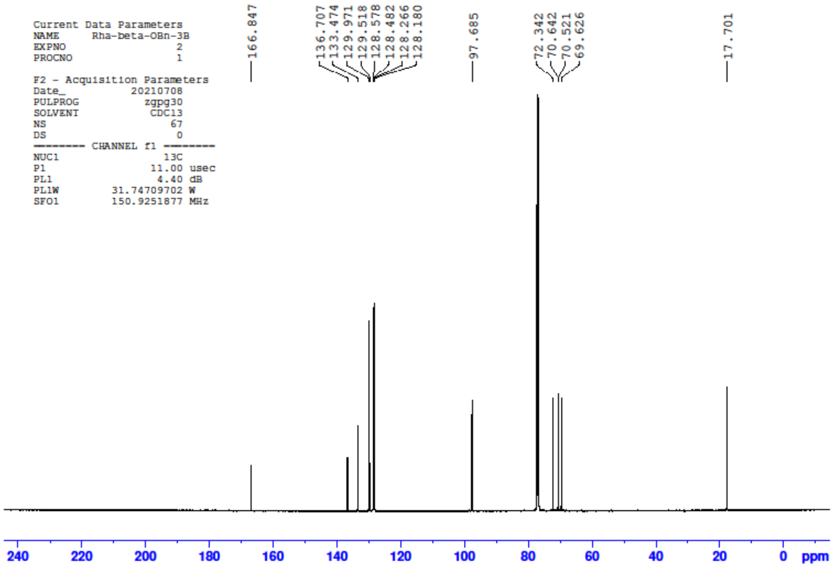

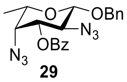

29

^{1}H NMR Spectroscopy of Benzyl 3-*O*-benzoyl-2,4-di-azide-*β*-L-fucopyranoside (29)

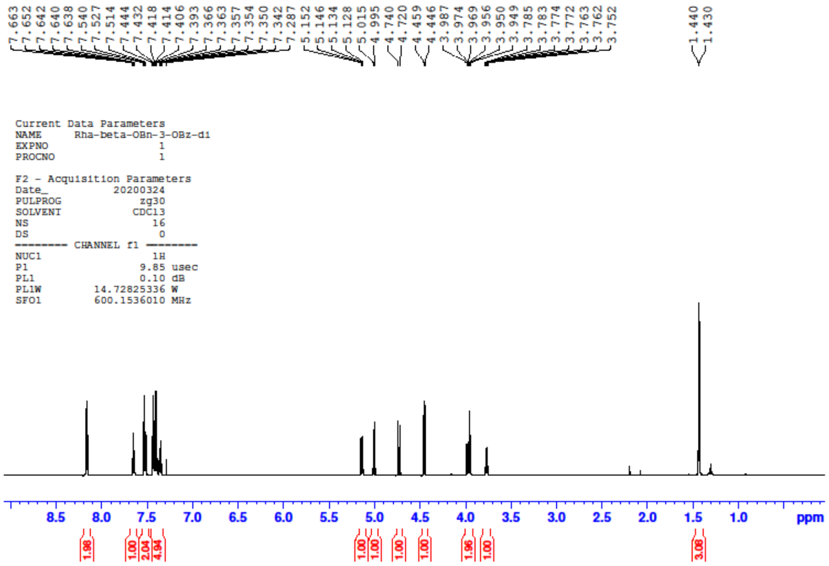

^{13}C NMR Spectroscopy of Benzyl 3-*O*-benzoyl-2,4-di-azide-*β*-L-fucopyranoside (29)

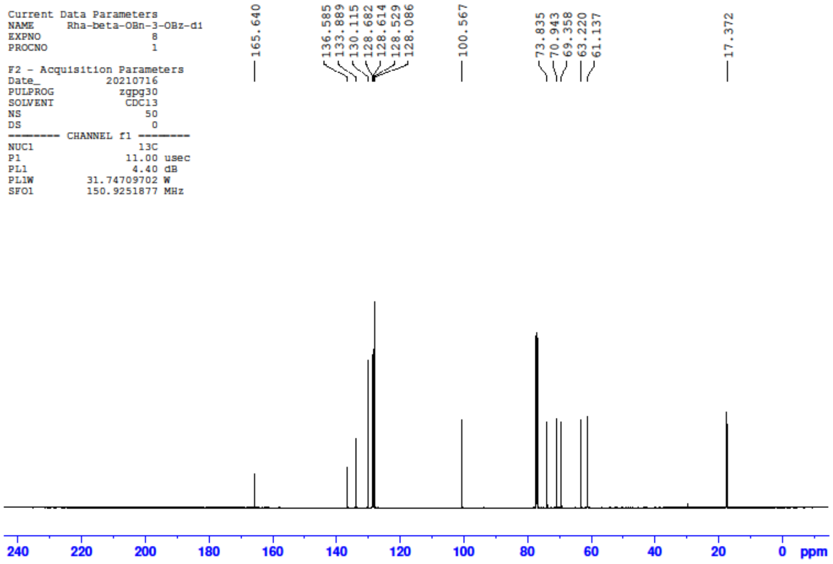

COSY NMR Spectroscopy of Benzyl 3-*O*-benzoyl-2,4-di-azide-*β*-L-fucopyranoside (29)

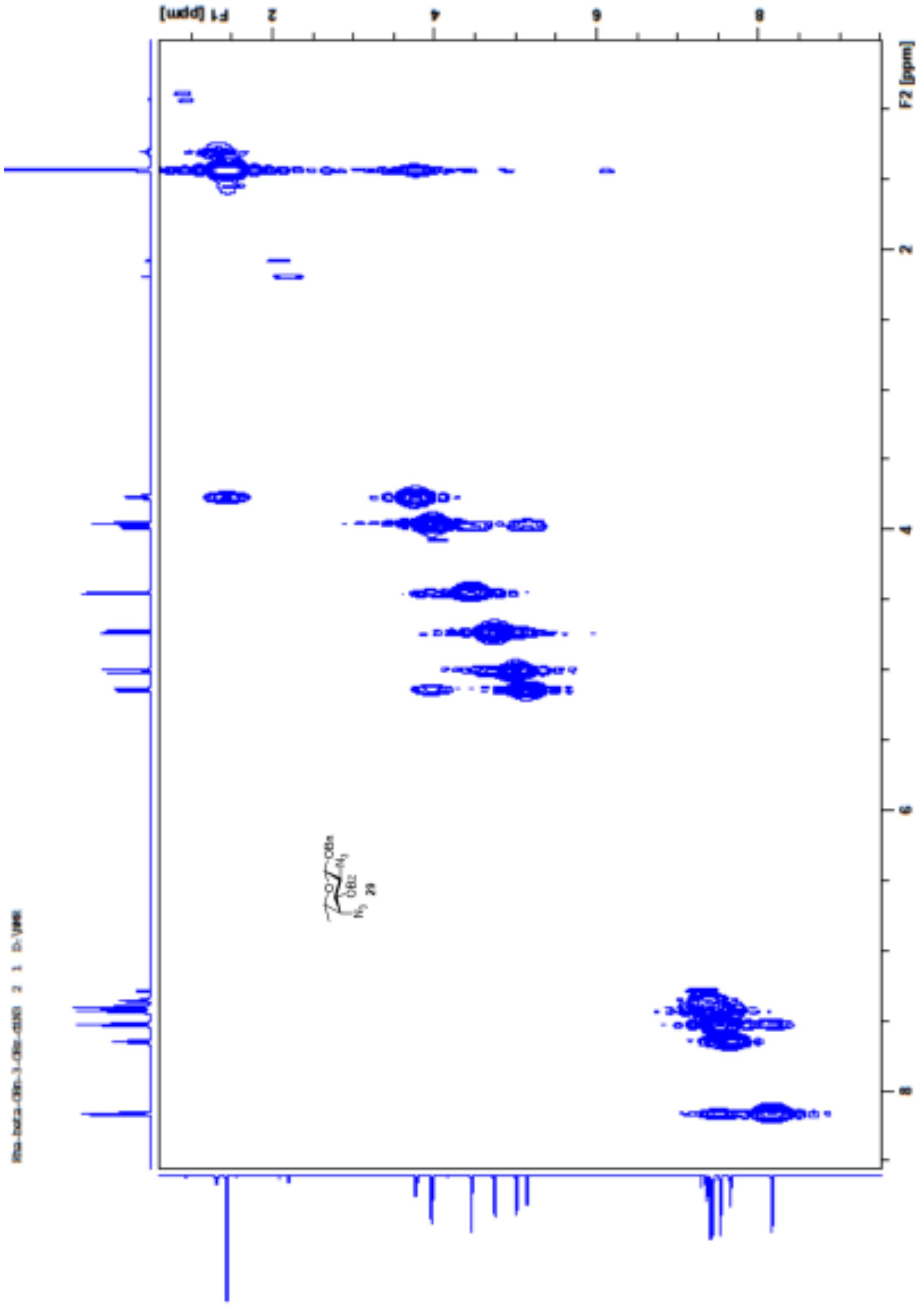

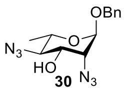

^{1}H NMR Spectroscopy of Benzyl 2,4-di-azide-α-L-rhamnopyranoside (30)

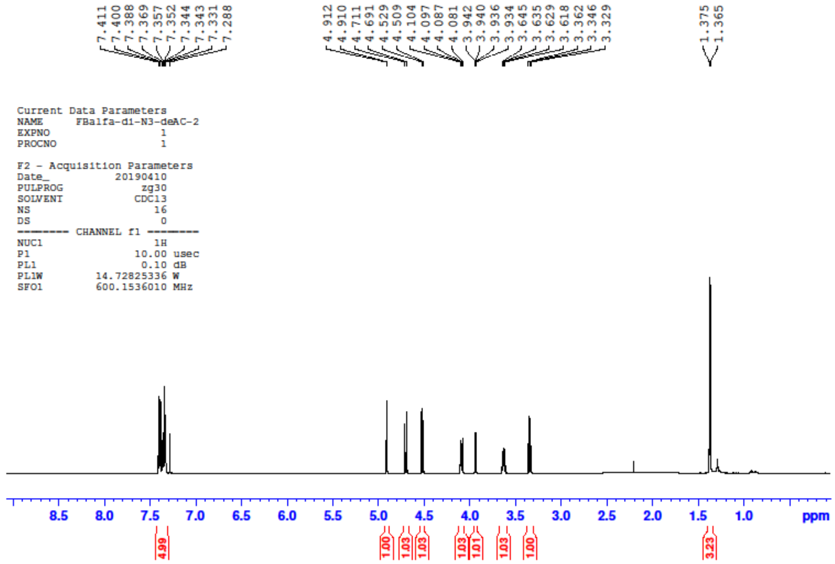

^{13}C NMR Spectroscopy of Benzyl 2,4-di-azide-α-L-rhamnopyranoside (30)

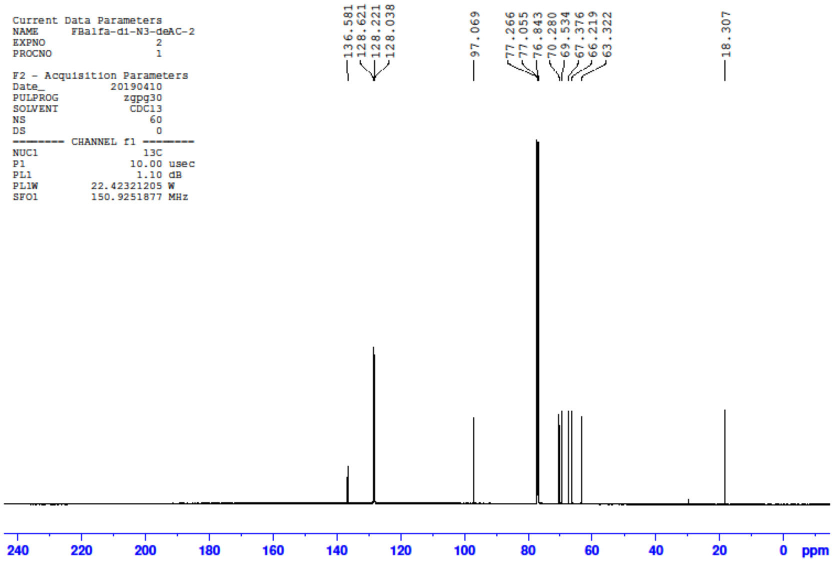

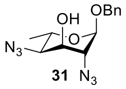

^{1}H NMR Spectroscopy of Benzyl 2,4-di-azide-6-deoxy-α-L- altropyranoside (31)

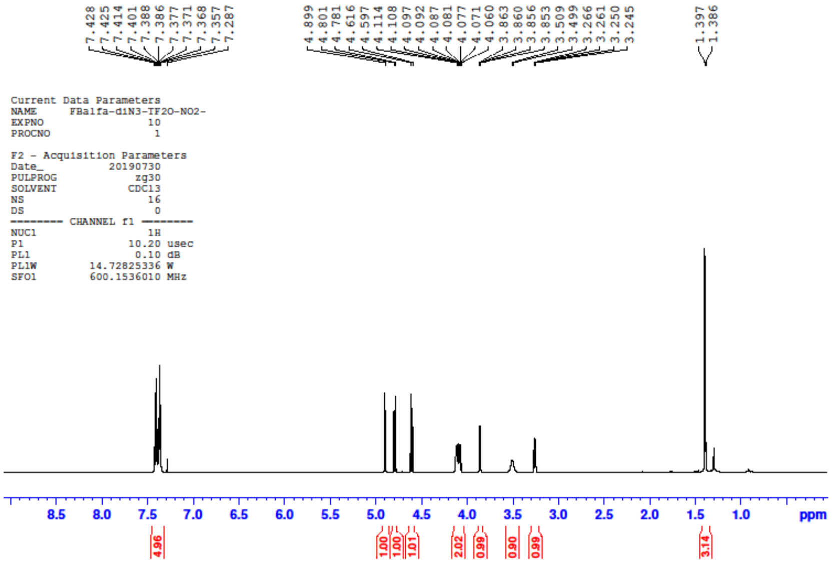

^{13}C NMR Spectroscopy of Benzyl 2,4-di-azide-6-deoxy-α-L- altropyranoside (31)

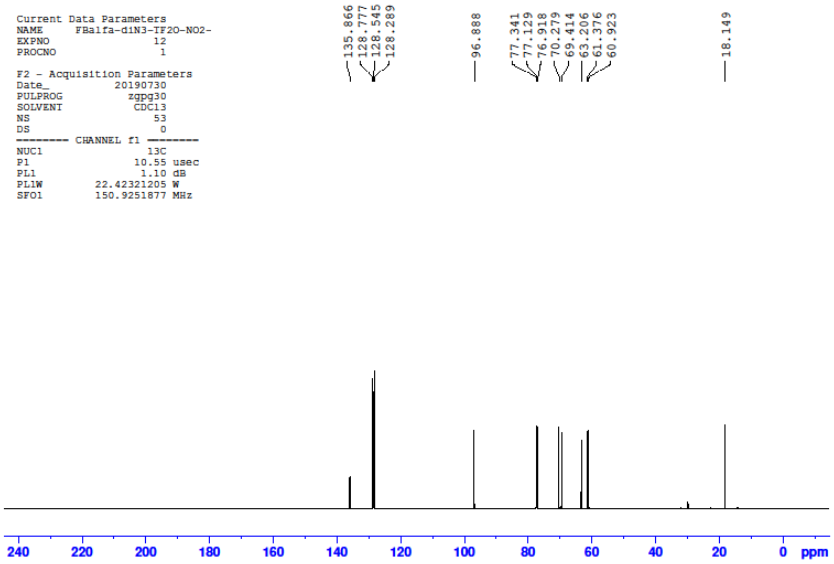

COSY NMR Spectroscopy of Benzyl 2,4-di-azide-6-deoxy-α-L- altropyranoside (31)

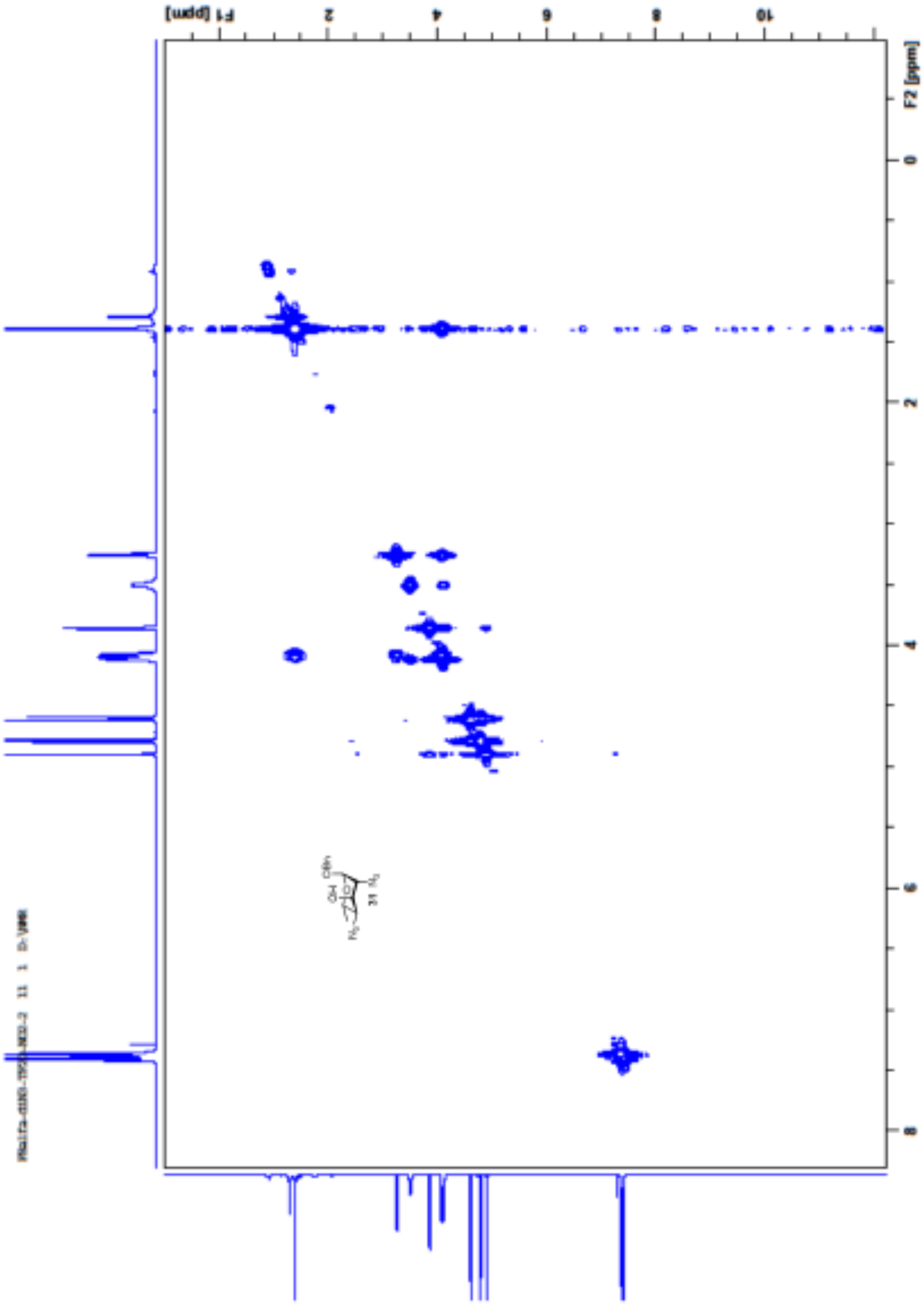

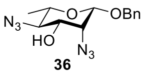

36

^{1}H NMR Spectroscopy of Benzyl 2,4-di-azide-*β*-L-rhamnopyranoside (36)

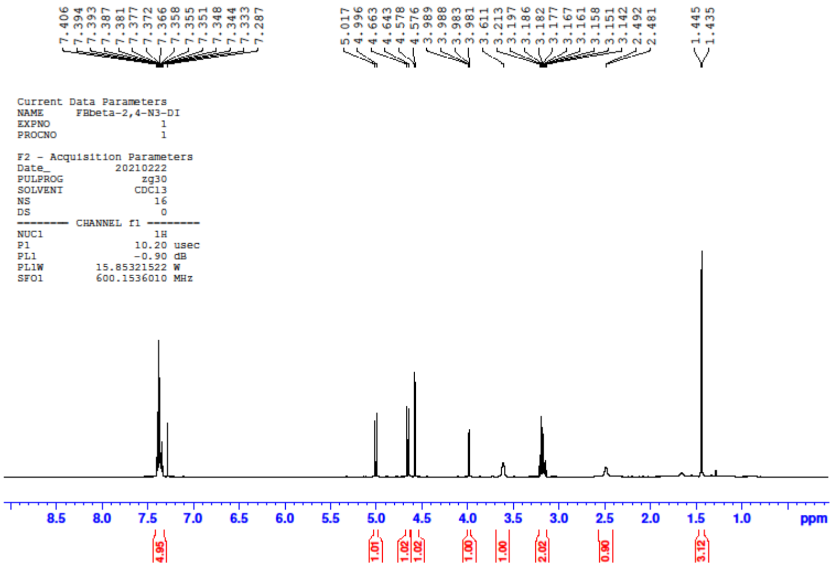

^{13}C NMR Spectroscopy of Benzyl 2,4-di-azide-*β*-L-rhamnopyranoside (36)

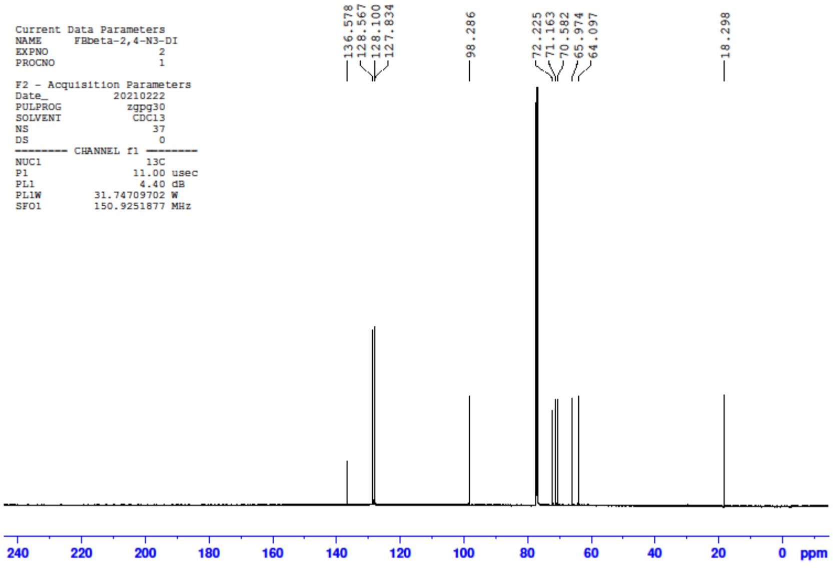

COSY NMR Spectroscopy of Benzyl 2,4-di-azide-β-L-rhamnopyranoside (36)

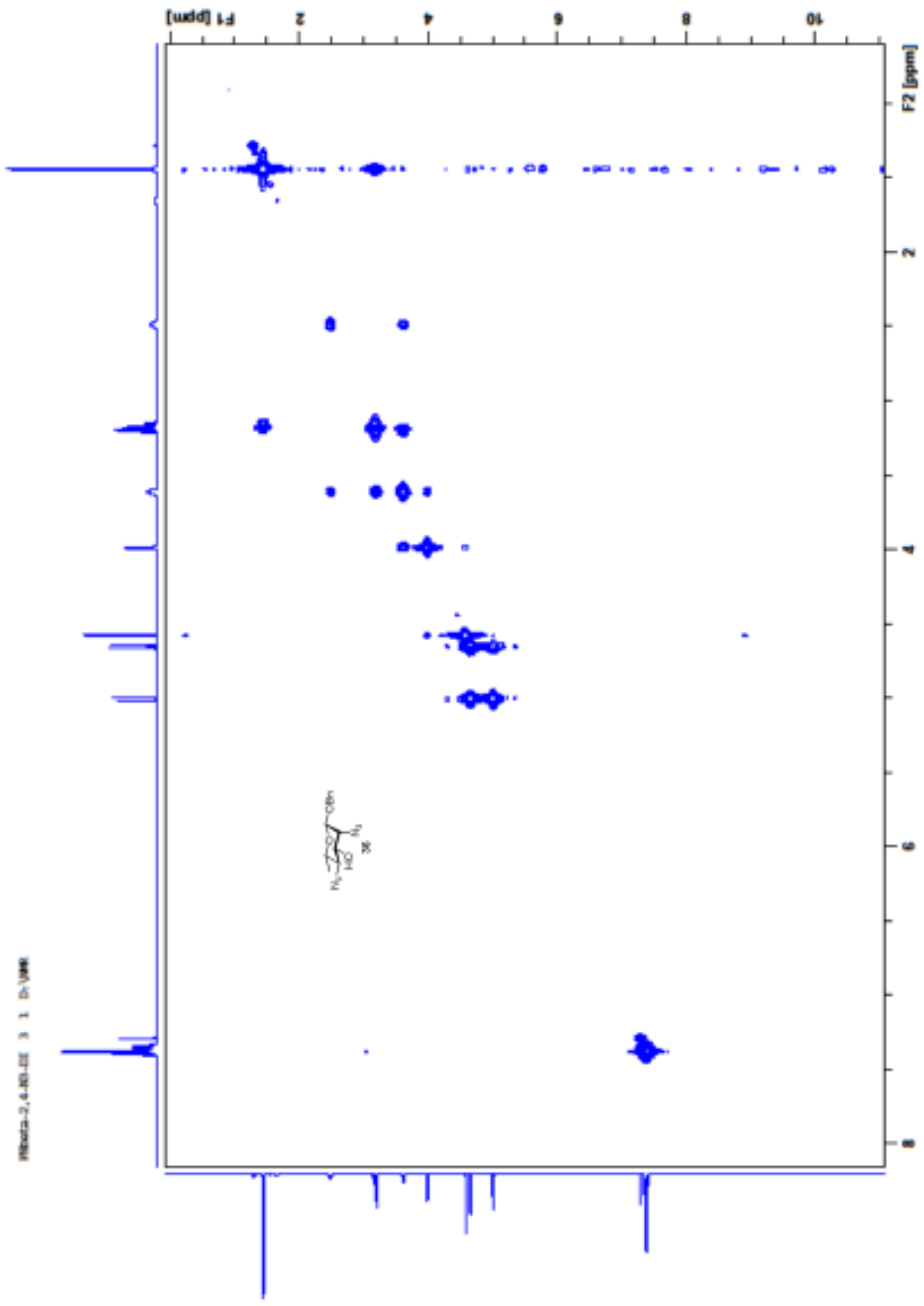

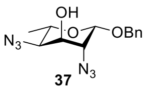

^{1}H NMR Spectroscopy of Benzyl 2,4-di-azide-2,4,6-deoxy-*β*-L-altropyranoside (37)

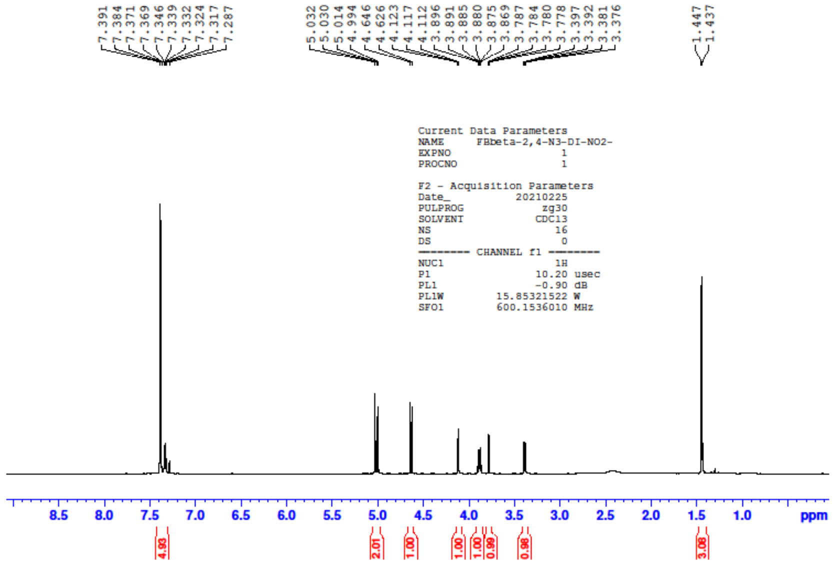

^{13}C NMR Spectroscopy of Benzyl 2,4-di-azide-2,4,6-deoxy-*β*-L-altropyranoside (37)

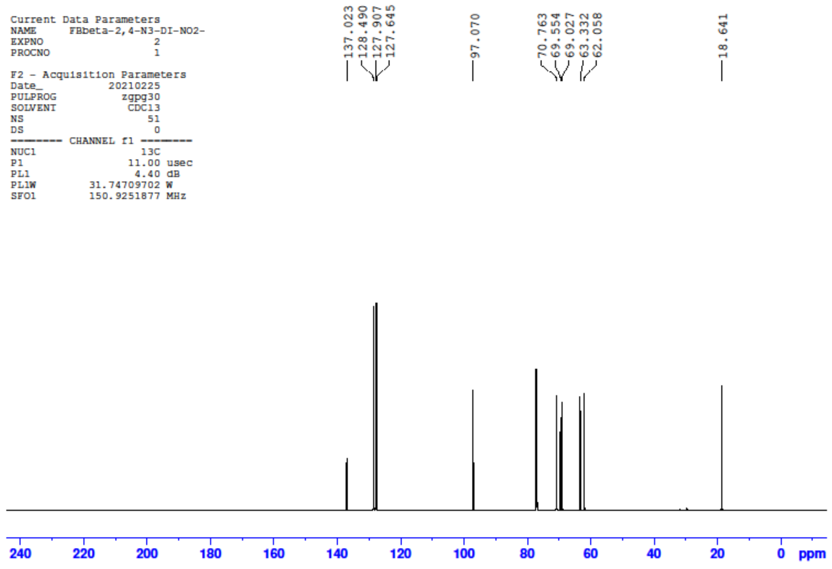

COSY NMR Spectroscopy of Benzyl 2,4-di-azide-2,4,6-deoxy-β-L-altropyranoside (37)

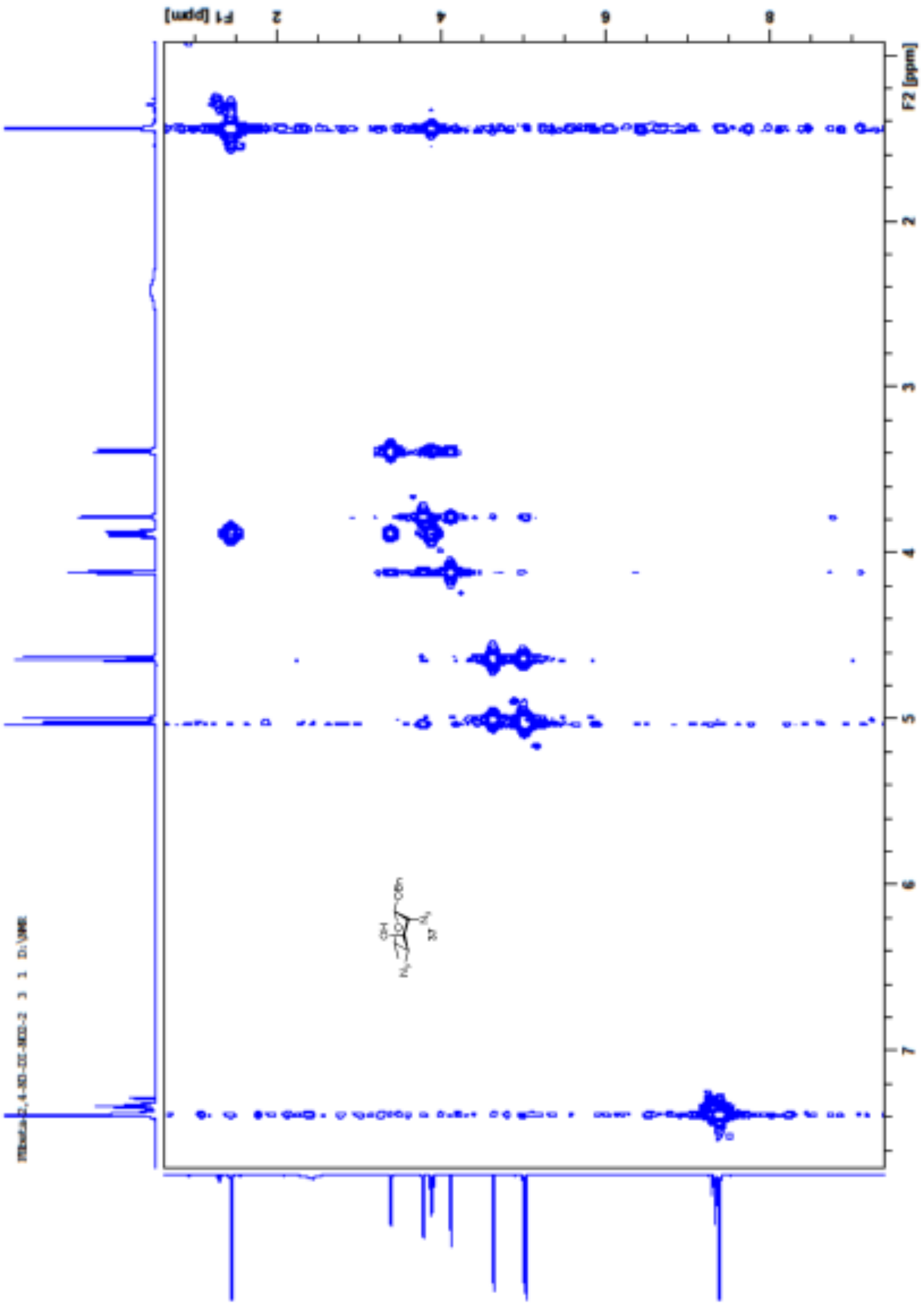

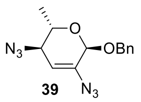

^{1}H NMR Spectroscopy of Benzyl 2,4-di-azide-2,3,4-tri-deoxy-2-dehydro-α-L-rhamnopyranoside (39)

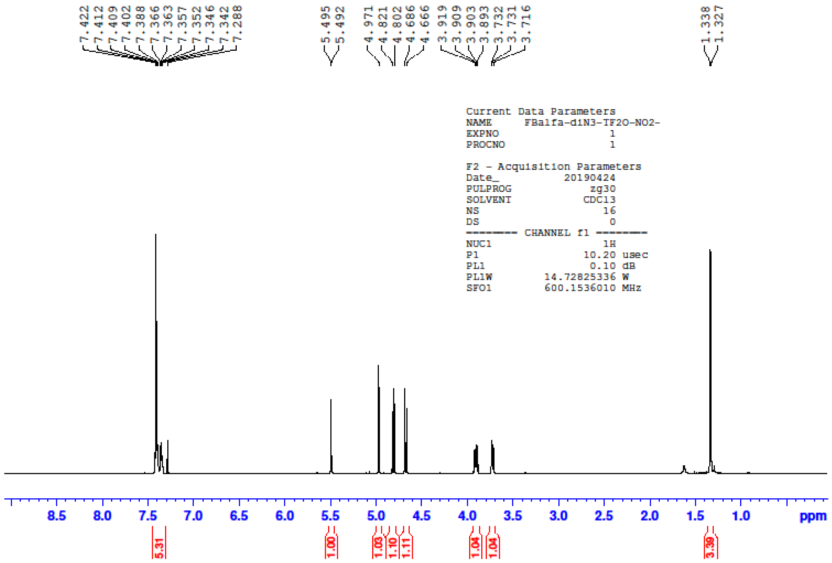

^{13}C NMR Spectroscopy of Benzyl 2,4-di-azide-2,3,4-tri-deoxy-2-dehydro-α-L-rhamnopyranoside (39)

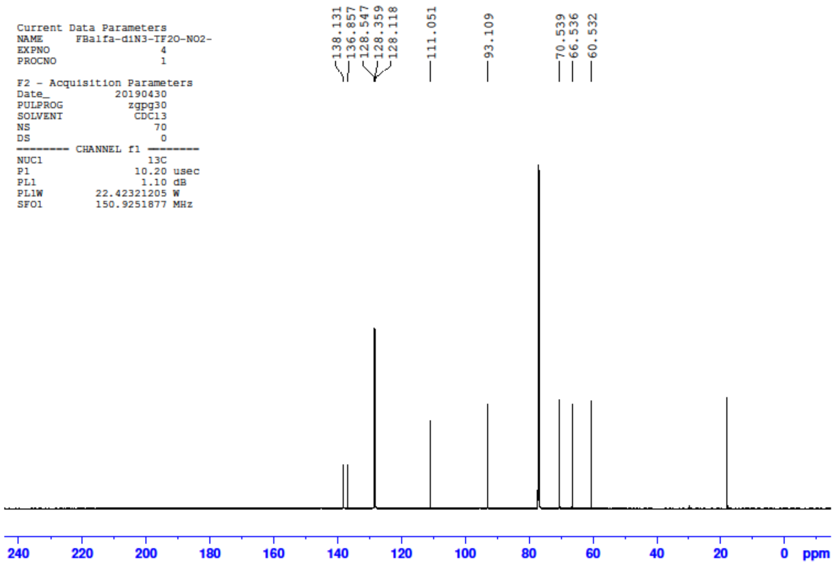

COSY NMR Spectroscopy of Benzyl 2,4-di-azide-2,3,4-tri-deoxy-2-dehydro-α-L-rhamnopyranoside (39)

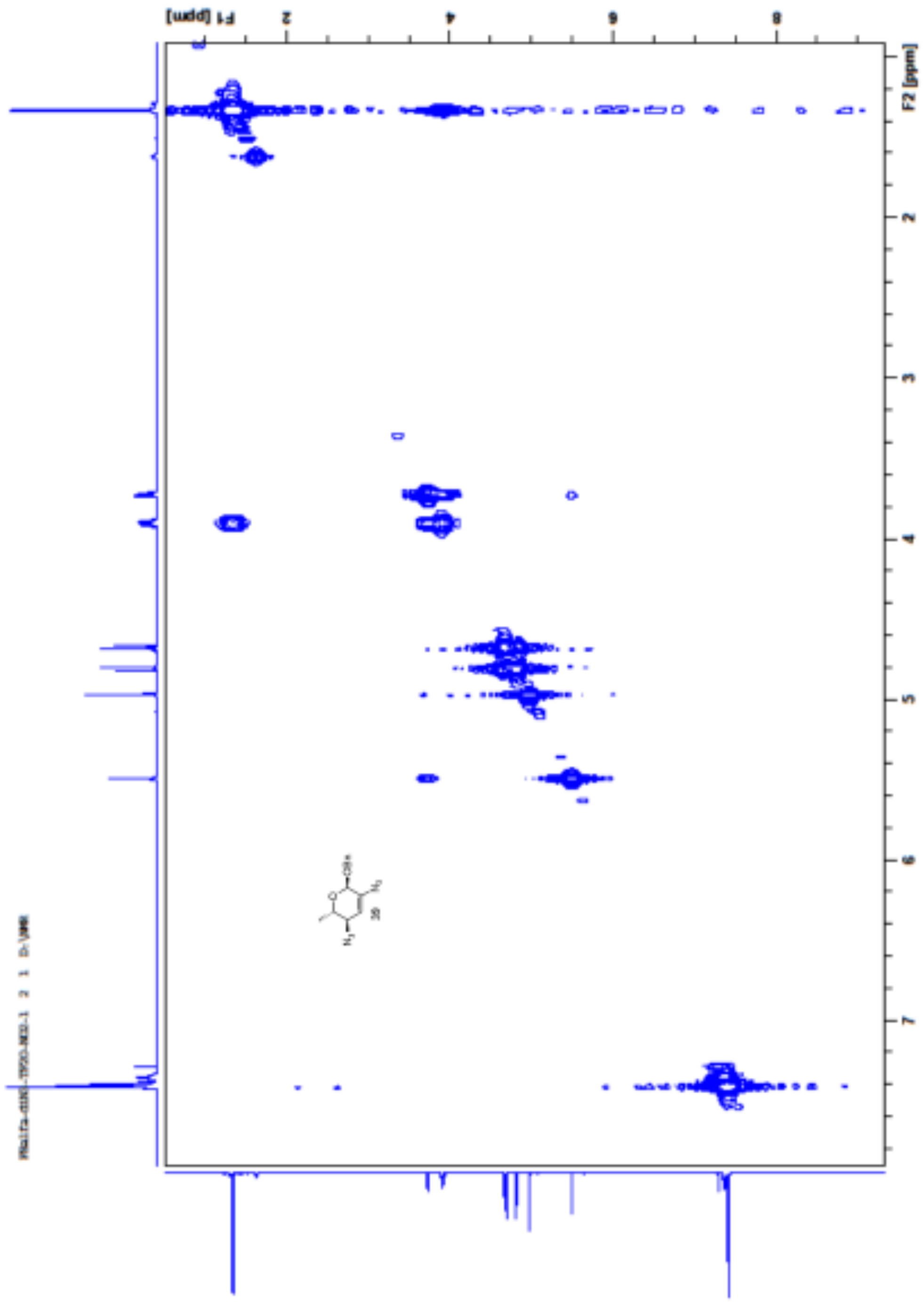

^{13}C NMR DEPT Spectroscopy of Benzyl 2,4-di-azide-2,3,4-tri-deoxy-2-dehydro-α-L-rhamnopyranoside (39)

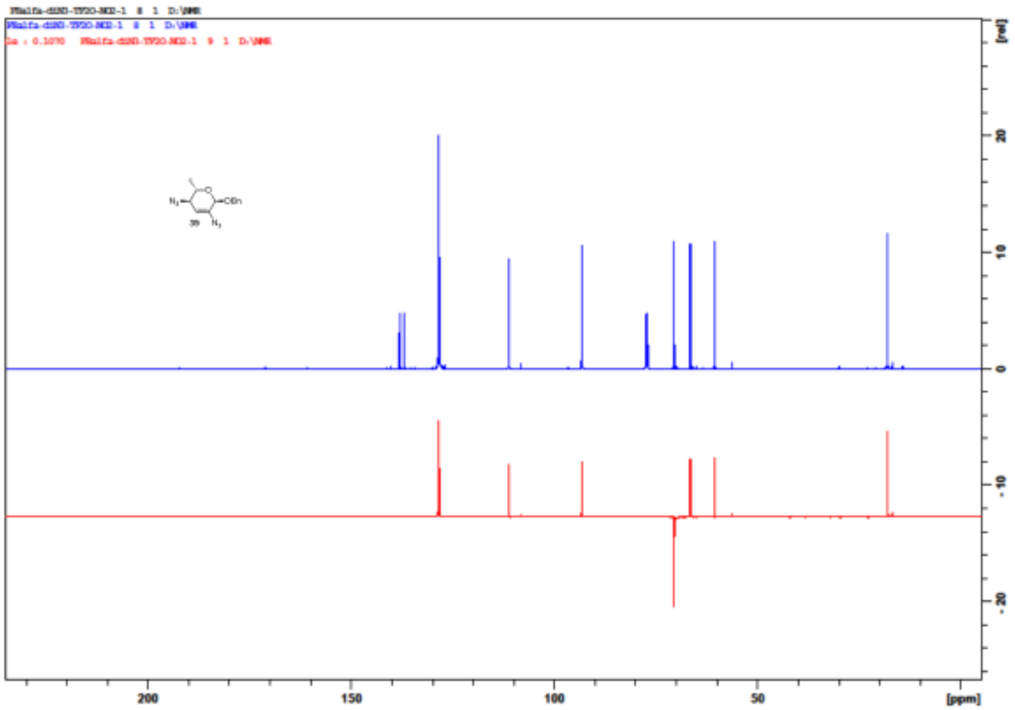

HMBC&HSQC NMR Spectroscopy of Benzyl 2,4-di-azide-2,3,4-tri-deoxy-2-dehydro-α-L- rhamnopyranoside (39)

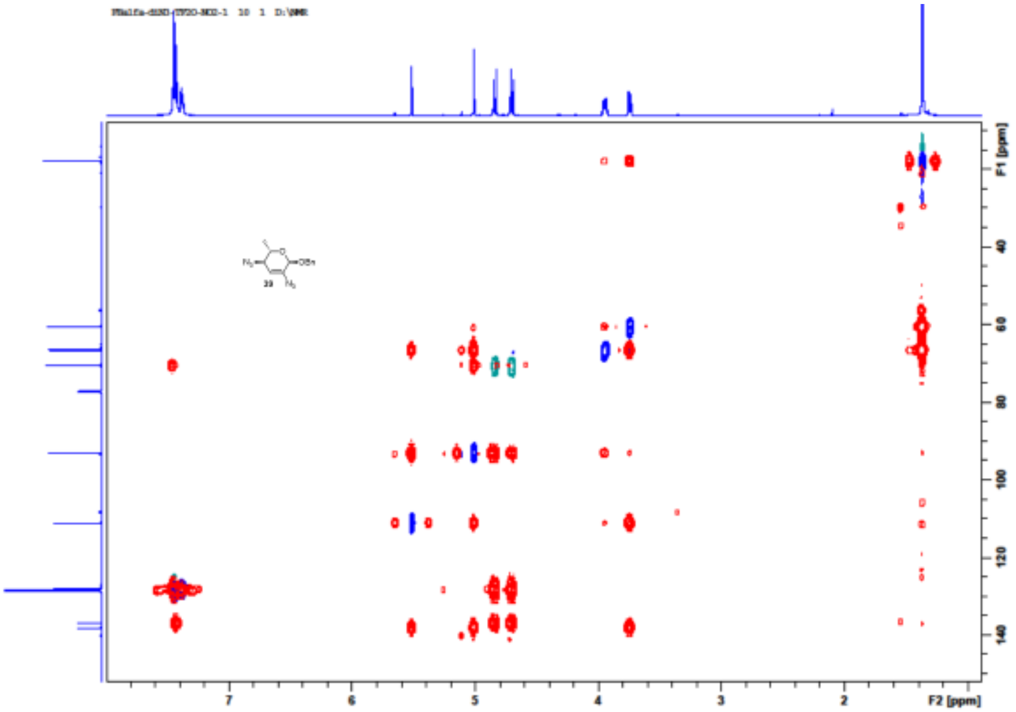

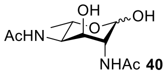

^{1}H NMR Spectroscopy of 2,4-diacetamido-2,4,6-tri-deoxy-L-altropyranoside (40)

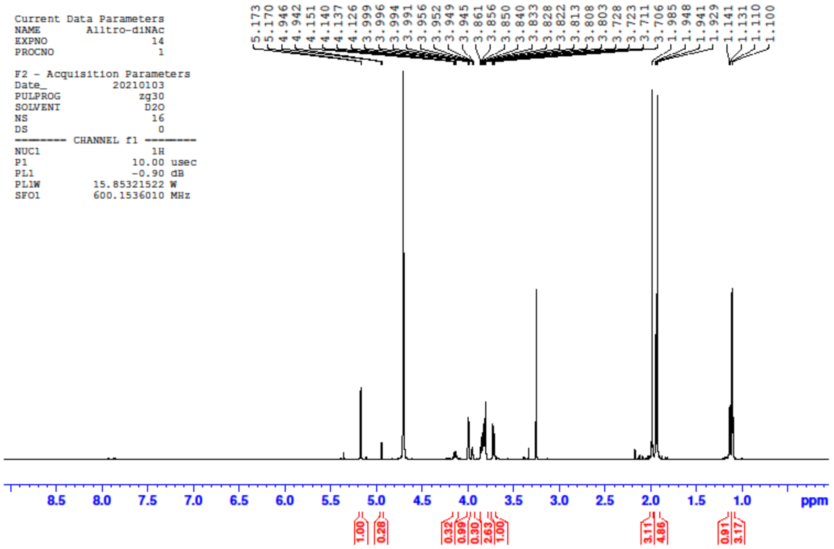

^{13}C NMR Spectroscopy of 2,4-diacetamido-2,4,6-tri-deoxy-L-altropyranoside (40)

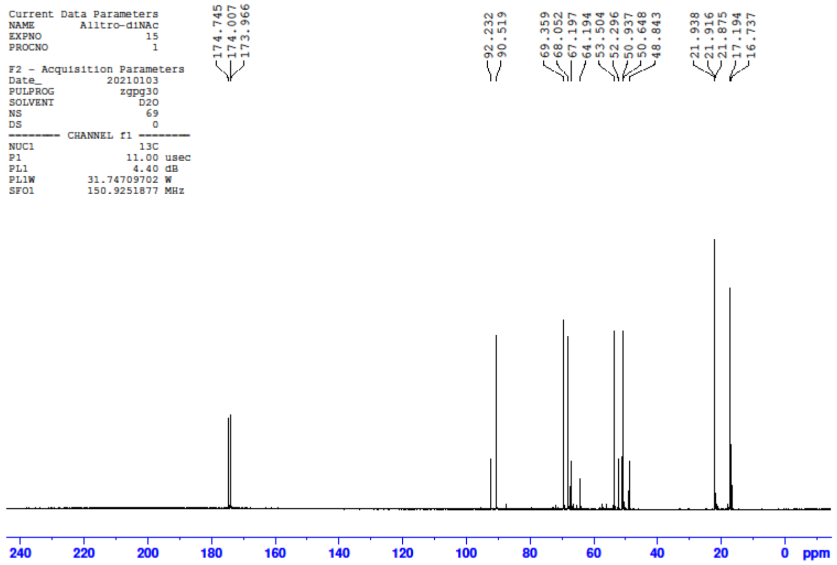

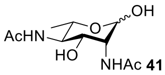

^{1}H NMR Spectroscopy of 2,4-diacetamido-2,4,6-tri-deoxy-L-rhamnopyranoside (41)

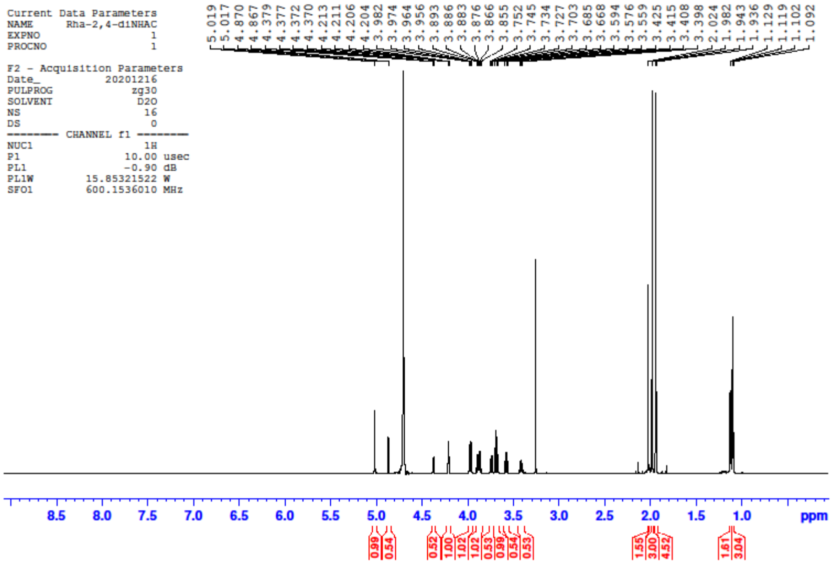

^{13}C NMR Spectroscopy of 2,4-diacetamido-2,4,6-tri-deoxy-L-rhamnopyranoside (41)

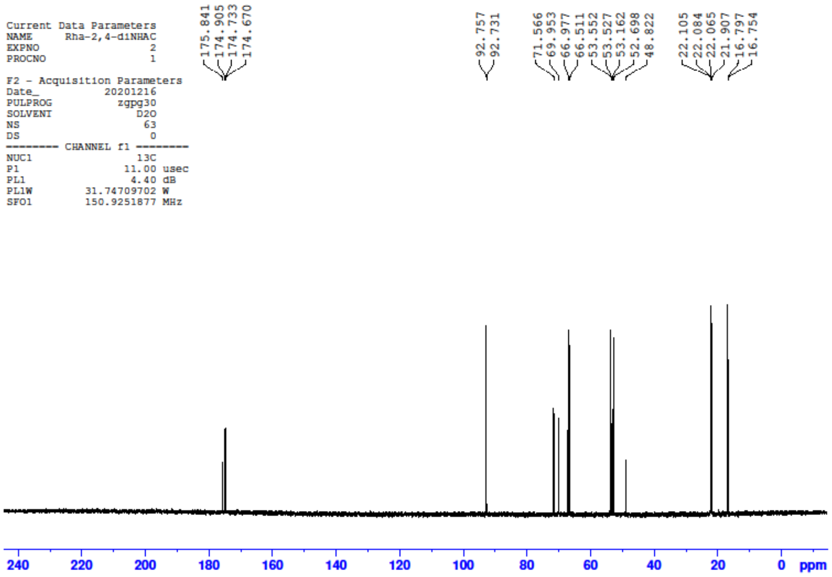

COSY NMR Spectroscopy of 2,4-diacetamido-2,4,6-tri-deoxy-L-rhamnopyranoside (41)

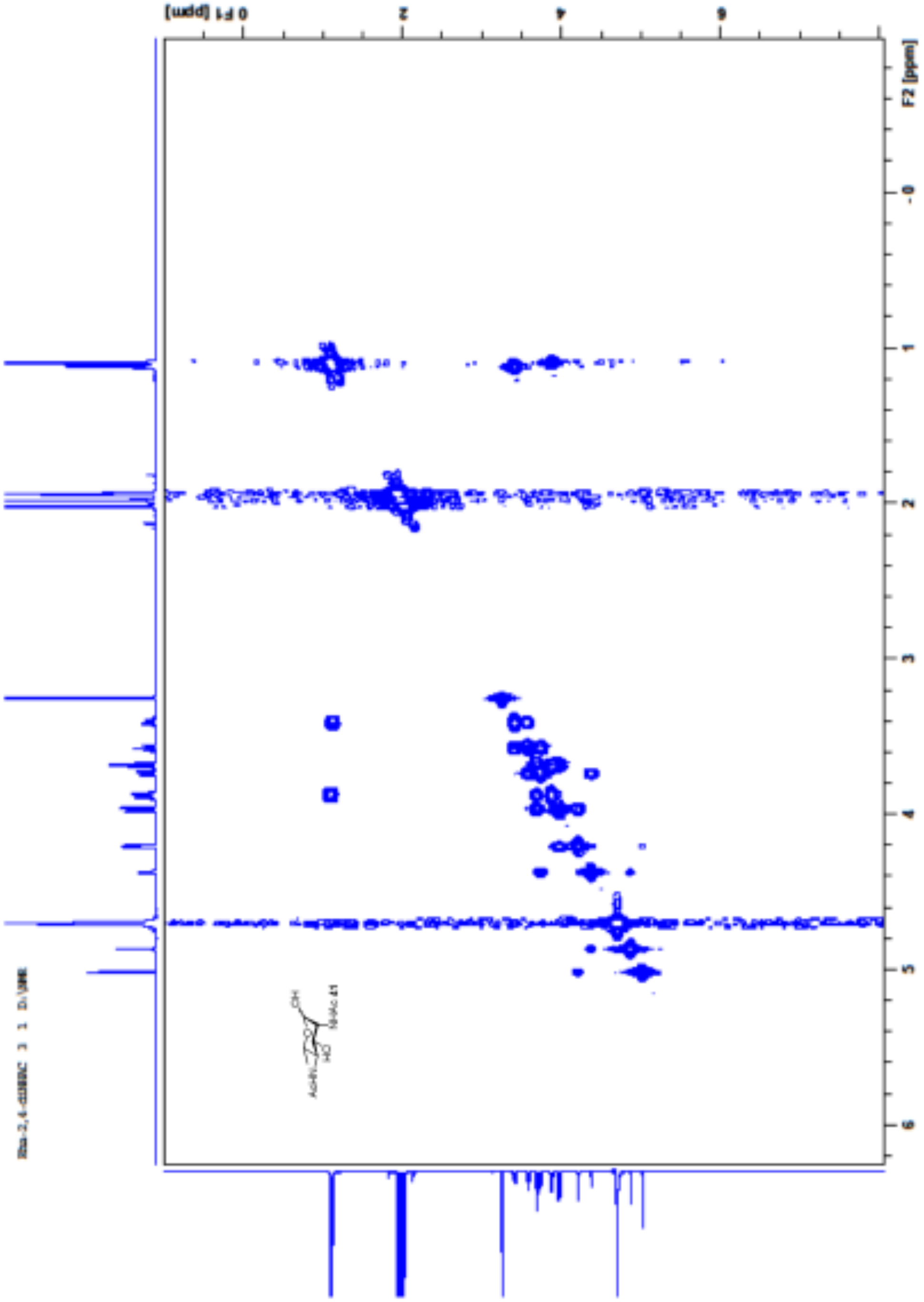

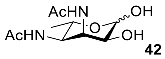

42

^{1}H NMR Spectroscopy of 3,4- di-acetamido-6-deoxyl-α-L-allopyranoside (42)

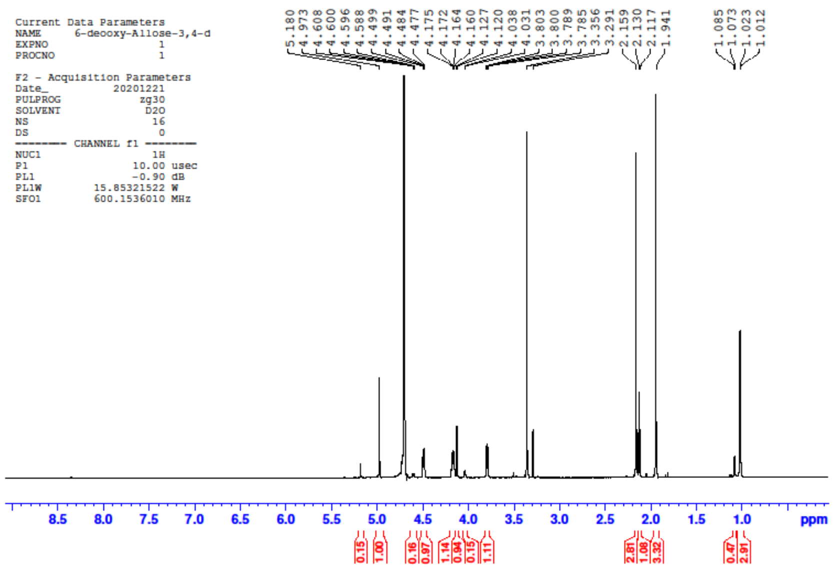

^{13}C NMR Spectroscopy of 3,4- di-acetamido-6-deoxyl-α-L-allopyranoside (42)

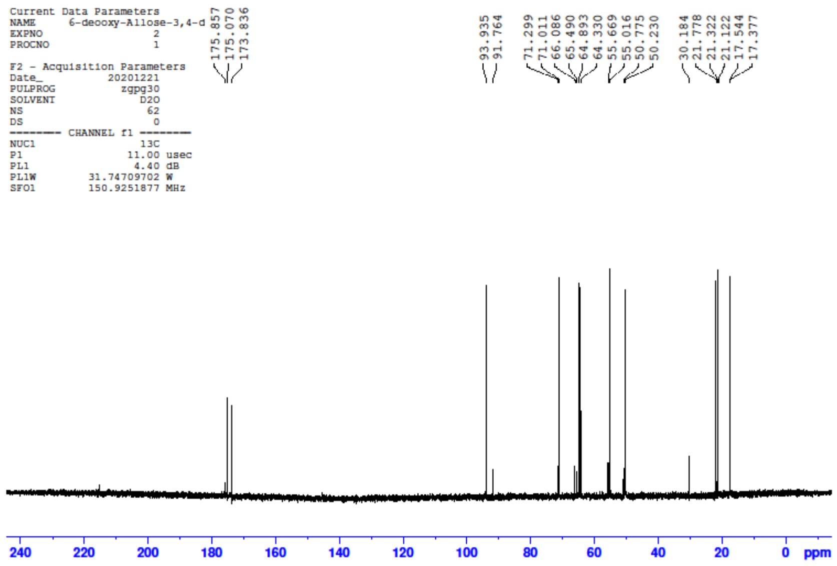

COSY NMR Spectroscopy of 3,4- di-acetamido-6-deoxyl-α-L-allopyranoside (42)

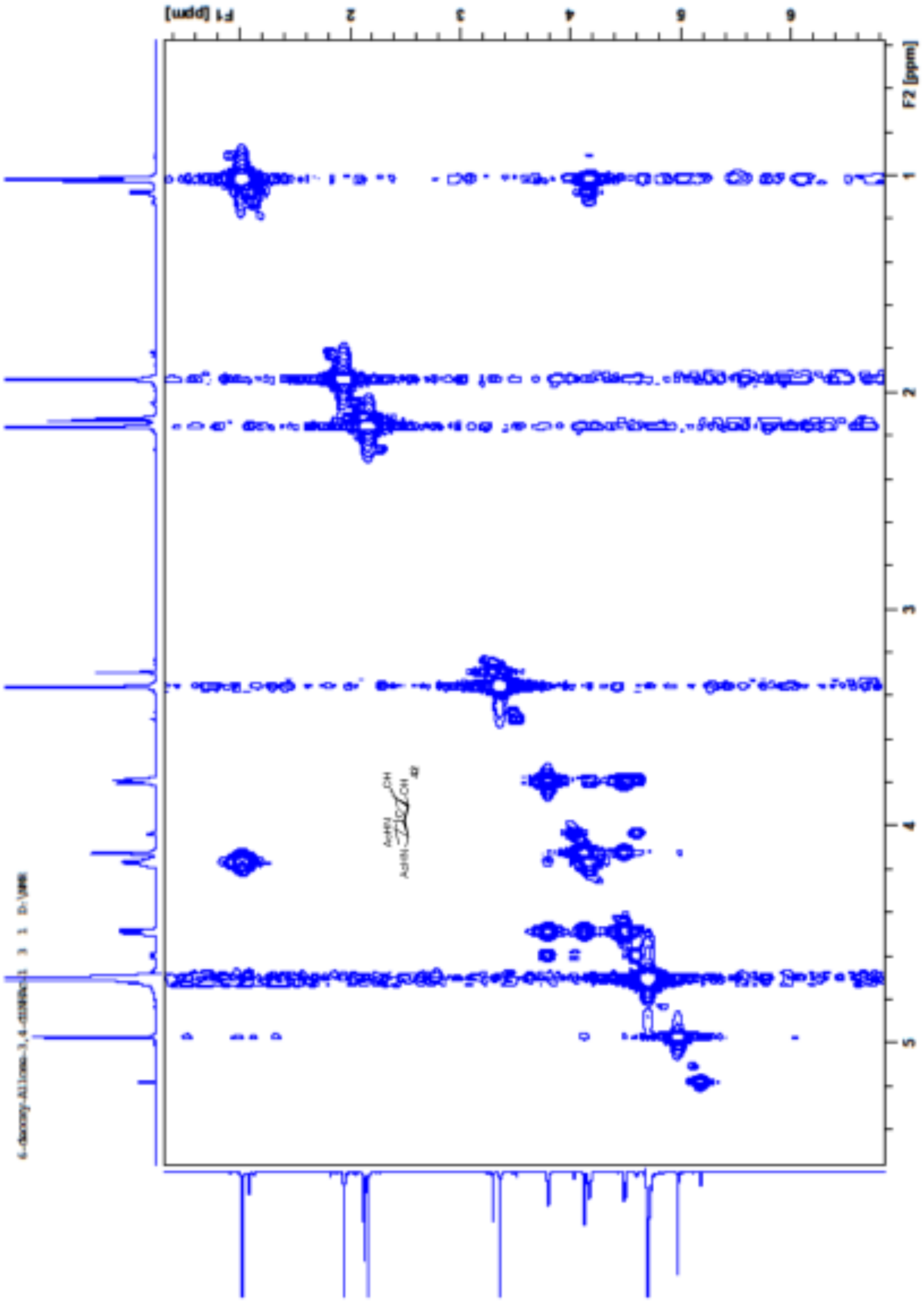

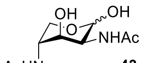

43

^{1}H NMR Spectroscopy of 2,4-di-acetamido-*β*-D-lyxopyranoside (43)

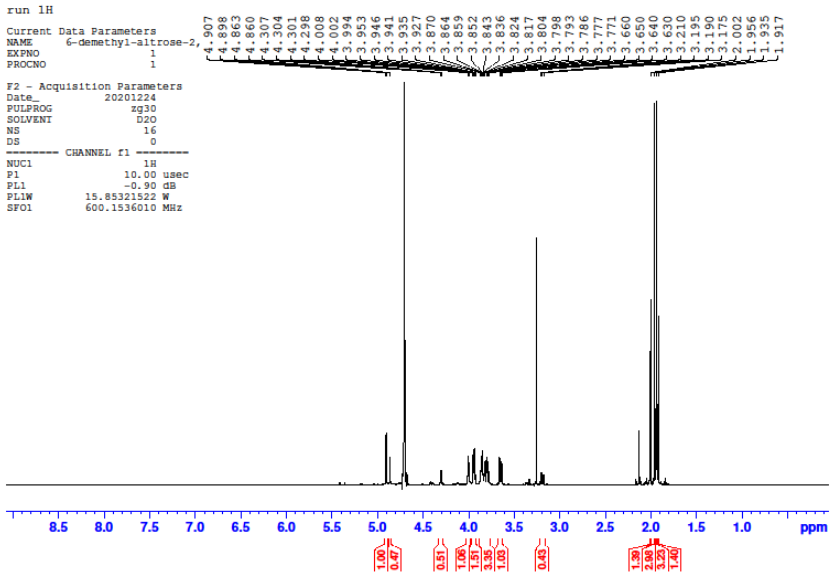

^{13}C NMR Spectroscopy of 2,4-di-acetamido-*β*-D-lyxopyranoside (43)

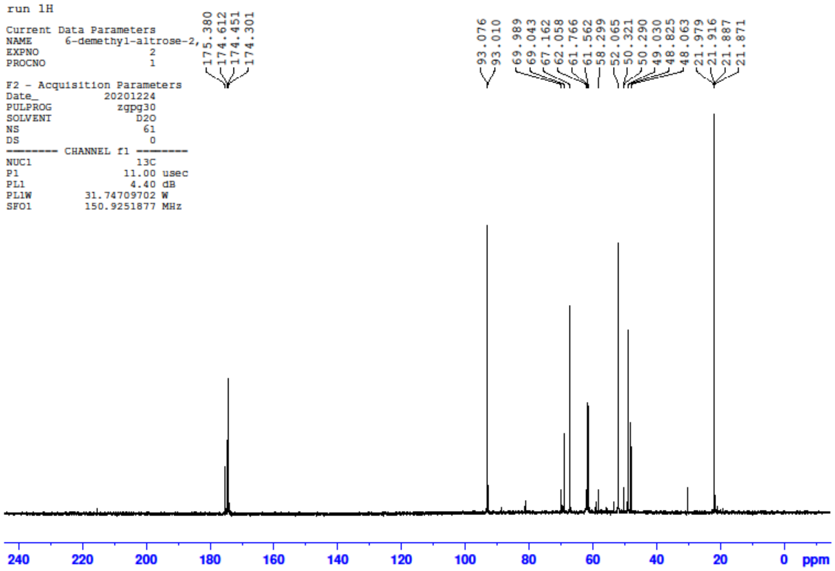

COSY NMR Spectroscopy of 2,4-di-acetamido-β-D-lyxopyranoside (43)

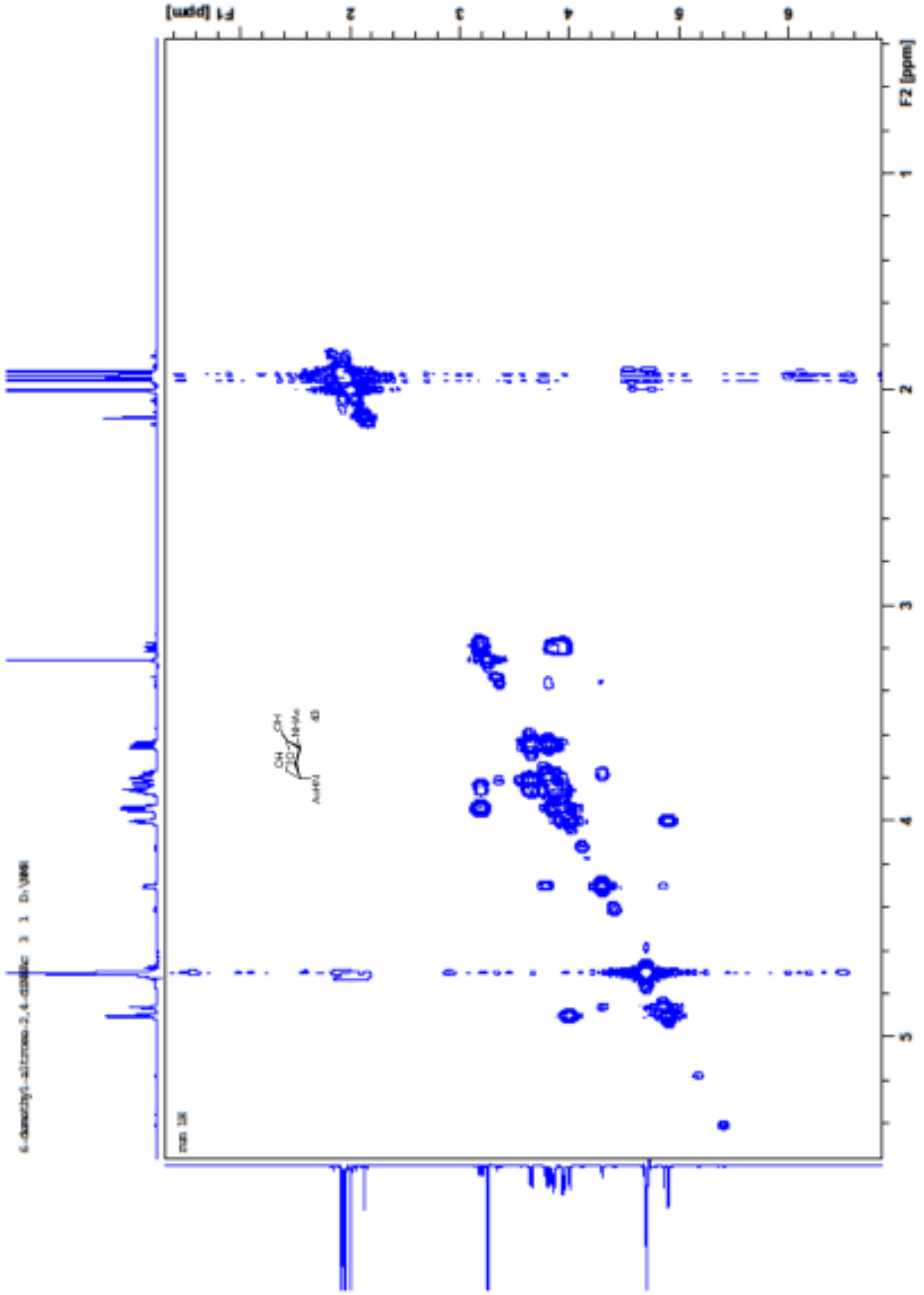

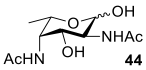

44

^{1}H NMR Spectroscopy of 2,4- di-acetamido-L-fucopyranoside (44)

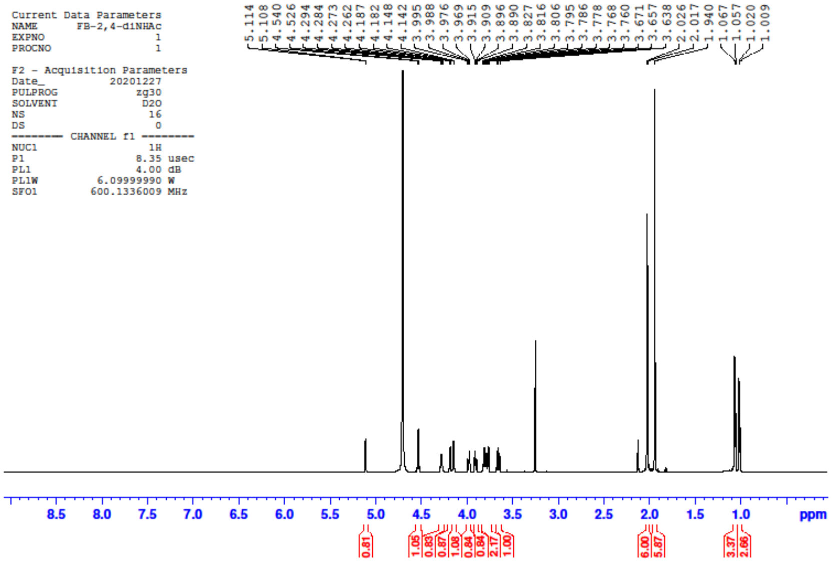

^{13}C NMR Spectroscopy of 2,4- di-acetamido-L-fucopyranoside (44)

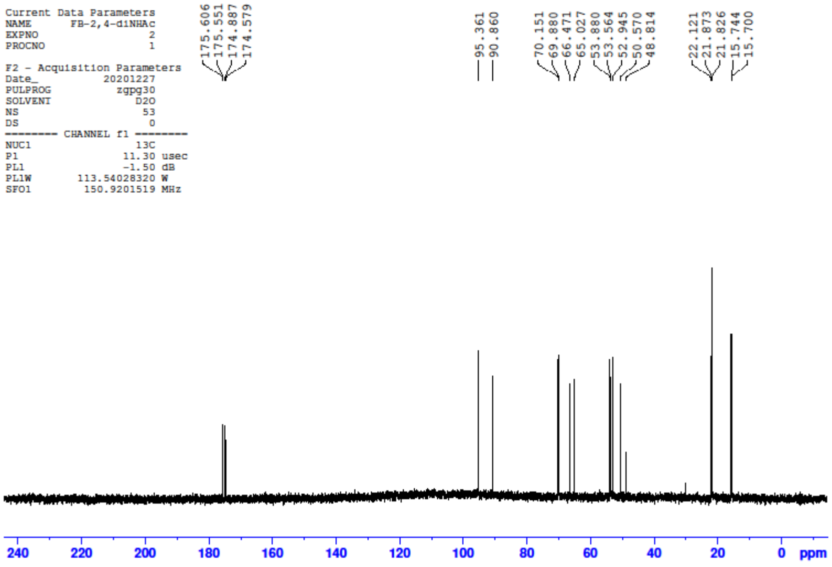

COSY NMR Spectroscopy of 2,4- di-acetamido-L-fucopyranoside (44)

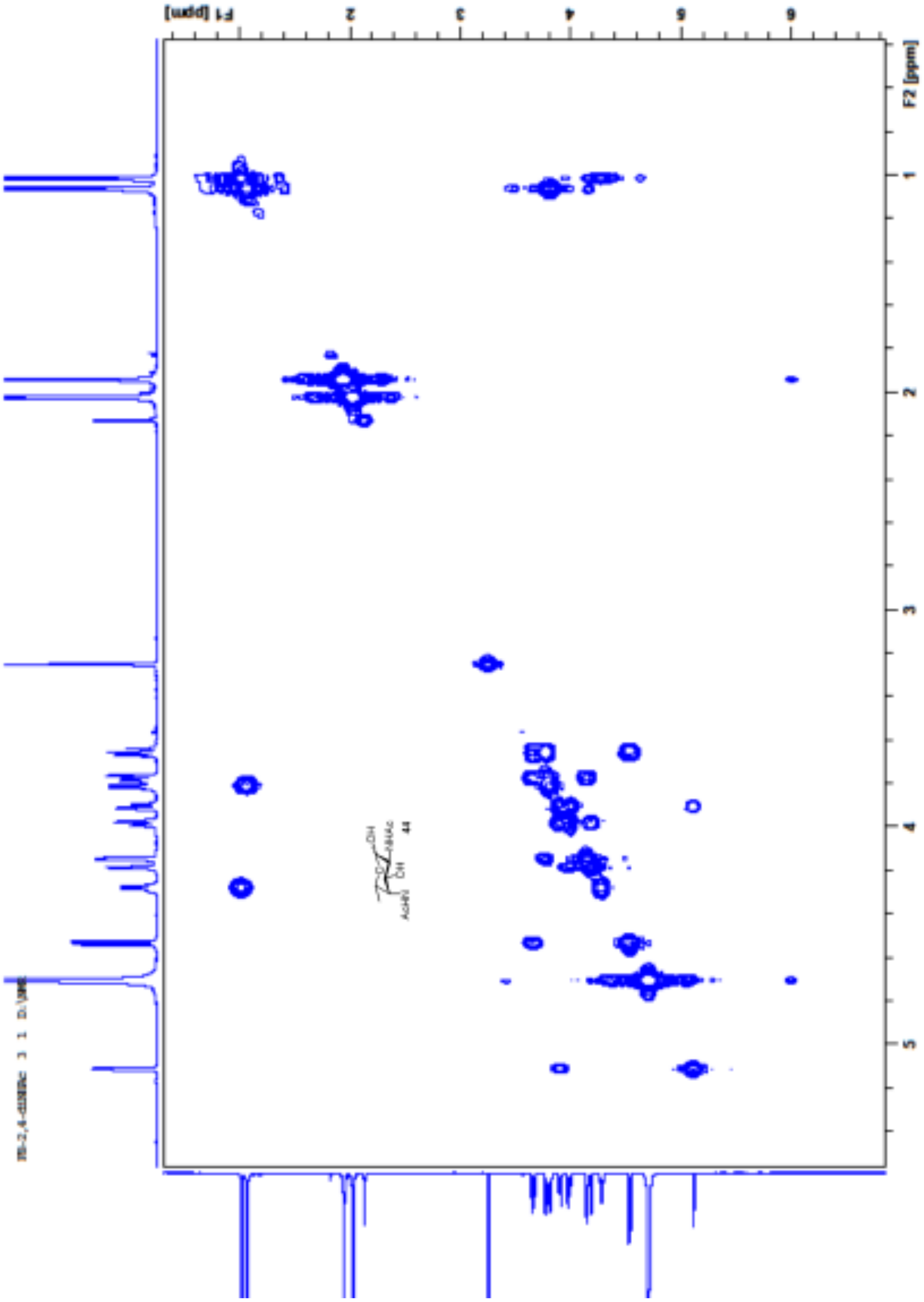

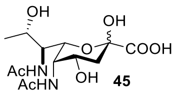

45

^{1}H NMR Spectroscopy of Pseudaminic Acid (Pse5Ac7Ac) (45)

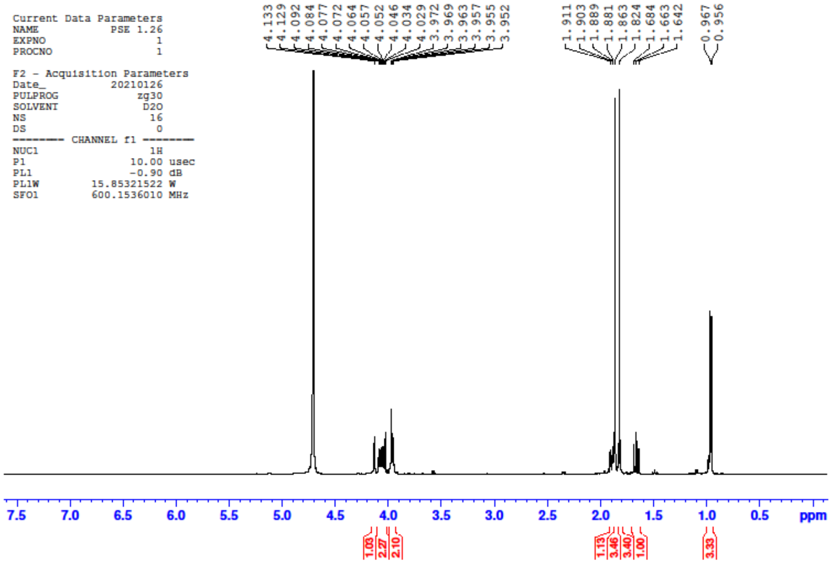

^{13}C NMR Spectroscopy of Pseudaminic Acid (Pse5Ac7Ac) (45)

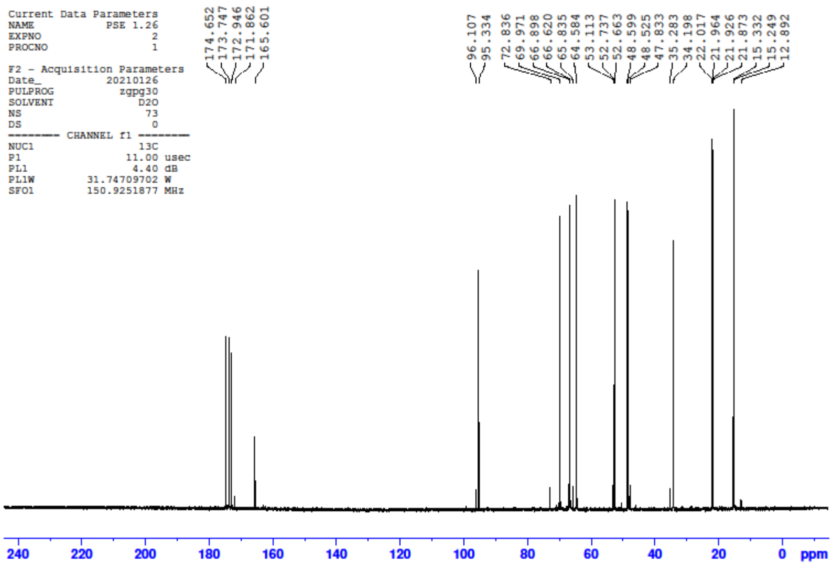

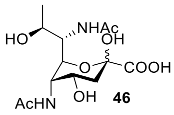

46

^{1}H NMR Spectroscopy of Pseudaminic Acid (Pse5Ac7Ac) isomer (46)

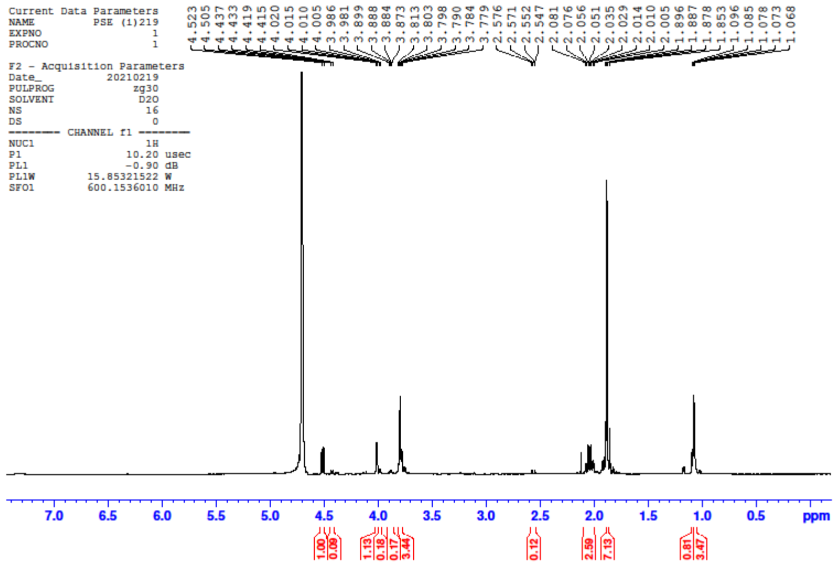

^{13}C NMR Spectroscopy of Pseudaminic Acid (Pse5Ac7Ac) isomer (46)

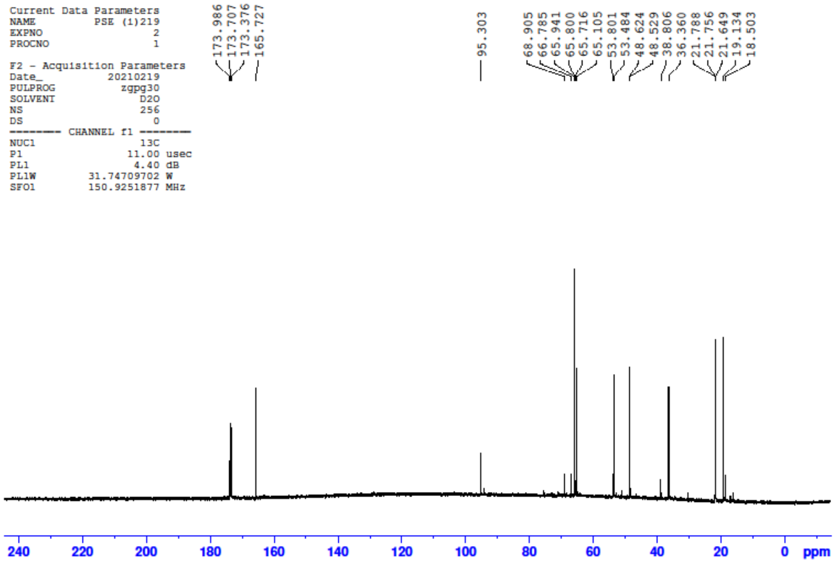

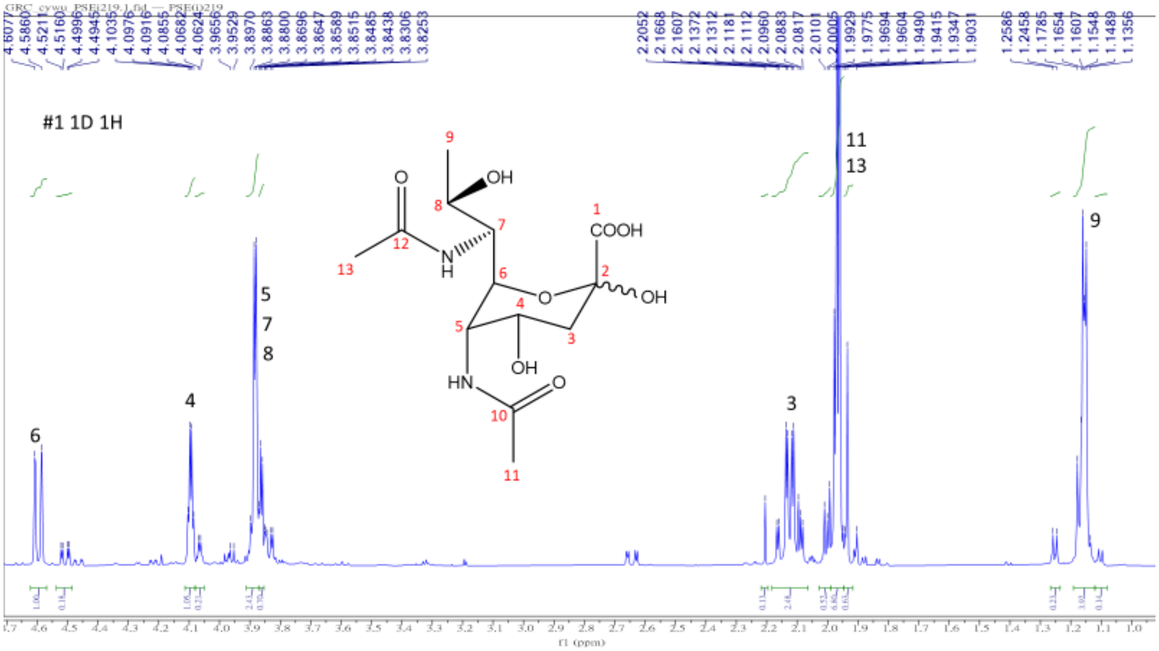
#1 1D 1H
COOH
OH
HN
O
6
4
5
7
8
3
11
13
9
f1 (ppm)

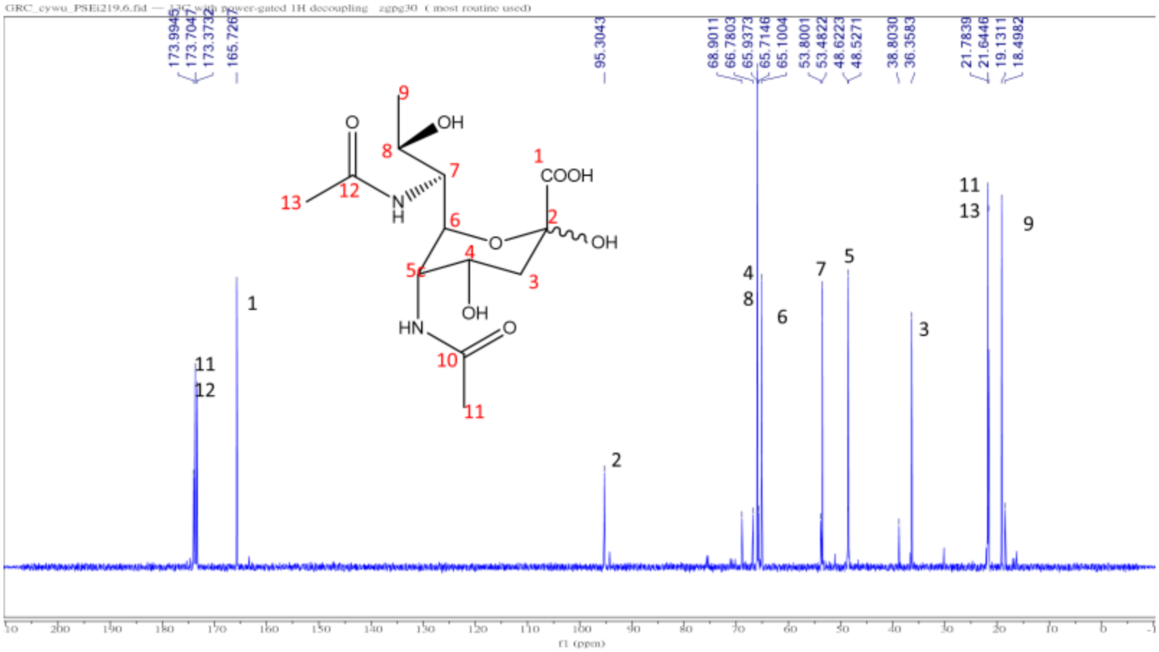
GRC_cywu_PSEi219.6.fid — 13C with power-gated 1H decoupling zgpg30 (most routine used)
173.9945
173.7047
173.3732
165.7267
95.3043
68.9011
66.7803
65.9373
65.7146
65.1004
53.8001
53.4822
48.6223
48.5271
38.8030
36.3583
21.7839
21.6446
19.1311
18.4982
COOH
OH
HN
O
1
2
11
12
4
8
6
7
5
3
11
13
9
f1 (ppm)

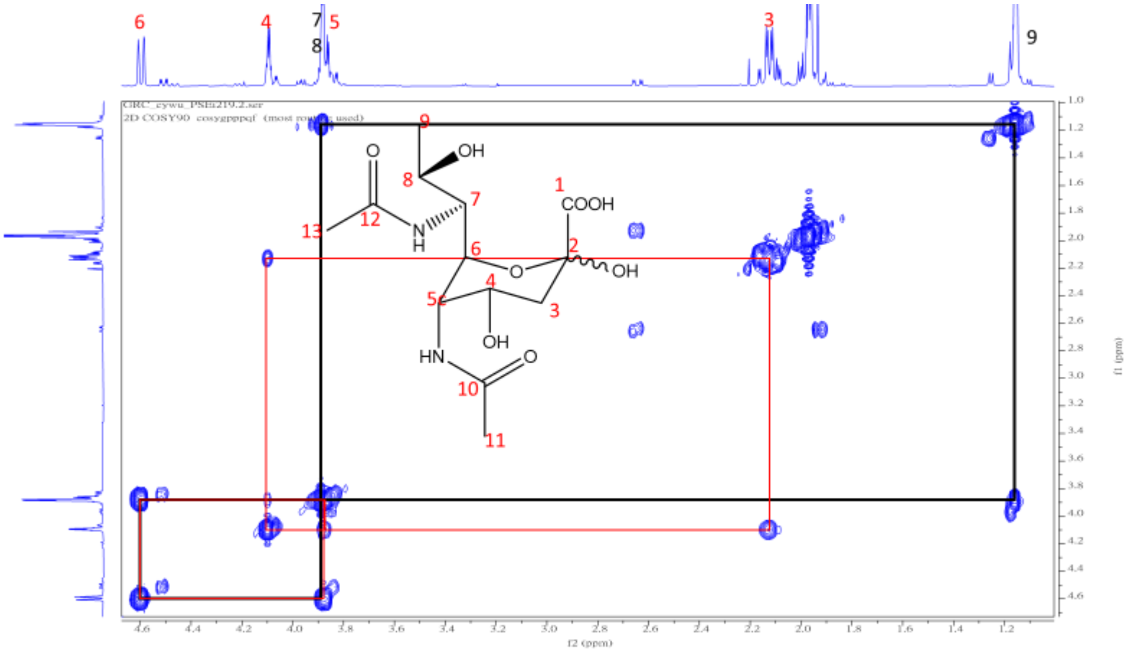

1D selective TOCSY D9:280ms
1D 1H

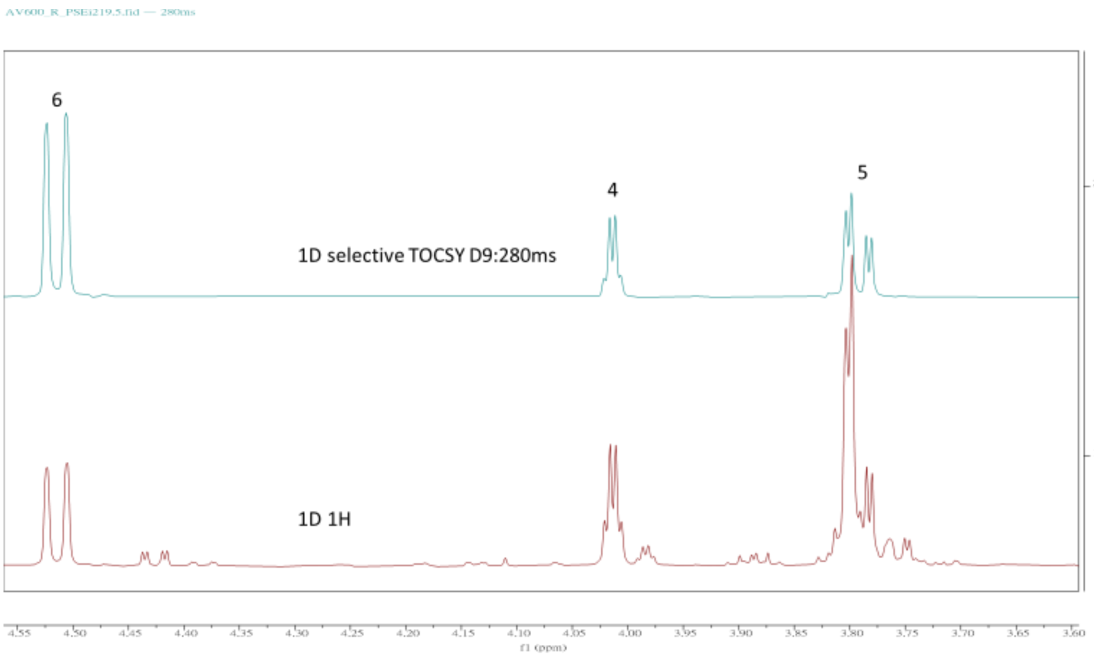

1D selective TOCSY D9:280ms
1D 1H

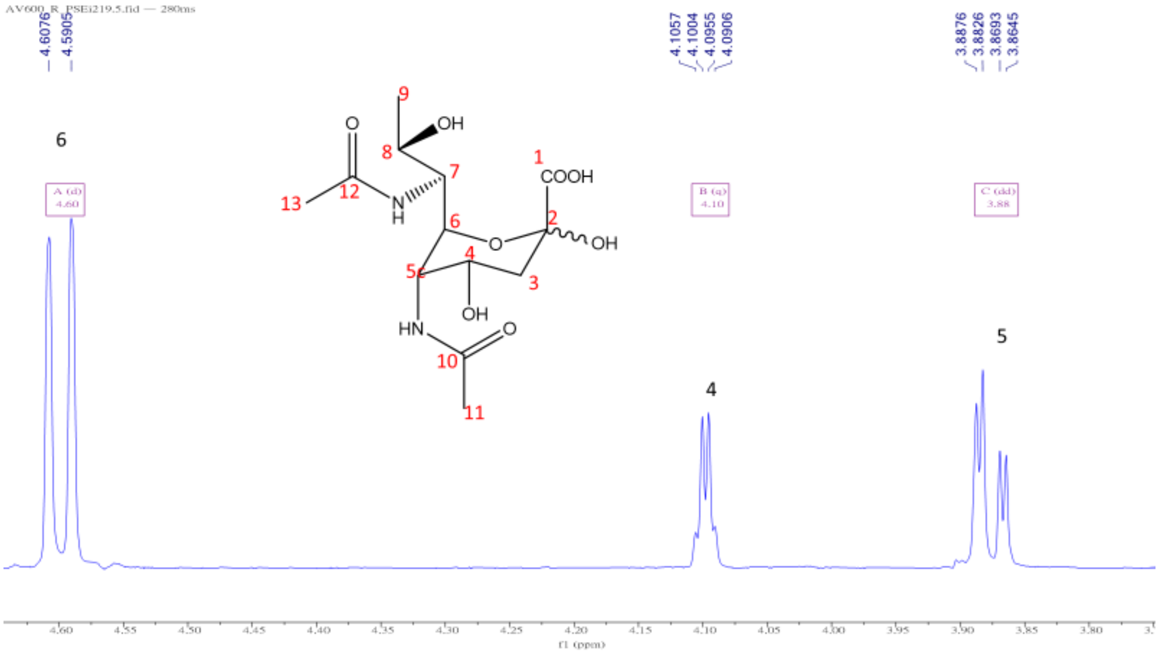
AV600_R_PSEi219.5.fid — 280ms
4.6076
4.5905
4.1057
4.1004
4.0955
4.0906
3.8876
3.8826
3.8693
3.8645
6
4
5
A (d)
4.60
B (q)
4.10
C (dd)
3.88
f1 (ppm)

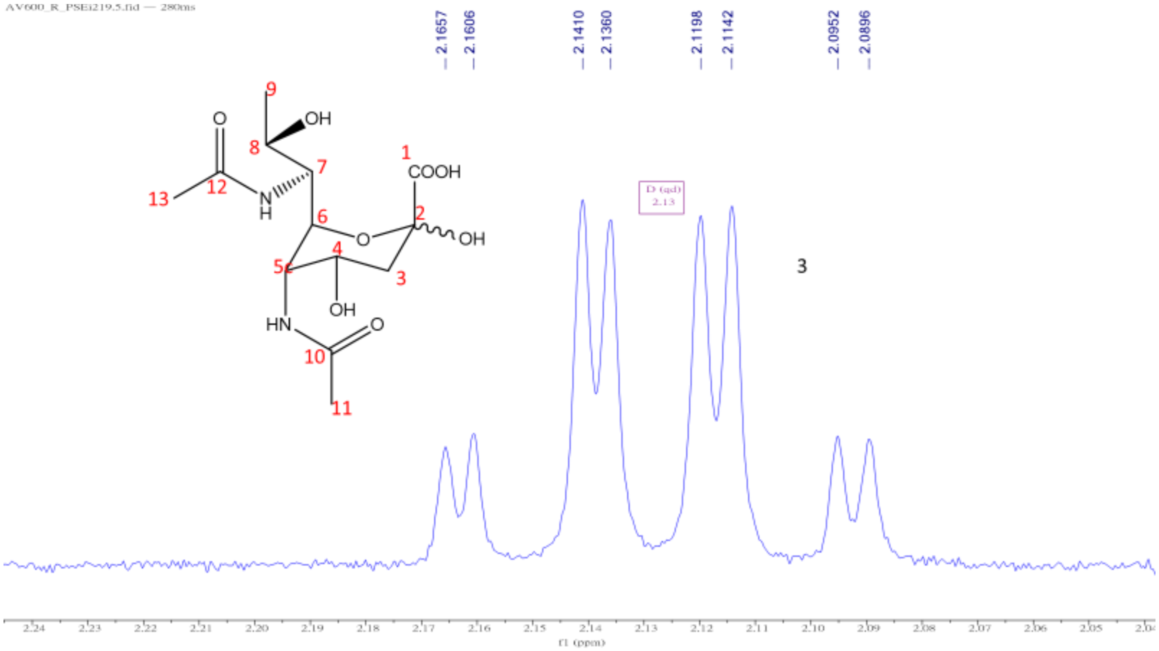
AV600_R_PSEi219.5.fid — 280ms
2.1657
2.1606
2.1410
2.1360
2.1198
2.1142
2.0952
2.0896
D (qd)
2.13
3
f1 (ppm)

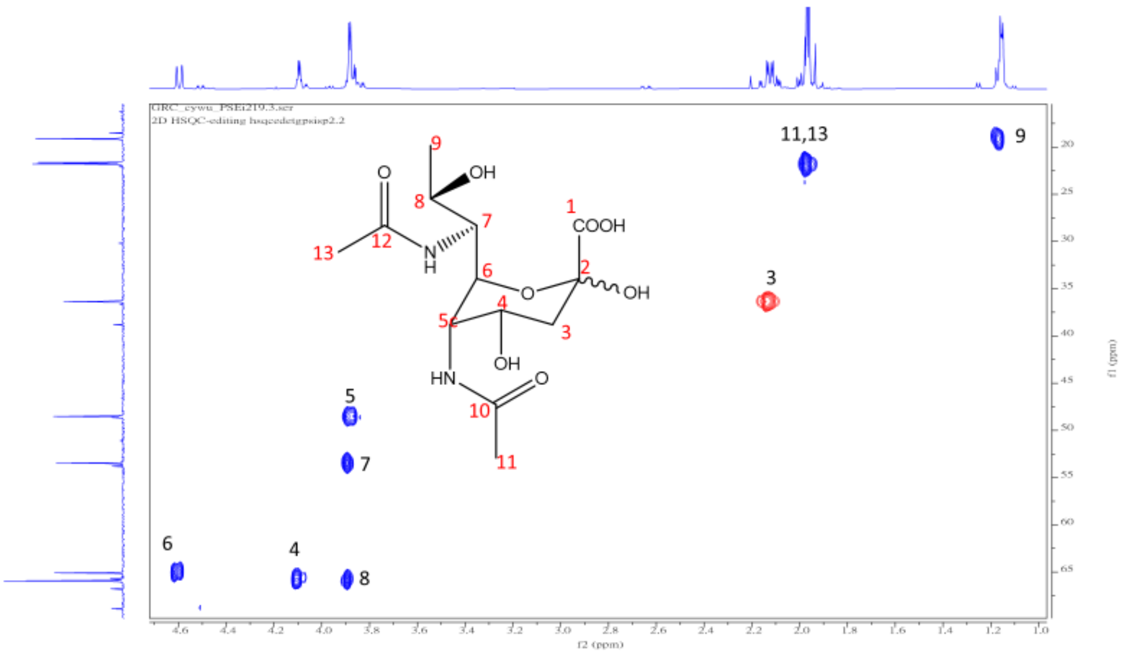
GRC_cywu_PSEi219.3.ser
2D HSQC-editing hsqcedetgpsisp2.2
11,13
9
3
5
7
6
4
8
COOH
OH
O
HN
N
H
f2 (ppm)
f1 (ppm)

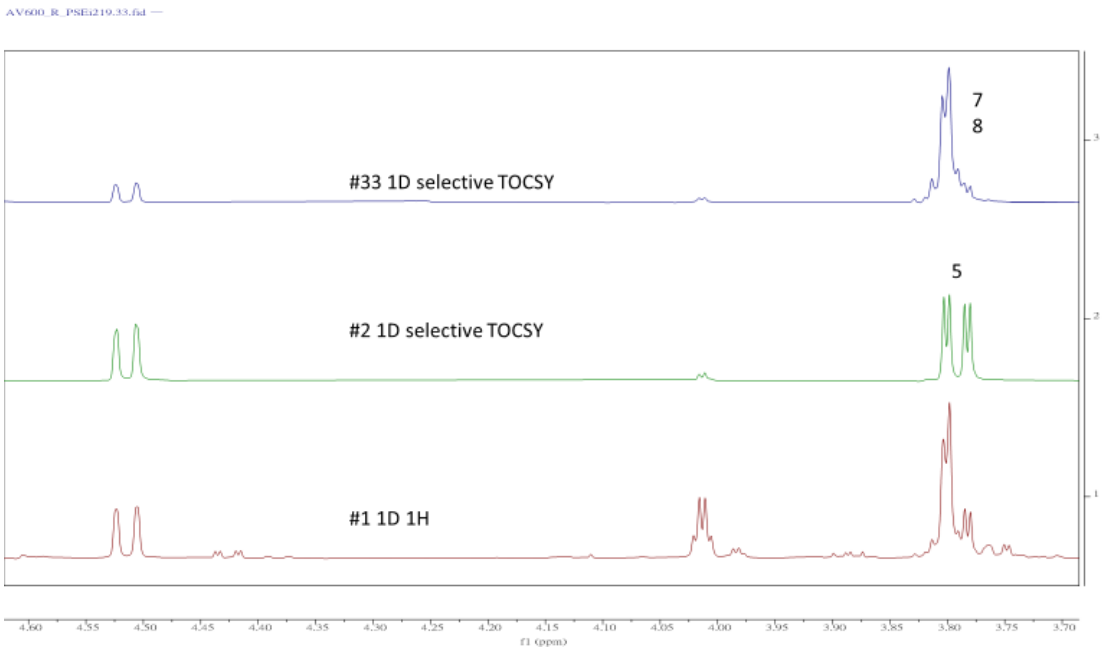
AV600_R_PSEi219.33.fid
#33 1D selective TOCSY
7
8
#2 1D selective TOCSY
5
#1 1D 1H
f1 (ppm)

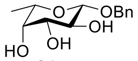

S1

^{1}H NMR Spectroscopy of Benzyl β-L-fucopyranoside (S1)

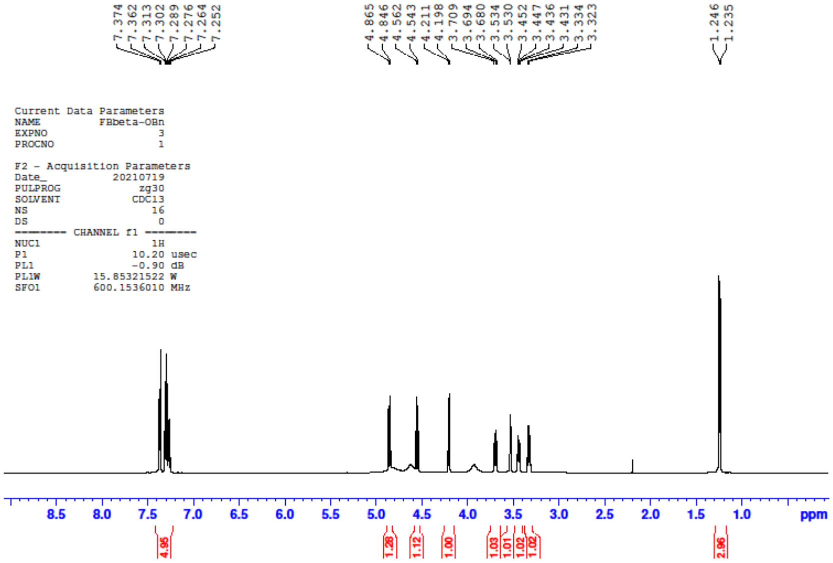

^{13}C NMR Spectroscopy of Benzyl β-L-fucopyranoside (S1)

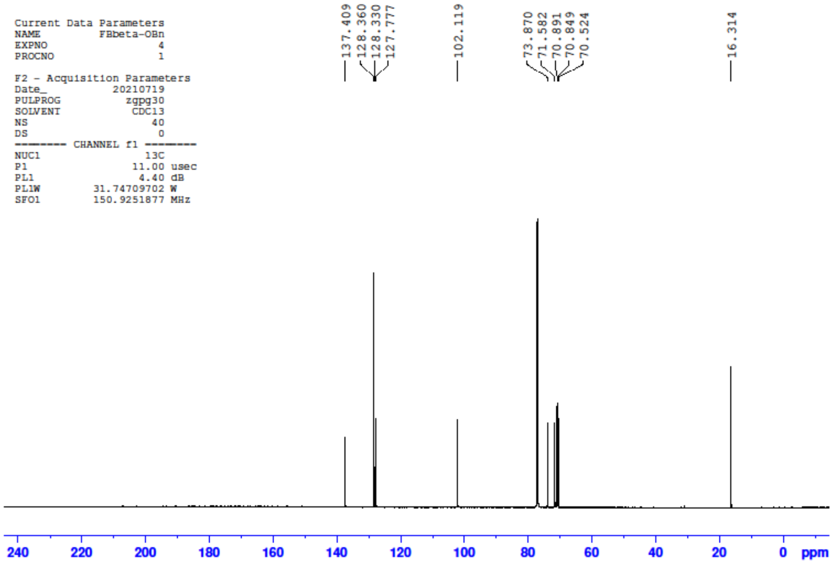

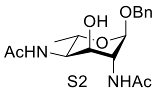

^{1}H NMR Spectroscopy of Benzyl 2,4-diacetamido-2,4,6-trideoxy-*a*-L-altropyranoside (S2)

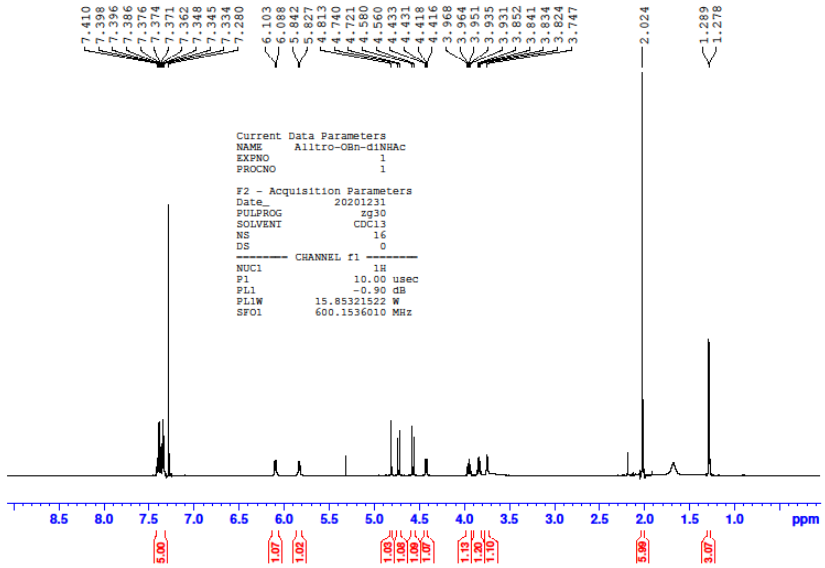

^{13}C NMR Spectroscopy of Benzyl 2,4-diacetamido-2,4,6-trideoxy-*a*-L-altropyranoside (S2)

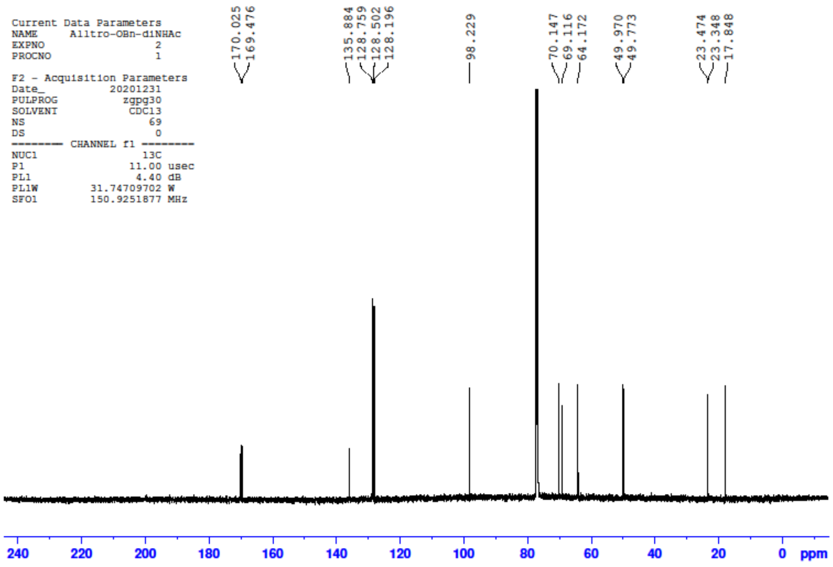

NMR Ratio of Double Inversion Competed with NGP

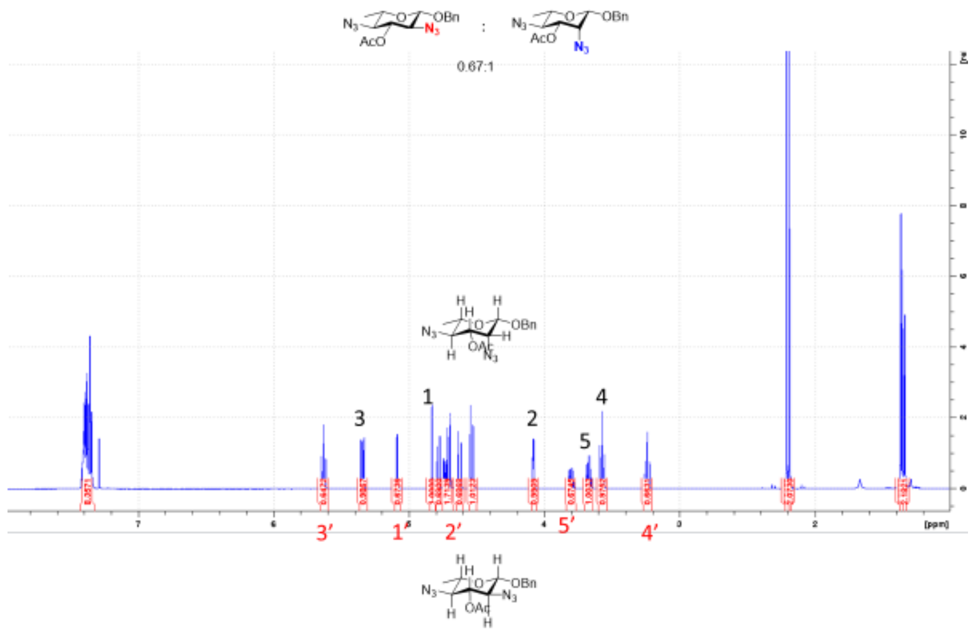

Figure S1 double inversion competed with NGP for compound **1 (Entry 1)**

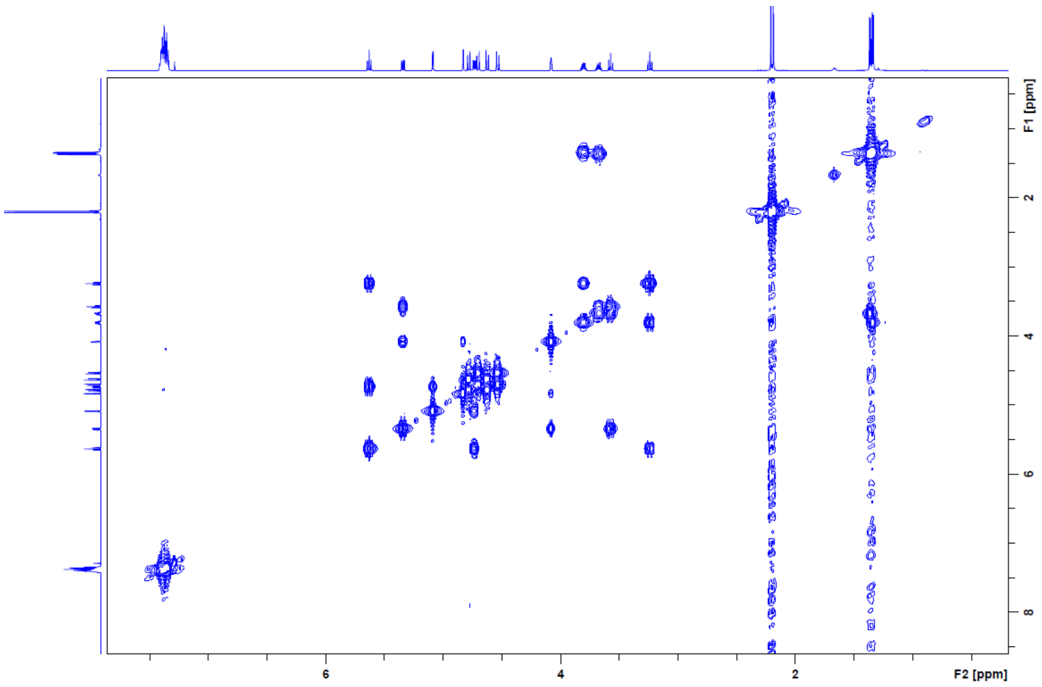

Figure S2 the COSY NMR spectra of compound **2** and **3** as a mixture

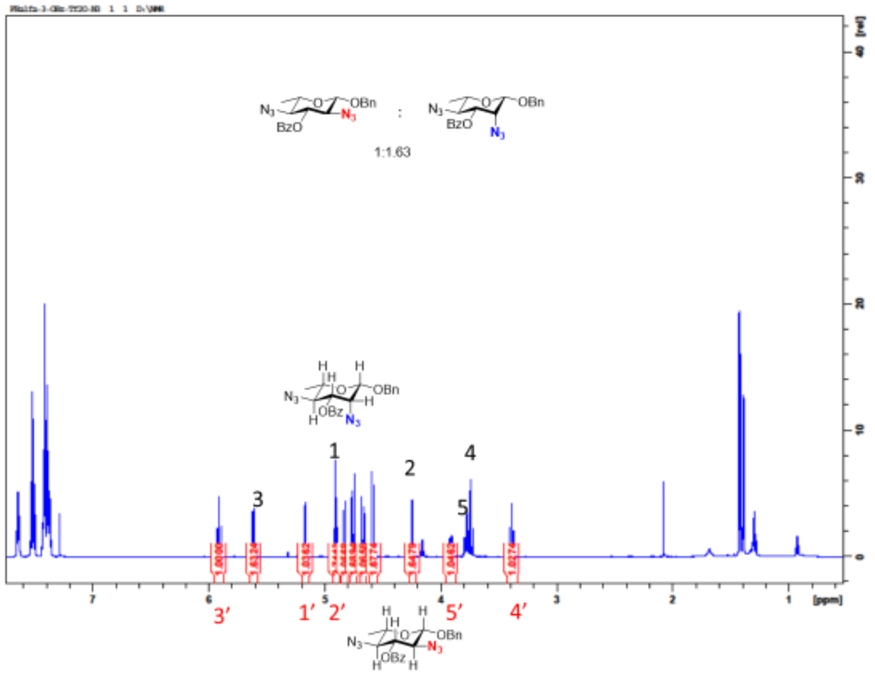

Figure S3 double inversion competed with NGP for compound **4 (Entry 3)**

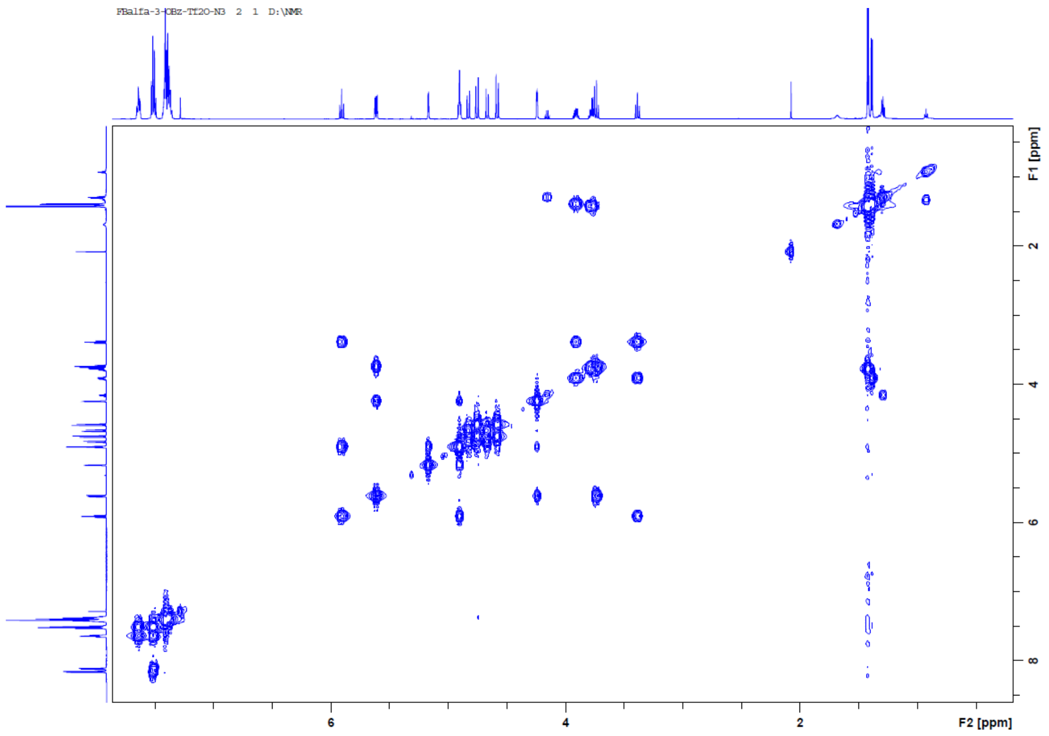

Figure S4 the COSY NMR spectra of compound **5** and **6** as a mixture

Reference:

Brito-Arias, M., Durán-Páramo, E., Mata, I., and Molins, E. (2002). A comparative analysis of mono-and disaccharide benzyl fucopyranosides. *Acta Crystallographica Section C: Crystal Structure Communications* 58(9), o537-o539.
